# 50 Years of Organoselenium Chemistry, Biochemistry and Reactivity: Mechanistic Understanding, Successful and Controversial Stories

**DOI:** 10.1002/chem.202403003

**Published:** 2024-11-20

**Authors:** Andrea Madabeni, Marco Bortoli, Pablo A. Nogara, Giovanni Ribaudo, Marco Dalla Tiezza, Leopold Flohé, João B. T. Rocha, Laura Orian

**Affiliations:** ^1^ Dipartimento di Scienze Chimiche Università degli Studi di Padova Via Marzolo 1 35131 Padova Italy; ^2^ Department of Chemistry and Hylleraas Centre for Quantum Molecular Sciences University of Oslo Oslo 0315 Norway; ^3^ Instituto Federal de Educação, Ciência e Tecnologia Sul-rio-grandense (IFSul) Av. Leonel de Moura Brizola, 2501 96418-400 Bagé, RS Brasil; ^4^ Dipartimento di Medicina Molecolare e Traslazionale Università degli Studi di Brescia Viale Europa 11 25123 Brescia Italy; ^5^ Department of Molecular Medicine University of Padova Italy; ^6^ Departamento de Bioquímica Universidad de la República Montevideo Uruguay; ^7^ Departamento de Bioquímica *Universidade Federal*do Rio Grande do Sul (UFRGS) 90035-003 Porto Alegre, RS Brazil

**Keywords:** Glutathione peroxidase, Organoselenide, Oxidation, Selenium, Selenoneine

## Abstract

In 1973, two major discoveries changed the face of selenium chemistry: the identification of the first mammal selenoenzyme, glutathione peroxidase 1, and the discovery of the synthetic utility of the so‐called selenoxide elimination. While the chemical mechanism behind the catalytic activity of glutathione peroxidases appears to be mostly unveiled, little is known about the mechanisms of other selenoproteins and, for some of them, even the function lies in the dark. In chemistry, the capacity of organoselenides of catalyzing hydrogen peroxide activation for the practical manipulation of organic functional groups has been largely explored, and some mechanistic details have been clearly elucidated. As a paradox, despite the long‐standing experience in the field, the nature of the active oxidant in various reactions still remains matter of debate. While many successes characterize these fields, the pharmacological use of organoselenides still lacks any true application, and while some organoselenides were found to be non‐toxic and safe to use, to date no therapeutically approved use was granted. In this review, some fundamental and chronologically aligned topics spanning organoselenium biochemistry, chemistry and pharmacology are discussed, focusing on the current mechanistic picture describing their activity as either bioactive compounds or catalysts.

## Graphical abstract

From the stink to the stars! The reputation of organoselenium species changed drastically in the past fifty years. While at the beginning of their history they were notoriously famous for their toxicity and infernal smell, organoselenides are nowadays recognized for their fundamental role in redox biology and as green organocatalysts. While applications in pharmaceutical sciences are still lacking, safe‐to‐be‐administered compounds exist and new venues for bioactive low molecular weight organoselenides are being explored. In this review, the pervasive chemistry of the element of the moon is discussed focusing on the pivotal role of organoselenium species in biology, catalysis and pharmacology, providing an historical perspective on different but intertwined fields.

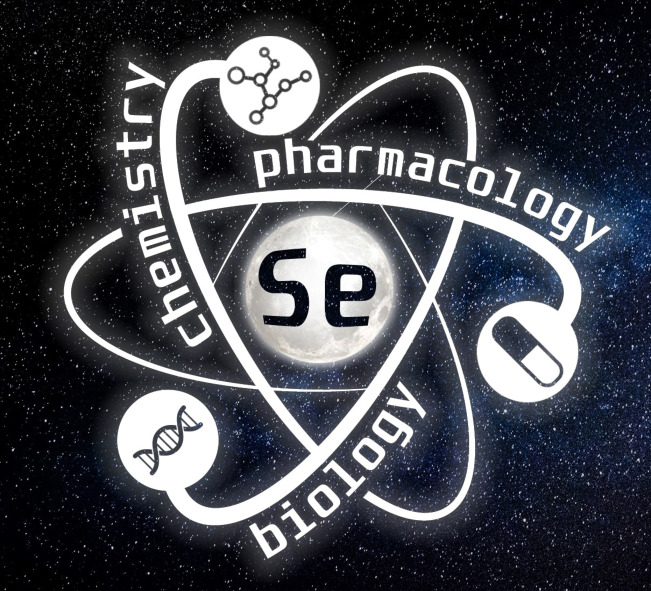

## 1 Introduction

In 1818, Jöns Jacob Berzelius wrote a letter to his colleague Claude Louis Berthollet which among other informed of *“… the discovery of a metallic substance, the oxide of which is a new fixed alkali, and that of another acidifiable metallic substance, more analogous to sulfur, than to any other substance …”*.^[1]^ Two new substances were discovered in Berzelius’ lab during the previous year. The former the scientist refers to is what he will name *lithium*. But it is in the latter that the present review article is focused, due to its important role in biology, chemistry, and pharmacology. The discovery of the new substance was, as in many other cases, accompanied by a fair dose of serendipity: a reddish precipitate was isolated in a sulfuric acid factory. After a description of the appearance and physical properties of the new material, Berzelius strongly refuted the possibility that the substance contained in the red mass is tellurium. Nonetheless, its evident and strong relationship with tellurium prompted the scientist to choose its name: “However, to recall the relationship of the latter with tellurium, I named it *selenium*.”^[1]^ In 1819, the first publication in a scientific journal on selenium appeared in the Swedish *Afhandlingar i Fysik, Kemi och Mineralogi* (Theses in Physics, Chemistry and Mineralogy) and the history of selenium began,^[1]^ a history which is still far from being complete.

**Figure 1:**
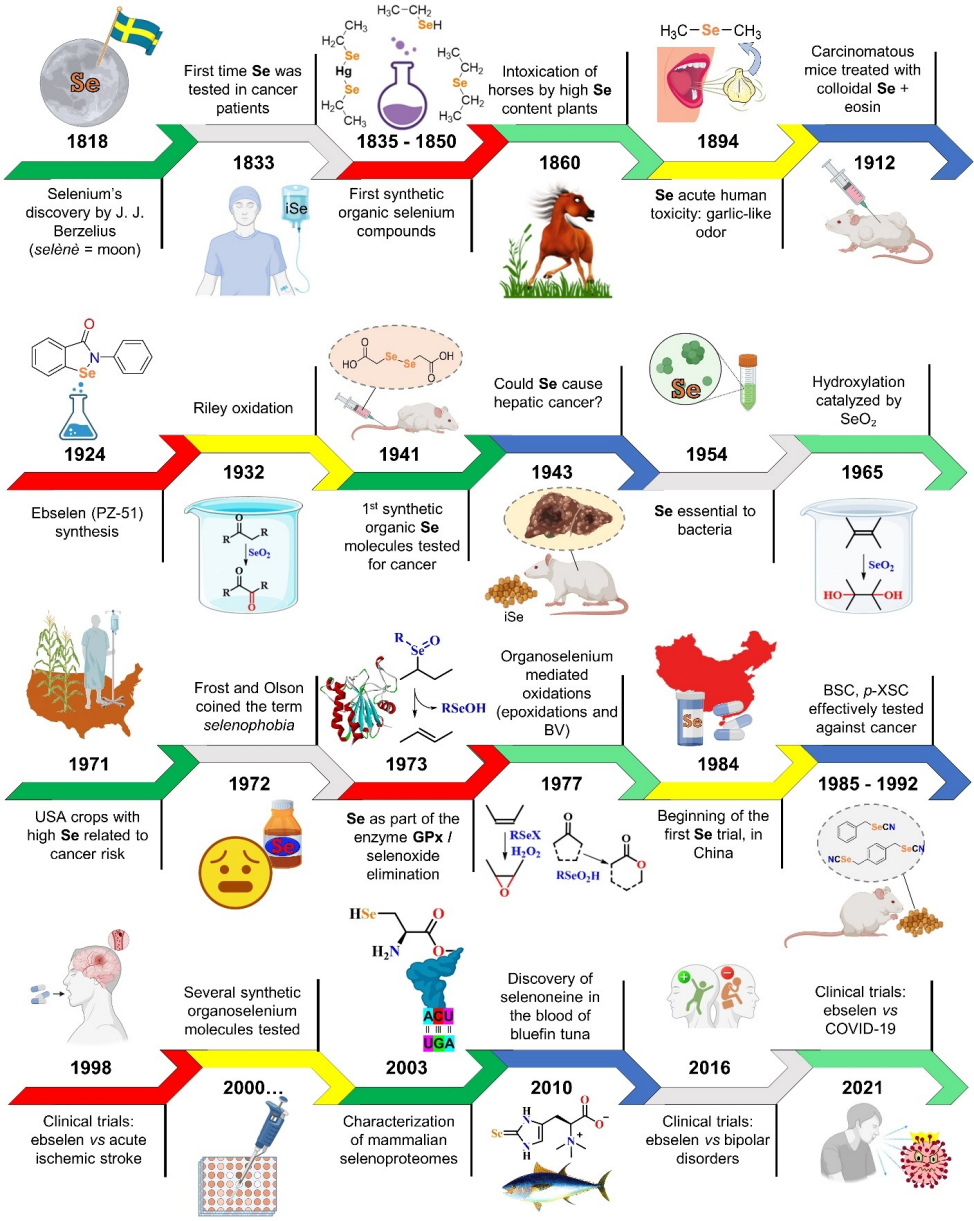
Timeline of selenium biology, chemistry and pharmacology. The main scientific events related to selenium are highlighted.

In the span of two hundred years, the face and shape of selenium chemistry changed, from poison to micronutrient, from smelly toxicant to green catalyst and useful synthetic tool for functional groups manipulation, and toward a possible pharmacological agent (Figure 1).

Most of these evolutions were granted by the birth and growth of the field of organoselenium chemistry, i. e., the field of study of compounds with a carbon–selenium bond (C−Se), which are pervasive in the biological, catalytic and pharmacological context. The role of selenium in these three areas has been explored by different researchers, including enzymologists, organic and medicinal chemists. Due to the diversity in methods and languages employed by scientists across different communities, most of these topics already received detailed reviews in the past. Particularly, the synthetic utility of organoselenium chemistry has been reviewed as early as in the late 70s and early 80s,[^2^, ^3^] and then two decades later to highlight green and catalytic aspects.[^4^, ^5^, ^6^, ^7^] The key role of selenium in biology has been previously reviewed in detail, with a focus on both its function in proteins[^8^, ^9^, ^10^, ^11^] as well as on the application of organoselenides as pharmaceutical agents.[^12^, ^13^, ^14^]

Without having the ambition of exploring the chemistry of selenium in full, this review tries to summarize the evolution of the organoselenium chemistry pointing out the milestones, the successes and controversies of three different scientific stories in the areas of biology, catalysis and pharmacology, focusing on key‐details of our mechanistic understanding of organoselenides functioning. In doing so, an historical perspective on these topics is provided, starting from the very beginning of each field and highlighting the points of contact among the different communities. The discovery and understanding of glutathione peroxidase, the first mammalian selenoenzyme ever discovered will be reviewed first. Then, the field of organoselenium catalyzed oxygen‐transfers will be examined, in which low molecular weight organoselenium species mediate the decomposition of a somewhat inert oxidant such as hydrogen peroxide to allow the facile oxidation of organic substrates. Lastly, the biological mechanism of action of various organoselenides with known pharmacological activity will be described. These three topics become often intertwined, since glutathione peroxidase precisely catalyzes the reduction of hydrogen peroxide, as organoselenium catalysts do, and many pharmacologically active organoselenides were at least initially designed as glutathione peroxidase mimics. But remarkable differences emerge between the organic chemistry of selenium and selenium‐based proteins, and it is nowadays clear that the biological activity of organoselenides cannot be described by a simple glutathione peroxidase‐like mechanism.

## 2 The Road of Selenium into Biology: The Discovery and Mechanism of Glutathione Peroxidase

### 2.1 From Poisonous to Essential: The Two Faces of Selenium

Berzelius soon understood that selenium could be toxic, as he verified when investigating health problems at his sulfuric acid production plant. Particularly, Berzelius itself experienced firsthand the respiratory inflammation which resulted from breathing in gaseous selenium compounds.^[15]^ In fact, as he changed the ores used in the process, which contained only trace amounts of selenium, the health of the workers greatly improved. In the following century, the interest on selenium was very low and almost no further research was conducted on the element. The only notable use was in the first solar cells built in New York by Charles Fritts in the early 1880s.^[16]^ But it was in the late 1930s that selenium gathered the spotlight again, and for a second time it was because of its toxicity. It was recognized as an industrial hazard[^17^, ^18^] and as a cause of the insurgence of various cattle and poultry diseases.[^19^, ^20^, ^21^] Moreover, in addition to the already established toxicity of selenium, it was also observed that the element could have teratogenic effects in birds^[22]^ and possibly in human beings.[^23^, ^24^] Only in the mid‐50s, the group of Jane Pinsent discovered the first “benign” function of selenium.^[25]^ Studying the bacterium *Escherichia coli*, Pinsent found that trace amounts of selenite were necessary for the enzyme formic acid dehydrogenase to work properly. It was already 1957 when Schwarz and Patterson groups independently proved that selenium was essential also to animals.[^26^, ^27^]

Schwarz's group was working on identifying micronutrients that are essential to life. In their studies, they fed mice a diet consisting only of sugar and yeast. The observed effect was that most of the animals ended up dying of liver necrosis. This condition could be prevented if a supplement of methionine or vitamin E was added. Moreover, if a particular kind of yeast (American brewer's yeast) was used in the diet these pathological effects did not occur. Therefore, Schwarz concluded that there was another substance that could prevent the disease and called it “Factor 3”.^[28]^ An insightful suggestion by Dr. DeWitt Stetten,[^29^, ^30^] prompted Schwarz to check the content of selenium in a sample of Factor 3. Indeed, not only did the sample contain selenium, but it was also proven that it was that particular element that prevented liver necrosis in rats.^[31]^

The discovery of the essential role of selenium in biology contributed to an increased interest in the subject that resulted in the identification of some human diseases caused by selenium deficiency. The first one to be discovered was the so called Keshan disease, which is a cardiomyopathy.^[32]^ Sometime later also the Kaschin‐Beck disease (a disorder similar to rheumatoid arthritis) and myxoedematous cretinism were related to selenium deficiency, although that not being the only cause for the insurgence of these diseases. These findings confirmed that the element was essential also to humans. Moreover, since the discovery of the first selenium‐caused disease, research was conducted also to ascertain if selenium supplementation could be related to the incidence of cancer. From the very rich literature on the subject (for an exhaustive list of publications see^[11]^ and references therein), a quite homogeneous perspective emerged, which seemed to favor the conclusion that a low intake of selenium resulted in a higher cancer incidence. This so called “selenium‐cancer” hypothesis was further expanded by a large‐scale study by Clark and Combs which showed that 200 μg/day of selenium intake resulted in a significant reduction in colon, prostate and lung cancer.^[33]^ On the wave of this promising result, the US National Health Institute promoted another trial on an even larger scale.^[34]^ Unfortunately, the results of Clark and Combs were not confirmed in this study. In fact, no clear evidence that could relate selenium supplementation with cancer prevention was found.^[35]^ The different outcomes of the two major studies on the relationship between selenium and cancer did not contribute to establish a clear role of the element as a protective agent. In addition, research on some selenoproteins showed how their overexpression could help promote cancer growth.[^36^, ^37^, ^38^] These factors, combined with the narrow span between the amount of selenium needed by the organism (26 μg/day–35 μg/day^[39]^) and the threshold above which it becomes toxic (900 μg/day^[39]^), make selenium a multi‐faceted element which was aptly named “the essential poison”.^[40]^

### 2.2 Glutathione Peroxidase: The First Mammalian Selenoprotein

Among the various proteins that make use of selenium in the human body, the family of glutathione peroxidases (GPx) is one of the most studied and well characterized and one of their members was the first mammal selenoprotein to be discovered.^[41]^ The first evidence for the existence of a GPx is found in a paper of 1957 by Mills that states: “*Studies with hydrogen peroxide indicate that this enzyme catalyzes the oxidation of reduced glutathione by hydrogen peroxide, and thus may be termed a glutathione peroxidase*.” At that time not only was the presence of selenium in GPx still unknown, but a few years after Mills's paper, part of the scientific American community deemed the existence of GPx very unlikely.^[30]^ Nonetheless, in Europe, research on the subject continued, albeit in a modest way, and culminated in 1971 with the work of Flohé’s group. Starting from cow blood, Flohé managed to extract, purify, and characterize GPx.[^42^, ^43^] The first studies on the activity and mechanism of GPx as a peroxidase brought about by these new findings, although published in a review in German,^[43]^ reached the group of William Hoekstra in the US. They combined these discoveries with their work on the ability of selenium to prevent oxidative damage in selenium deficient rats^[44]^ and managed to discover a direct relationship between selenium and GPx activity.^[45]^ If this evidence proved unequivocally the need for selenium in the catalytic mechanism of GPx, it was still not clear if the element was present in the protein itself or if it acted as a diffusible co‐factor, since the selenium was reported to be present in sub‐stoichiometric amounts.^[46]^ The uncertainty was resolved by Flohé *et al*., who managed to precisely determine selenium in a highly purified bovine GPx sample. The enzyme was first subjected to neutron bombardment, and the ^75^Se was then quantified by means of a gamma spectrometer. The analysis undoubtedly confirmed the presence of four selenium corresponding to one gram atom per subunit of the tetrameric GPx1^[41]^ In the following years, this stoichiometry was confirmed also in sheep, rats, fish and humans[^47^, ^48^, ^49^, ^50^, ^51^] and was years later verified by sequencing bovine GPx1^[52]^ and GPx4.^[53]^ For more details of the early times of selenium biochemistry and its implications the reader is referred to a recent review of Flohé.^[54]^

These findings and the later discovery of the second mammalian selenoprotein,^[55]^ phospholipid hydroperoxide glutathione peroxidase (PHGPx, now GPx4) supported the belief that the catalytic power of selenium could substitute for heme in the catalytic decomposition of hydroperoxides, an assumption that had, however, to be refuted. When Maiorino *et al*. exchanged the catalytic selenocysteine of GPx4 against cysteine, the activity of this CysGPx4 enzyme was expectedly impaired.^[56]^ However, the bimolecular rate constant for the oxidation of the enzyme by phosphatidylcholine hydroperoxide was only decreased by less than 3 orders of magnitude and with 5⋅10^4^ M^−1^ s^−1^ was still orders of magnitude higher than any rate constant for the oxidation of any low molecular weight thiol by a hydroperoxide (Table 1). Moreover, naturally occurring CysGPxs, e. g. the GPx of *D. melanogaster*,^[57]^ displayed rate constants that were almost competitive with those of the mammalian selenoenzymes (for a review on the topic see^[58]^). Finally, after the discovery, in the laboratories of Bruce Ames and Earl Stadtman, of the second non‐heme peroxidase family,[^59^, ^60^] the peroxiredoxins, which only exceptionally work by selenium catalysis,^[61]^ it became clear that also sulfur can efficiently catalyze the reduction of hydroperoxides.

**Table 1:** Selected rate constants for chalcogen oxidation at nearly physiological pH.

Compound	Co‐reactant	k_+1_ (M^1^ s^1^) 1	Ref
Glutathione (GSH)	H_2_O_2_	0.9	^[62]^
Cysteine (Cys, C)	H_2_O_2_	2.9	^[62]^
Selenocysteine (Sec, U)	H_2_O_2_	35.4	^[63]^
Protein phosphatase PTP1B	H_2_O_2_	9 – 20	[^62^, ^64^]
Protein phosphatase Cdc25B	H_2_O_2_	~1.60⋅10^2^	^[62]^
Glyceraldehydephosphate dehydrogenase	H_2_O_2_	5⋅10^2^	^[62]^
Transcription factor OxyR	H_2_O_2_	~5⋅10^4^	^[65]^
Peroxiredoxins	H_2_O_2_	~10^4^–10^7^	[^62^, ^66^]
Transcription factor Ohr (Prx)	LoaOOH	3⋅10^7^	^[67]^
Cys‐glutathione peroxidases	H_2_O_2_	up to 1.6⋅10^6^	^[58]^
Glutathione peroxidase 1 (bovine)	H_2_O_2_	5⋅10^7^	^[68]^
Glutathione peroxidase 4 (porcine)	PCOOH	1.4⋅10^7^	^[58]^
Glutathione peroxidase 4 U→C	PCOOH	5⋅10^4^	^[58]^

PCOOH=Phosphatidylcholine hydroperoxide; LoaOOH=Linoleic acid hydroperoxide.

### 2.3 Selenocysteine: Legacy or Novelty?

**Figure 2:**
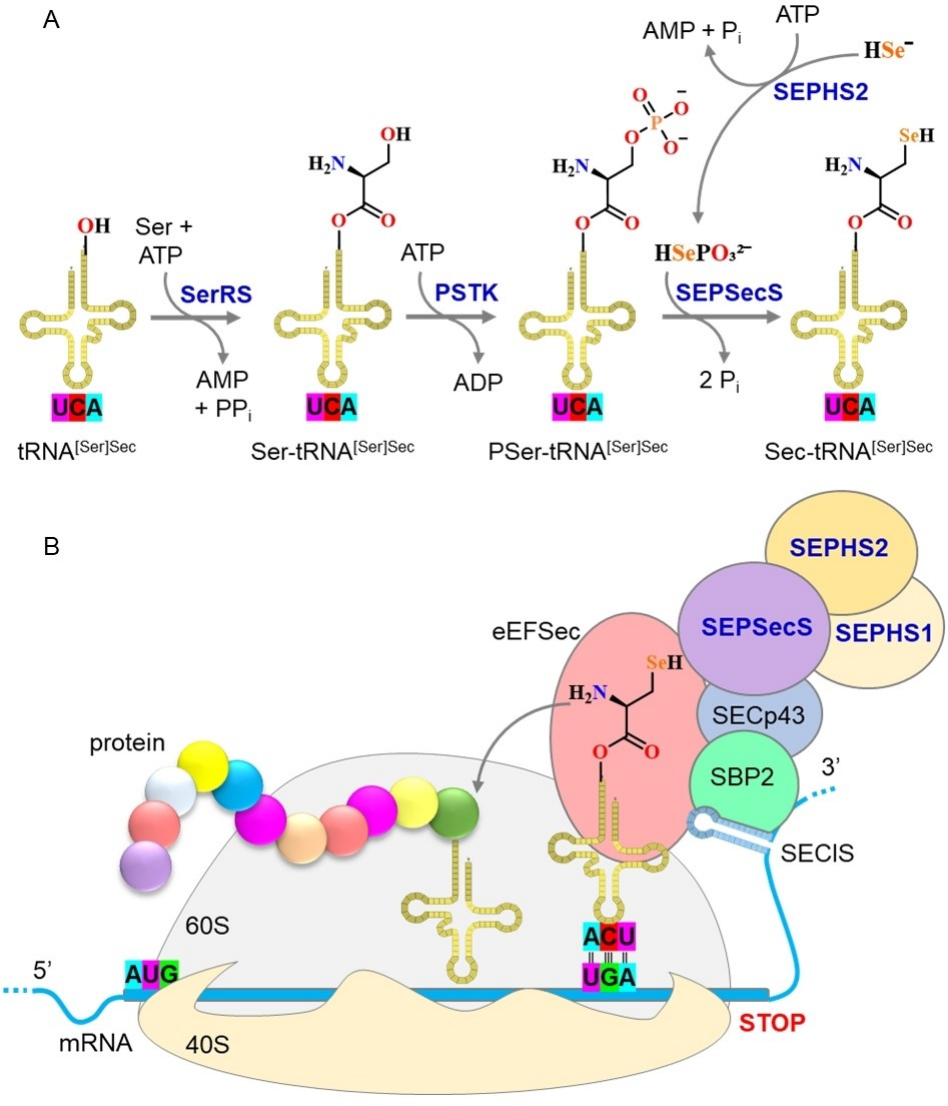
proposed mechanism of selenocysteine amino acid and selenocysteinyl‐proteins biosynthesis in eukaryotes. (A) Selenocysteine (Sec or U) is the 21^st^ amino acid utilized in protein synthesis. Its biosynthesis in eukaryotes initiates with the activation of selenide (Se^2−^ or HSe^−^) to produce selenophosphate (HSePO₃^2−^), a pivotal step catalyzed by the enzyme selenophosphate synthetase 2 (SPHS2). This activation serves as a regulatory point in the pathway. The backbone of Sec originates from serine (Ser). Initially, a serylation of Sec‐specific tRNA occurs, catalyzed by seryl‐tRNA synthetase (SerRS). The Ser bound to tRNA (Ser‐tRNA^[Ser]Sec^) undergoes phosphorylation by phosphoseryl‐tRNA kinase (PSTK, also known as L‐seryl‐tRNA(Sec) kinase), generating O‐phosphoseryl‐tRNA^[Ser]Sec^ (PSer‐tRNA^[Ser]Sec^). Subsequently, PSer‐tRNA^[Ser]Sec^ is converted into Sec‐tRNA^[Ser]Sec^, utilizing selenophosphate as a selenium source, by the enzyme O‐phosphoseryl‐tRNA selenium transferase (SEPSecS). (B) Sec biosynthesis and incorporation require a complex machinery with multiple protein‐protein/RNA interactions (eEFSec, SBP2, SECp43, SEPSECS, SEPHS1, SEPHS2, and Sec‐tRNA^[Ser]Sec^). The Sec‐tRNA^[Ser]Sec^ carries the Sec to the ribosome during translation. The incorporation of Sec into a protein requires a unique codon called the UGA codon, which normally functions as a stop codon. The Sec incorporation via the above molecular machinery is followed by the Sec insertion sequence (SECIS) in the untranslated region of the selenoprotein mRNA. The SECIS element guides the ribosome to read the UGA codon as Sec rather than as a stop signal. This process involves specific proteins such as SBP2 (selenocysteine insertion sequence binding protein 2), which interacts with the SECIS element, and other factors such as Sec‐specific elongation factor (eEFSec or SelB), which binds Sec‐tRNA^[Ser]Sec^ with high specificity and delivers it to the ribosome for selenoprotein production. The SECp43 (tRNA selenocysteine‐associated protein 1) stabilizes the SBP2, eEFSEC, and Sec‐tRNA^[Ser]Sec^ complex, enhancing the efficiency of selenoproteins synthesis, and may be involved in the methylation of Sec‐tRNA^[Ser]Sec^. Together, these components ensure the accurate translation of the UGA codon into Sec.[^74^, ^75^, ^76^]

The discovery of selenium in proteins was a great achievement. However, as with all scientific discoveries, it clarified many doubts but opened many more questions. First, how is selenium inserted into proteins? It took three years to solve this issue and the first conclusive evidence on the structure of a protein‐bound amino acid containing selenium was published only in 1976.^[69]^ It was unambiguously shown that the moiety containing the element is a cysteine residue in which sulfur is substituted by selenium (hence selenocysteine). Another ten years had to pass before it was eventually recognized that selenocysteine (Sec) could be rightfully called the 21^st^ amino acid.^[70]^ The major contributions that fueled the recognition of Sec as a proteinogenic amino acid were the discoveries that two genes, coding for two different selenoproteins, contained an in‐frame TGA termination codon.[^71^, ^72^] This led to the novel idea that the codon UGA, which normally is read as a stop codon, could, under “particular circumstances”, signal the insertion of Sec. These “particular circumstances” were seen to be a complicated machinery involving multiple co‐factors that are needed to differentiate the meaning of the UGA codon^[73]^ (Figure 2).

While all the steps building up the process of Sec insertion were being elucidated, the prevailing ideas about the presence of selenium in biology leaned towards the opinion that it was a legacy of the anaerobic world.[^77^, ^78^] The complexity and costliness of the reactions involved in the process clearly supported this view, which was corroborated by the higher selenium than sulfur sensitivity towards oxygen. Later on, studies on protein homology between prokaryotes and eukaryotes led to the abandonment of this theory in favor of a new point of view: selenium is recent evolutionary improvement and it has a unique and specific role and a particular advantage over sulfur resulting in a strong enhancement of the enzymatic reactions in which it is involved.^[79]^ This consideration seemed to be the natural result from all the studies revolving around the mutation of Sec to Cys in selenoenzymes or vice versa,[^56^, ^80^, ^81^, ^82^, ^83^, ^84^, ^85^] and stems also from the different chemical properties of selenium and sulfur based functional groups.[^86^, ^87^] For example, Sec is a better nucleophile but also a better electrophile than Cys, thus supporting an enhanced rate of the reactions in which it partakes. However there have been a few instances in which the catalytic role of selenium was difficult to justify.[^88^, ^89^]

Interestingly, terrestrial plants apparently lack selenoprotein catalysis.^[8]^ Such plants are steadily exposed to high oxygen pressure, temperature and light, i. e., to extremely stressing conditions. Nevertheless, not any single selenoprotein has ever been detected in any terrestrial plant, and the machinery to insert selenocysteine into proteins, which is typical for animals and lower organisms is also absent. Moreover, some insects have a reduced selenoproteome and even completely selenoproteinless animals have been identified.[^90^, ^91^] These findings strongly argue against a slow development of selenium catalysis in response to environmental stressors.

Despite these controversial views another possibility for selenium was proposed. If the presence of selenium was still viewed as an evolutionary sophistication, the main advantage of selenium over sulfur was ascribed not to its superior catalytic activity but to its more favorable redox properties.^[92]^ In other terms, due to its intrinsic atomic properties, selenium is better suited to efficiently sustain the high oxidative stress levels present in most of the environments in which selenoenzymes work, thus performing its catalytic role not only promoting fast reactions, but also thanks to the reversibility of organoselenium mediated processes.[^11^, ^78^, ^93^] This *redox advantage*, as previously labelled by Reich and Hondal,^[87]^ stems from intrinsic properties of the two chalcogens, i. e., from the well‐known preference for low oxidation states when increasing the atom size along a group in the periodic table, which prevents overoxidation of selenium to the highest (inactive) oxidation states, but also from enzyme specific circumstances (*vide infra* for the specific case of GPx4). Despite the limited number of reactions in which selenium is involved, which is a result of evolutionary pressure, some advantage over sulfur is expected, which, in theory, could replicate the physiological role of selenium. After more than 200 years since its discovery, the knowledge of the function of selenium inside the cell is still not exhaustive. Selenocysteine shows different chemical properties when compared to cysteine: lower pKa (5.2 vs 8.3),^[94]^ superior electron acceptor and leaving group ability,^[78]^ but these differences do not clearly explain why in some cases the choice of nature fell on selenium rather than sulfur.^[95]^ Moreover, in some complex eukaryotes it has been seen that the replacement of Sec with Cys causes no appreciable activity loss,^[89]^ suggesting that a deeper investigation on the chemical, biochemical and biological roles of selenium is still needed. Thus, far from being completely understood, the presence of selenium in biology with its complex insertion machinery relies on different hypotheses. Lately, the idea of a superior redox ability and a higher resistance to overoxidation seems to provide a convincing argument to disentangle this ongoing debate. Moreover, the recognition of the role of selenium in the redox regulation of cellular pathways such as signal cascades, transcription factor and apoptosis,[^96^, ^97^, ^98^] is another factor that could determine the essentiality of this element.

### 2.4 GPx Mechanism

Among the selenoproteins, some members of the glutathione peroxidase family play an important role in balancing hydroperoxide challenges. Hydroperoxides are toxic and necessitate elimination, but also serve as signaling molecules.[^99^, ^100^] Therefore, over‐optimizing hydroperoxide reduction can disrupt metabolic regulation. For instance, overexpression of glutathione peroxidase type 1 in mice led not to increased robustness but to obesity and insulin resistance.^[101]^

In glutathione peroxidases, H_2_O_2_ reduction commonly occurs *via* the oxidation of a selenocysteine residue. This reaction is typical in many members of the glutathione peroxidase (GPx) family[^102^, ^103^, ^104^, ^105^] and, in rare instances, in peroxiredoxins.^[61]^ These enzymes share a mechanism involving the oxidation of a selenocysteine residue to selenenic acid, followed by its reduction by a thiol. Although the formation of selenenic acid within the protein architecture has not been confirmed analytically, pioneering theoretical predictions and density functional theory (DFT) calculations support its feasibility.[^106^, ^107^]

Despite the lack of direct evidence, recent NMR studies by Goto and coworkers, have shown that a cradle‐protected selenocysteine can be oxidized to selenenic acid, which then reacts readily with thiols and more slowly with NH‐groups, while deselenylation is the least favored process.^[108]^

The reduction of oxidized selenocysteine residues is consistently achieved by a thiol group. Notably, the specificity for both the oxidizing hydroperoxide and the reducing thiol varies significantly among these enzymes. All glutathione peroxidases and almost all peroxiredoxins reduce H_2_O_2_ or other soluble hydroperoxides, whereas GPx4 reduces complex peroxidized lipids, even those integrated into biomembranes.^[105]^ This specialization of GPx4, particularly its cytosolic form, explains the role of this enzyme as a key‐regulator of ferroptosis.^[109]^ Many peroxiredoxins also react with complex peroxidized lipids,^[59]^ and some specifically reduce lipid hydroperoxides.^[110]^

The reductive part of the catalytic cycles is even more varied. GPx1, GPx2, GPx3, and GPx4 predominantly use glutathione (GSH) as their reducing thiol. However, GPx3 can also be reduced by thioredoxin, and GPx4 reacts with non‐physiological dithiols like dithiothreitol (DTT) and a range of physiological protein thiols, including its cysteine residues.[^105^, ^111^] The polymerization of mitochondrial GPx4 and its co‐polymerization with other cysteine‐rich proteins produce an enzymatically inactive structural protein aggregate essential for male fertility in mammals.^[102]^ GPx7, and likely GPx8, which are wild‐type CysGPx, prefer protein disulfide reductase as a reducing substrate, thereby playing a crucial role in oxidative protein folding. Most cysteine‐containing GPx homologs in bacteria, protozoa, plants, and insects use thioredoxin or related redoxins as reductants,^[105]^ as do the 2‐Cys peroxiredoxins.

**Figure 3:**
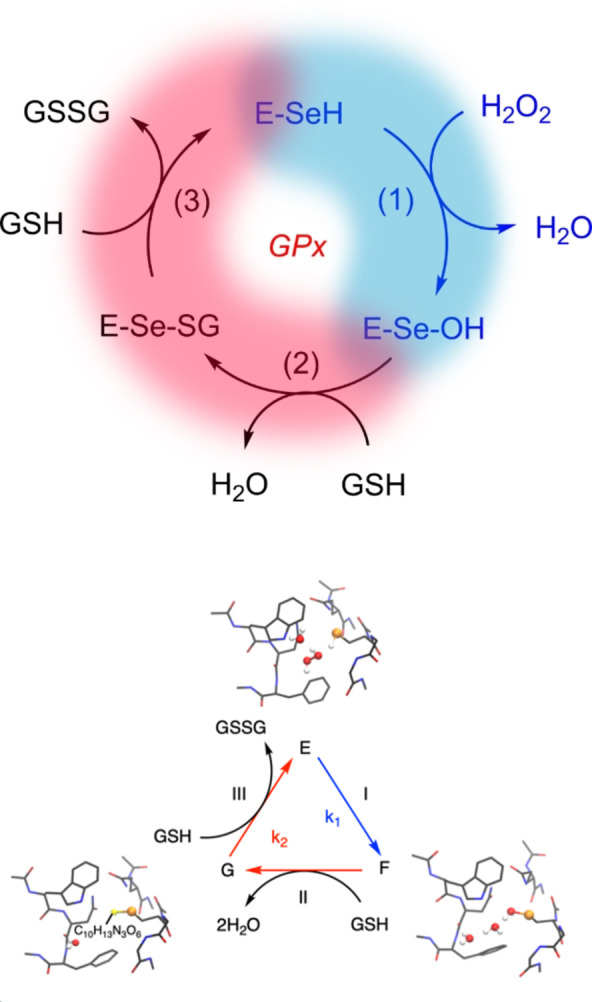
Mechanism of hydroperoxides reduction catalyzed by GPx. (**a**) Catalytic mechanism of SecGPx. GSH=Glutathione, GSSG=glutathione disulfide. The peroxidatic part of the catalytic cycle is highlighted in blue and the reductive part in red. (**b**) Close‐up on the intermediate identified within a representative catalytic pocket as obtained by DFT calculations. E is the reduced enzyme in with the Cys/Sec in the thiol/selenol form (E‐XH), F represents the oxidized intermediate, the sulfenic/selenenic acid (E‐XOH) and G is the disulfide/selenosulfide species (E‐XSG), X=S, Se.

The molecular mechanism of glutathione peroxidase has been thoroughly investigated in the past decade and this biochemistry has been tightly connected to peculiarities of selenium chemistry disclosed *in silico* and recently supported by the experimental peptide models of Goto and co‐workers.[^108^, ^112^] As described above, GPx enzymes catalyze the reduction of hydrogen peroxides and organic hydroperoxides by glutathione (GSH).^[113]^ First, the oxidation of selenol (E) to selenenic acid (F) occurs (Figure 3), accompanied by the reduction of the hydroperoxide; thereafter, two equivalents of GSH are consumed in the two subsequent reductive steps: first a selenenylsulfide intermediate (G) is formed and then the reduced enzyme is regenerated.[^55^, ^114^] The kinetics of this mechanism were seen to be more complicated than a Michaelis‐Menten process in which an overall kinetic constant can be defined.^[115]^ The kinetic constants for the reductive part are usually 2–3 orders of magnitude smaller than that of the oxidative step (k_1_). However, the much higher concentration of GSH *in vivo* compared to peroxide (mM vs nM‐μM) usually makes the overall reaction independent of the concentration of the reducing agent.

**Figure 4:**
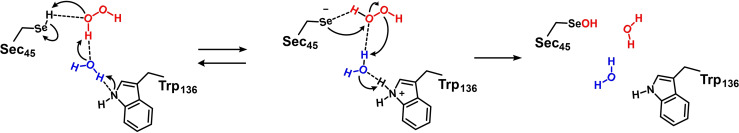
Stepwise peroxide bond breaking as it is predicted to occur in GPx4. Residue numeration is referred to human GPx4.

Numerous attempts have been made to explain the unique kinetics and efficiency of glutathione peroxidases. In 2015, the first satisfactory explanation based on DFT calculations was provided.^[103]^ These calculations revealed that, within a model of six conserved amino acid residues constituting the active site of nearly all glutathione peroxidases, the selenocysteine residue dissociates, a finding consistent with its low pK value (5.2). Surprisingly, a cysteine residue in this position also dissociates in this environment despite its higher pK (>8). The proton released from the Sec/Cys remains bound in the active site, migrating through a network of hydrogen bonds formed by water molecules (and eventually hydrogen peroxide itself) to the imidazole nitrogen of a conserved tryptophan residue, leading to a charge–separated complex. Upon the addition of H_2_O_2_, this complex decays without measurable activation energy, with the selenium (or sulfur) nucleophilically attacking one oxygen of the peroxide bond while the dislocated proton, mediated by water, electrophilically attacks the second oxygen. This dual attack cleaves the peroxide bond, forming selenenic (or sulfenic) acid and water.[^103^, ^116^] This mechanism accounts for the unusual kinetic behavior of glutathione peroxidases, where the enzyme/H_2_O_2_ complex does not accumulate due to its rapid reaction rate, consistent with the lack of saturation kinetics (infinite Michaelis constants and maximum velocities) and the high rate constant for the enzyme's reaction with H_2_O_2_ (ca 10^8^ M^−1^ s^−1^ for bovine GPx1).^[117]^ It is important to stress that other residues in the catalytic pocket besides tryptophan may aid the proton dislocation, as long as the acceptor is in proximity of the substrate (Figure 4).^[103]^

As already mentioned, the selenocysteine in glutathione peroxidases (GPxs) can be replaced by cysteine without altering the basic H_2_O_2_ reduction mechanism. However, when cysteine substitutes selenocysteine via site‐directed mutagenesis, there is a significant decrease in specific activity and rate constants for both the oxidative and reductive parts of the catalytic cycle. Naturally, this substitution occurs in human GPx5, GPx7, GPx8, and in most non‐vertebrate GPx proteins. Despite lacking selenocysteine, these “CysGPxs” can exhibit high reaction rates with hydroperoxides (up to 10^6^ M^−1^ s^−1^), though generally less efficient than their selenium‐containing counterparts. A recent DFT study also corroborated that selenium‐based enzymes are more reactive in hydrogen peroxide reduction.^[118]^

A key qualitative difference in GPx catalysis arises in the absence of a reducing substrate. Natural selenoproteins (GPx1 or GPx4) form reversible Se−N bonds with the protein backbone, while cysteine homologs overoxidize to sulfinic and sulfonic acids.^[103]^ This reflects selenium's tendency to remain in lower oxidation states than sulfur,[^11^, ^119^] and provides a self‐protection mechanism against irreversible enzyme destruction. Also this bypass mechanism has been experimentally observed in Goto's protected peptide models.[^108^, ^112^] Importantly, a potentiation of peroxide‐induced cell death (ferroptosis) was seen when Sec was substituted by Cys in GPx4.^[120]^ The superior resistance of SecGPx to overoxidation would let it work as a signaling agent through peroxide regulation without compromising the cell viability in peroxide‐rich environments.

The dual attack principle, initially identified in GPxs, has been extended *in silico* also to peroxiredoxins.^[121]^ Here, the proton of the peroxidatic cysteine migrates to a conserved threonine residue, which can only be replaced by serine without loss of activity.^[122]^ This mechanism was identified to be active, *in silico*, also in non‐peroxidase proteins like Glyceraldehyde 3‐phosphate dehydrogenase GAPDH and the bacterial hydroperoxide sensor OxyR that can react with H_2_O_2_ much faster than low molecular mass thiols. Investigating other protein families with super‐reactive cysteine residues for this mechanism appears promising.^[121]^

### 2.5 Peroxynitrite Reductase Activity of Glutathione Peroxidase

While the catalytic mechanism of GPxs with H_2_O_2_ is well understood, the way in which these enzymes deal with peroxynitrite (ONOO^−^) and its conjugate peroxynitrous acid (HOONO) remains elusive. Peroxynitrite is a strong oxidant and nitrant which is formed under biological conditions from the reaction of the nitroxide radical (NO⋅) with the superoxide anion (O_2_⋅^−^).[^123^, ^124^] The reaction between O_2_⋅^−^ and NO⋅ is so fast that it occurs even in the presence of superoxide dismutase.[^123^, ^125^] Even if the product of the reaction is the peroxynitrite anion, ONOO^−^, its pKa of 6.8 implies that, at biologically accessible pH values, this oxidant is present as a mixture of peroxynitrite and peroxynitrous acid, with the predominant form depending on the local pH. Under most biological conditions, both species will be present in some percentage.^[125]^

Both oxidation^[126]^ and nitration[^127^, ^128^] promoted by peroxynitrite can lead to cytotoxic effects, due to its capacity to modify biomolecules such as lipids, amino and nucleic acids.[^123^, ^129^, ^130^, ^131^, ^132^] Importantly, before the 90s, no enzymatic protection against peroxynitrite damage was recognized.^[133]^ Only after the discovery that the GPx‐mimic ebselen (*vide infra*) can act as a peroxynitrite scavenger,[^134^, ^135^, ^136^] the same “peroxynitrite reductase” activity was proposed for GPx.^[137]^ Particularly, Sies and coworkers observed that both GPx1^[137]^ and selenoprotein P can be implicated in the defense against peroxynitrite.^[138]^ However, in the same period, it was also observed that GPx is inhibited by the presence of peroxynitrite in the absence of glutathione.[^139^, ^140^, ^141^] These results suggest that, in the reduction of peroxynitrite, a different oxidized enzymatic intermediate might be formed, i. e., different from the canonical selenenic acid (E‐SeOH)^[139]^ (Figure 4), or the protective selenyl amide formed by intramolecular condensation of selenenic acid when the enzyme deals with other hydroperoxides.^[103]^ Indeed, these two species can be reintegrated in the canonical catalytic mechanism in the presence of glutathione, thus not explaining GPx inactivation.^[139]^ Additionally, at the beginning of the 2000s, Fu and coworkers^[142]^ observed that neither GPx1 knockout hepatocyte nor wild type cells were sensible to peroxynitrite induced apoptosis. They proposed that GPx1 is not required by hepatocytes to cope with peroxynitrite. In the same year, Sies and coworkers^[143]^ observed that, even more strikingly, GPx1 knockout hepatocyte can be more resistant against peroxynitrite induced damage than wild type cells, suggesting an apoptosis inducing role of GPx in the presence of peroxynitrite. To the best of our knowledge, a complete understanding of the topic is still to be reached, and this behavior of GPx1 (as well as similar behaviors of other antioxidant enzymes) has been considered paradoxical.^[144]^

The potential energy surface (PES) of the catalytic mechanism of GPx as a peroxynitrite reductase has been previously investigated *in silico* by Morokuma and coworkers, employing a simplified cluster encompassing three out of the four essential amino acids which compose the enzyme catalytic tetrad.[^145^, ^146^, ^147^] In their investigation, the PES was studied by comparing two possible alternative mechanisms, i. e., one in which the peroxynitrite oxidizes GPx Sec to the canonical selenenic acid, and a second one in which the peroxynitrite acts as a nitrating agent, leading to a nitrated Sec intermediate.^[146]^ However, in their study, the oxidation to selenenic acid was found to be preferred, thus leaving the problem of a plausible inactive intermediate open.

While the reaction of thiols with peroxynitrite has been variously explored in the past years, far less investigation has been conducted with selenols.^[133]^ Particularly, peroxynitrite is known to be able to oxidize thiols to disulfide,[^133^, ^148^] with sulfenic acid as a plausible intermediate also in proteins,[^133^, ^148^, ^149^] in analogy to thiol chemistry with hydroperoxides. Importantly, peroxynitrous acid is relatively unstable in biological conditions and in the absence of reducing agents it undergoes a spontaneous degradation.^[150]^ However, its reaction with thiols to produce sulfenic acid is faster than this degradation mechanism.[^151^, ^152^] Additionally, peroxynitrite is known to be capable of nitrating thiols, leading to nitrothiols such as nitroglutathione (GS‐NO_2_),^[153]^ as well as capable of nitrosylating thiols, leading to nitrosothiols such as nitrosoglutathione (GS‐NO).^[154]^ The formation of radical species seems to be excluded in the nitrosylation reaction[^139^, ^154^] and a direct nucleophilic substitution mechanism has been proposed. To the best of our knowledge, no nitrosylation of Sec by peroxynitrite has ever been reported, and only very recently the nitrososelenocysteine (Sec‐SeNO) species was isolated in a molecular cradle by Goto and coworkers,^[155]^ thus enabling the possibility to study its chemistry.

### 2.6 Other Selenoproteins

**Figure 5:**
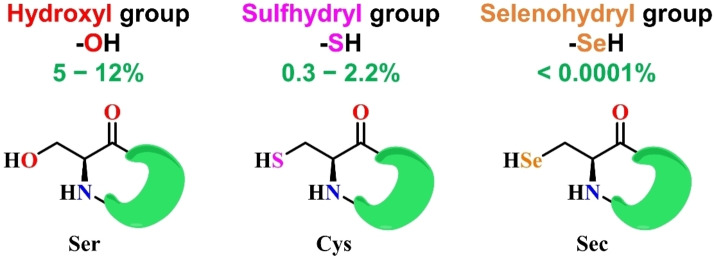
Relative (%) occurrence of serine, cysteine and selenocysteine residues in proteins.^[156]^

Selenium is a quite rare element in biology. The relative and roughly estimated abundance of serine, cysteine, and selenocysteine residues in proteins from organisms of different kingdoms is schematically represented in Figure 5 and this aspect further motivates the curiosity of its biological function.^[8]^ GPx1 and GPx4 were the first selenoproteins ever discovered. In the previous discussion, we described in detail their catalytic principle, which is the best known biological organoselenium mechanism currently understood.

**Figure 6:**
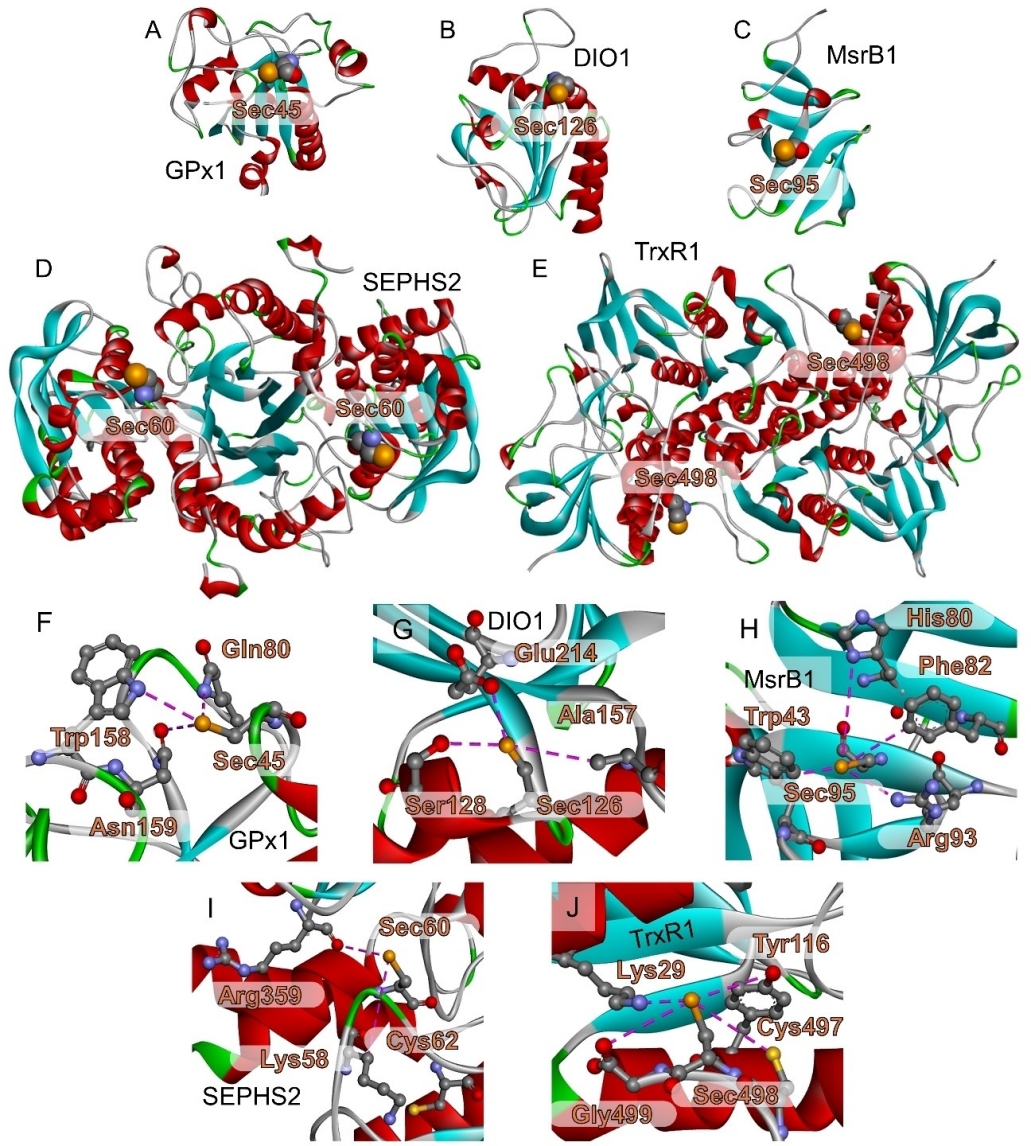
Tridimensional structures (3D) of selenoenzymes. (A) GPx1 [1GP1], (B) DIO1 [P49895/4TR3], (C) MsrB1 [3MAO], (D) SPHS2 [Q99611/3FD5], (E) TrxR1 [3 J2 N], and close up on the residues in the immediate surrounding of the Sec residue in each protein (F–J). The source of the 3D structures (PDB or protein homology modeling from Swiss‐Model) is indicated in the brackets.

However, at the beginning of the 2000s, thanks to the efforts of Gladyshev and coworkers,[^157^, ^158^] 25 genes were identified in humans coding for selenoproteins.^[159]^ As of today, only six protein classes have clearly defined functions in mammals, i. e., glutathione peroxidase (GPx), methionine sulfoxide reductase (Msr), thioredoxin reductase (TrxR), iodothyronine deiodinase (DIO), selenophosphate synthetase (SPHS2) and the Se transporter selenoprotein P (Figure 6).^[160]^ The precise discussion of all mammalian's selenoproteins is out of the scope of this review, and the interested reader can consult classical reviews on the matter.[^8^, ^95^, ^161^, ^162^, ^163^]

Many of them are directly or indirectly involved in the defense against oxidative damage (GPx, TrxR and MsrB1). However, it would be rather strange to list the deiodinases (DIO), which form or degrade the thyroid hormone thyroxine, under the antioxidant enzymes.[^160^, ^164^] It therefore seems advisable to look for functions distinct from “antioxidant activities” of selenoproteins with still unknown biological role. Certainly, selenium is known for its extraordinary catalytic redox activity, but the final outcome of such redox catalysis might be completely unrelated to the prevention of oxidative damage. To this end, organic chemistry may provide numerous examples in which an organoselenium catalyst employs hydrogen peroxide as sacrificial oxidant to introduce or modify functional groups in organic molecules. (see below).

## 3 Selenium in Organic Chemistry: Organoselenium‐Catalyzed Oxygen‐Transfer Reactions and Hydrogen Peroxide Activation

It is perhaps by chance that the same years that saw the birth of selenium enzymology also marked the beginning of the widespread use of Se in organic chemistry. Indeed, before the 70s, the role of Se in organic synthesis was mainly limited to the use of the inorganic SeO_2_ as a (sometimes catalytic) oxidant.[^3^, ^165^, ^166^, ^167^] During such oxidations, variable quantities of organoselenides were known to form but at the time were considered unwanted side products and received little attention until the end of the 60s.^[166]^ This does not mean that the *organic chemistry of selenium* was undeveloped. Quite the contrary, the birthday of the field can be traced back at least to 1847, when, according to Fredga, Wöhler wrote to Berzelius: “*a small grandchild of yours has come into the world, a child of selenium, the selenomercaptan*”.^[168]^ The first full publication on *selenomercaptan* was published in the same year, mentioning possible early contributions to organoselenides synthesis by Lowig.^[169]^ Indeed, by the first half of the 1900, many classes of organoselenides had been synthesized, even if the references were scattered and the field was characterized by lack of systematicity.[^170^, ^171^, ^172^] In fact, even after the discovery of the vital role of Se in living beings, organic chemists of the time were affected by a form of *selenophobia*,[^6^, ^173^] not only due to the well‐known toxicity of selenium, but also to the *infernal* smell which was historically associated to organoselenium work.[^168^, ^173^] An instructive anecdote is provided in a 1972 communication by Arne Fredga,^[168]^ where the unfortunate attempt of an organic chemist to synthesize some organoselenides at the beginning of the 20th century, is remembered:

*“He soon found that work indoors was impossible and pursued the experiments on the roof of the building, but the smell spread over defenseless Cambridge. It caused much commotion and partly spoiled the centenary celebrations of Charles Darwin's birth. The origin of the smell was soon discovered, and he had to assemble his equipment in an open field in the fens, far from human dwellings. Laboratory work in this place was of course not comfortable, and in addition he was pestered by herds of creeping and flying insects who found the smell attractive. At last, he resigned, and the project was abandoned.”*

What was overall missing, before the 70s, was any practical exploitation of *the reactivity of organoselenides*.^[174]^ Huguet in 1967 was probably one of the first to focus on the organoselenides formed during the oxidation of alkenes with SeO_2_, *inter alia* postulating for the first time a reaction step which would have been known in the future as selenoxide elimination. (*vide infra*)^[166]^ Still, organoselenides were regarded at best as reactive intermediates in reactions mediated by an inorganic selenium oxidant.[^166^, ^175^]

**Scheme 1:**
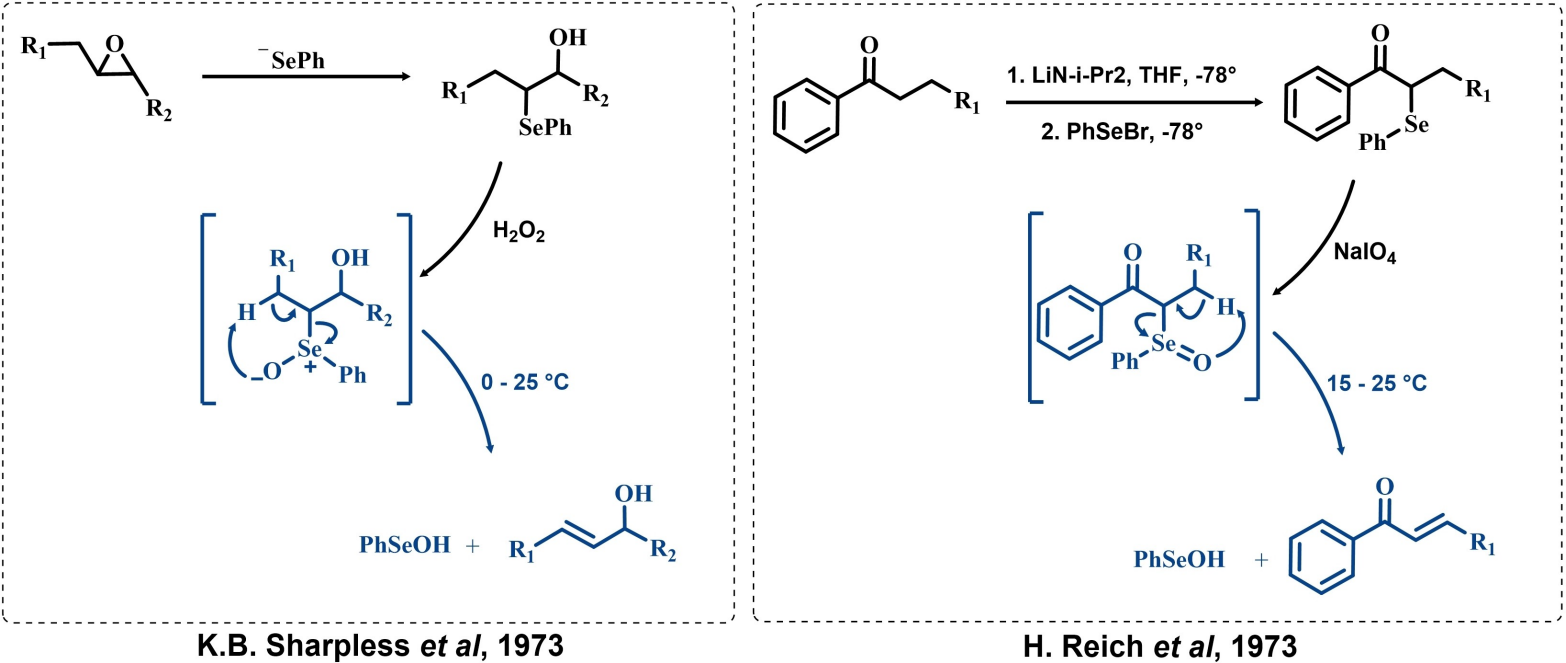
First investigations and applications of the selenoxide elimination appeared in the literature by the groups of Sharpless and Reich for the synthesis of allylic alcohols and α,β unsaturated carbonyl compounds, respectively.[^176^, ^177^] The selenoxide elimination step is highlighted in blue. Differences in representation of the selenoxide bond (i. e., as a charge‐separated Se−O single bond, or as a true Se=O double bond) are faithful to the choices of Sharpless and Reich, respectively.

Things changed with the recognition that organic selenoxides, i. e., organic species with a formal selenium=oxygen double bond, could rapidly undergo an intramolecular deselenylation reaction (the so‐called *selenoxide elimination*) which leads to formation of alkenes at room temperature, under very mild conditions, thus providing an easily accessible route to olefine synthesis (Scheme 1). The thermal instability of selenoxides had been known for decades, but the decomposition pathway was somewhat uncharacterized and thus its synthetic potential remained unappreciated.[^170^, ^174^]

After the first definitive observation at the very beginning of the 70s,[^178^, ^179^] the groups of Sharpless,[^177^, ^180^, ^181^] Reich^[176]^ and Clive^[182]^ pioneered in popularizing the reaction by highlighting its synthetic usefulness (Scheme 1). This reactivity has been widely exploited in the following years by various groups who confirmed the synthetic potential of the reaction by employing the selenoxide elimination in various synthetic protocols.[^3^, ^183^, ^184^, ^185^] Even if other reactions of organoselenides were developed and characterized at the beginning of the 70s,[^186^, ^187^, ^188^, ^189^, ^190^, ^191^, ^192^, ^193^] the discovery of the selenoxide elimination is currently recognized as the breakthrough responsible for the development of organoselenium chemistry applications into conventional organic synthesis.[^3^, ^6^, ^194^, ^195^]

An important step forward in the development of modern organoselenium chemistry, was the awareness that like the inorganic SeO_2_, also organoselenides could be employed for the oxidation of organic substrates. Particularly, the oxidizing power of seleninic anhydride was observed by the group of Barton[^196^, ^197^, ^198^, ^199^] and then further explored by Back and coworkers,[^200^, ^201^] expanding the scope of organoselenides with applications in organic synthesis. While these first rudiments of organoselenides reactivity required a stoichiometric amount of selenium, even before the end of the 70s the first organoselenium catalysts started to appear in the literature.

**Scheme 2:**
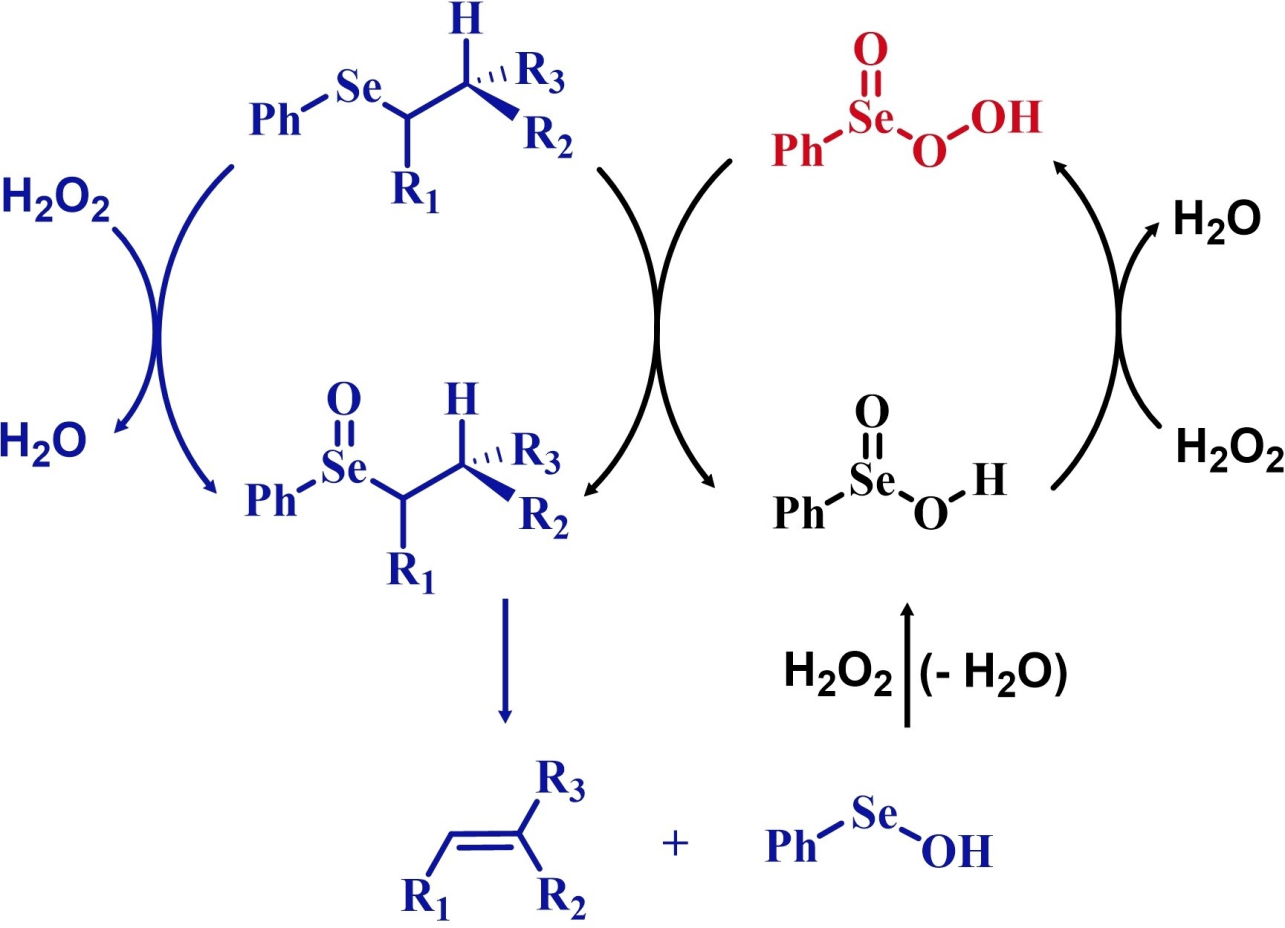
Mechanistic hypothesis to rationalize the autocatalytic oxidation of β‐eliminating organoselenides by Reich *et al*. The inductive step (i. e., prior to autocatalysis) is highlighted in blue, the peroxyseleninic acid is highlighted in red.

Indeed, the first observation of the catalytic potential of organoselenides in oxygen‐transfer reactions can be traced back to the pioneering work of Reich and coworkers in 1975. While working on the selenoxide elimination, they reported that selenides with β‐hydrogens (a prerequisite for the elimination) were oxidized by H_2_O_2_ faster than selenides lacking such feature.^[202]^ A plausible explanation was provided in terms of further oxidation of the released phenyl selenenic acid to phenyl seleninic acid,^[177]^ which was hypothesized to lastly “activate” H_2_O_2_ in the form of a peroxyseleninic acid. This species was deemed capable of oxidizing the remaining selenide to selenoxide faster than H_2_O_2_ itself, thus triggering an autocatalytic reaction^[203]^ (Scheme 2).

These observations reported the first example in a long series of oxygen‐transfer reactions mediated by organoselenium species and usually rationalized on the basis of the catalytic cycle in the right of Scheme 2. In the following, the historical development of the field in the past fifty years will be covered, highlighting successes in the employment of the organoselenides as oxygen‐transfer catalysts, as well as current controversies and areas where there is still room for improvement.

### 3.1 Organoselenium Catalyzed Oxygen‐Transfer Reactions Involving C=C and C=O Bonds

**Scheme 3:**
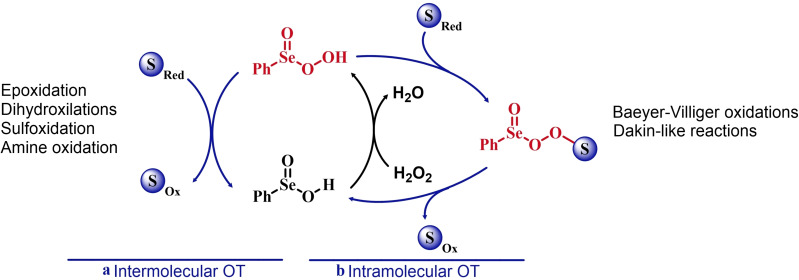
Generic cycles for substrate (S) oxidation via seleninic−peroxyseleninic acid catalysis. **a**. concerted substrate oxidation via intermolecular S_N_2‐like oxygen‐transfer; **b**. stepwise mechanism with formation of a covalently bound intermediate involving the organoselenium catalyst and the substrate.

Two possible mechanistic variations of the same scheme have been hypothesized to describe organoselenium catalyzed oxygen‐transfer reactions with hydrogen peroxide (Scheme 3). Either hydrogen peroxide is activated in the form of a peroxidic species which then can transfer the oxygen atom to an organic substrate in an intermolecular S_N_2‐like fashion or via the intermediate formation of a covalent adduct between the organoselenium peroxyacid and the organic substrate, with sequential intramolecular oxygen‐transfer reactions. For the catalytic couple phenyl seleninic–peroxyseleninic acid, these two pathways are represented in Scheme 3a and b, respectively.

Not all reactions fall properly within these two categories, and for some oxygen‐transfers the mechanism is still uncertain and might proceed via either or both pathways. Additionally, organoselenides can catalyze glutathione peroxidase‐like oxygen‐transfer reactions, in which a thiol is converted into a disulfide via different mechanisms, depending on the nature of the organoselenide catalysts (*vide infra*).

We will describe firstly oxygen‐transfer reactions that follow the mechanistic course sketched in Scheme 3. Then, some fundamentals of glutathione peroxidase mimics chemistry will be provided and then extended into their biological applications (paragraph 4).

#### 3.1.1 Epoxidations and Dihydroxylations of Olefines

**Scheme 4:**
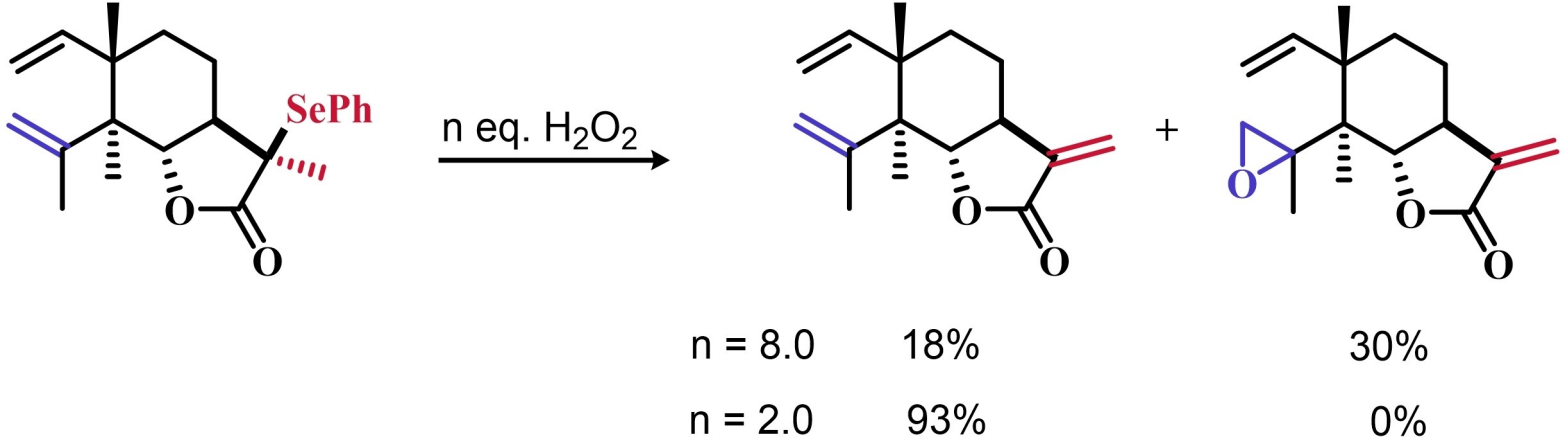
Pioneering organoselenium‐mediated epoxidation of alkenes. The effect of the addition of different equivalents of hydrogen peroxide is highlighted.

The organoselenium mediated epoxidation of olefines was reported as early as 1977 by Grieco and coworkers,^[204]^ and it was a serendipitous discovery. Indeed, Grieco *et al*. were investigating applications of the selenoxide elimination into the synthesis of α‐methylene lactones^[205]^ (Scheme 4), when they observed partial epoxidation of the unsaturated substrate produced via the selenium‐mediated olefination. Particularly, the epoxide was observed to be the major product when a large excess of hydrogen peroxide was employed (8.0 equivalents) to induce the selenoxide elimination. Conversely, no epoxide was observed when only a moderate excess of hydrogen peroxide was used (2.0 equivalents).

As previously hypothesized by Reich and coworkers, the further oxidation of the substrate was rationalized considering the overoxidation of the selenenic acid released by the selenoxides elimination to seleninic acid which lastly activates hydrogen peroxide in the form of a peroxyacid (Scheme 2). To the best of our knowledge, this might also be the first time that the term “benzene peroxyseleninic acid” appeared in the literature, even if peresters of organic seleninic acids had been previously proposed as the result of the reaction of diselenides with organic peroxides such as tert‐butyl hydroperoxide.^[206]^

**Scheme 5:**
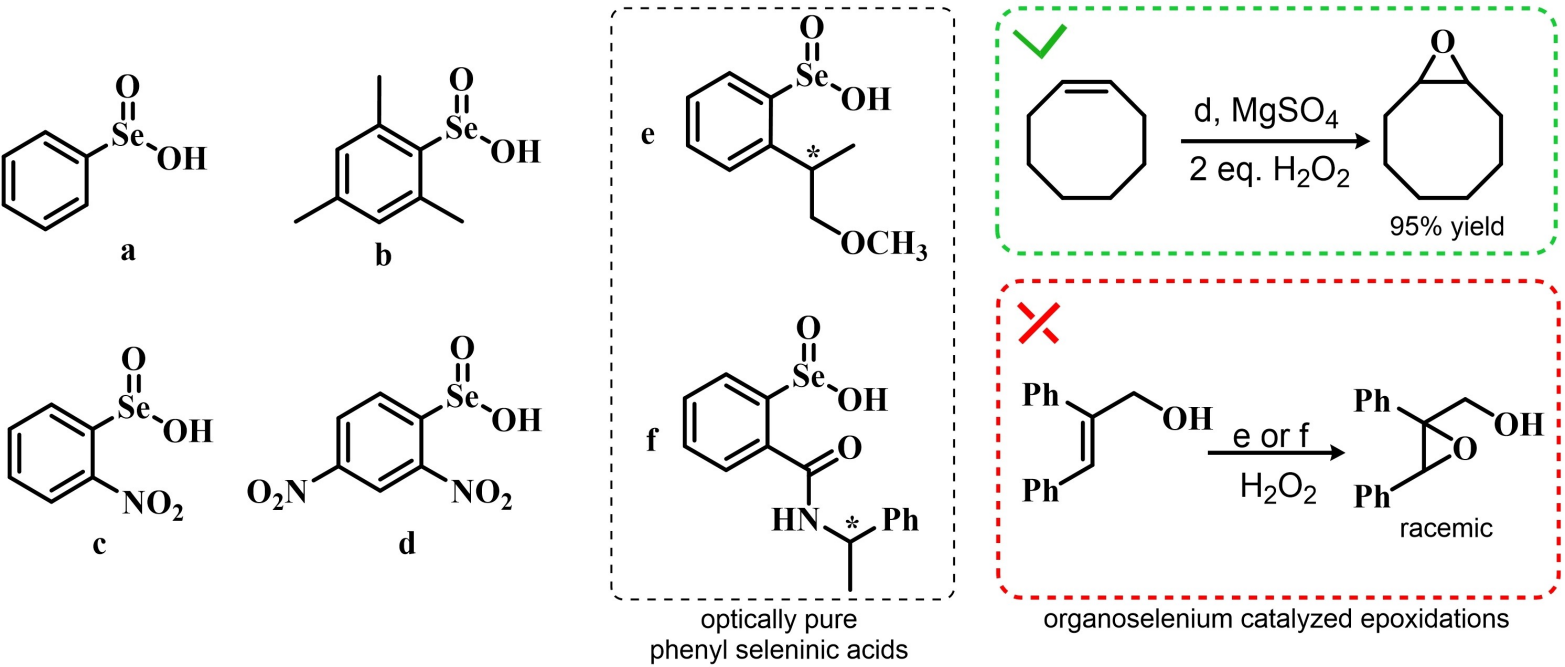
Selected active phenyl seleninic acids tested by Sharpless and Hori for epoxidation of alkenes; optically pure phenyl seleninic acids; successful preparative epoxidation of cycloctene and unsuccessful asymmetric epoxidation of allyl alcohols. The catalysts **a–d** were employed in the reaction mixtures as the corresponding diselenides and activated *in situ* with hydrogen peroxide. The green check indicates a successful application, while the red cross an unsuccessful application.

**Scheme 6:**
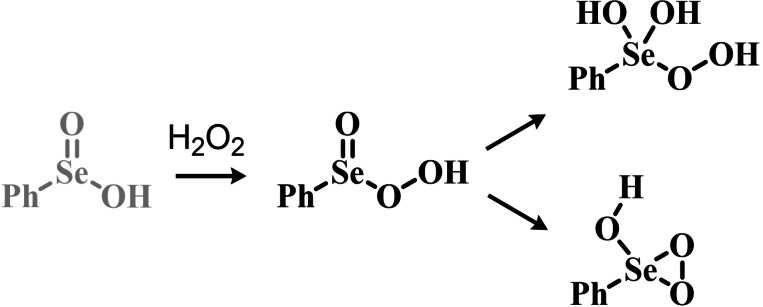
Sharpless’ and Hori's hypothesis about the nature of the epoxidizing agent to discuss the lack of stereoselectivity displayed by optically pure seleninic acids **e** and **f**. The precursor seleninic acids is represented in grey.

Building on these preliminary results, Grieco and coworkers explored the stoichiometric use of benzene peroxyseleninic acid, produced *in situ* by the reaction of benzene seleninic acid with hydrogen peroxide, for the effective epoxidation of a plethora of alkenes at room temperature using only a slight excess of peroxide.^[204]^ The catalytic version of the same reaction was reported less than one year later by Hori and Sharpless, who, beside confirming the results of Grieco *et al*., also explored some substituted phenylseleninic acids as epoxidation catalysts (Scheme 5). Notably, the preparative scale epoxidation of cyclooctene with 5 mol % of 2,4‐dinitrophenyl seleninic acid **d** and hydrogen peroxide as final oxidant was successfully reported. Sharpless and Hori also attempted the use of chiral, optically pure phenylseleninic acids **e** and **f** in combination with hydrogen peroxide as a route to asymmetric epoxidation of pro‐chiral alkenes. While both compounds were active epoxidation catalysts, the product epoxide was found to be racemic (Scheme 5). Hypothesis regarding the lack of asymmetric induction in the alkene epoxidations revolved around the plausible formation of a hydrate or cyclic peroxidic species (Scheme 6), with consequent loss of the stereogenic selenium center. Additionally, it was speculated that the large distance between the stereogenic carbon and the selenium nucleus might hamper the asymmetry induction in the formation of the peroxyacid.^[207]^

In the same years, the catalytic power of phenyl seleninic acids as epoxidizing agents was also independently observed by Reich, whose group additionally reported the effective epoxidation of tetrasubstituted olefines with benzene seleninic acid and the mono or dinitro derivative **c** and **d**. In these cases, 1 mol % of catalyst was sufficient to obtain good yield in epoxide (>90 %) at room temperature, in 2–5 hours and with a moderate excess of hydrogen peroxide (1.5–2 eq.)^[202]^

**Scheme 7:**
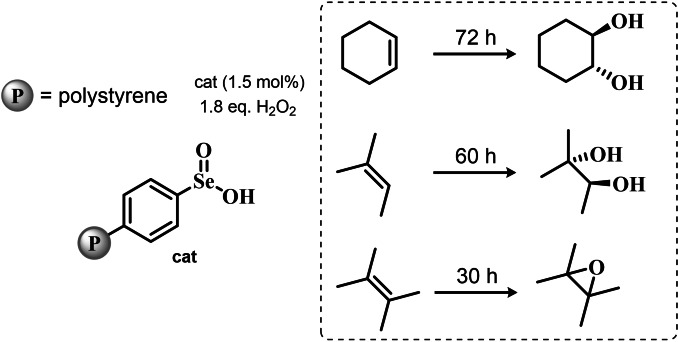
Polystyrene‐bound phenyl seleninic acid catalyst (cat) for the dihydroxylation of alkenes.

Despite these promising results, no further result about organoselenium catalyzed epoxidations was published until 1983, when a heterogenized phenyl seleninic acid bound to a polystyrene polymer was proposed as an oxygen‐transfer catalyst against a variety of substrates, among which substituted alkenes.^[208]^ Depending on the substrate, either the 1,2‐diol or the epoxide was recovered as the oxidized product. Particularly, the fully substituted tetramethyl ethylene was neatly oxidized to the corresponding epoxide. Conversely, in the oxidation of di‐ and tri‐substituted olefines only the corresponding diol could be observed (Scheme 7).

The observation of epoxide formation for tetrasubstituted olefines and especially the oxidation of cyclohexene to trans diols exclusively led the authors to hypothesize that the phenyl seleninic acid mediated di‐hydroxilation proceeded through a selenium‐catalyzed epoxidation followed by nucleophilic ring opening.^[208]^ To the best of our knowledge, this is up to today the commonly accepted mechanism for all reported organoselenium‐mediated dihydroxilations of alkenes (*vide infra*).[^4^, ^209^]

**Scheme 8:**
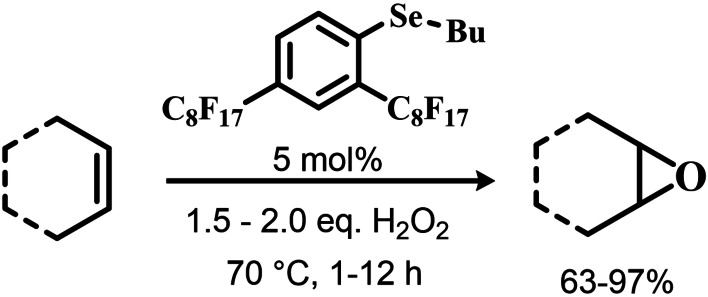
Organoselenium catalyzed epoxidation of alkenes in perfluorinated solvents.

Further investigation by Syper and Mlochowsky in the late 1980s and 1990s also highlighted the potential of phenyl peroxyseleninic acids in epoxidations, when both the phenyl seleninic acids or the corresponding precursors diselenides were employed as catalysts.[^210^, ^211^] Additionally, Betzemeier and coworkers reported the use of a phenyl alkyl selenide as effective catalyst for epoxidation in perfluorinated solvents^[212]^ (Scheme 8).

For some systems, dihydroxylation also occurred. With respect to other previously reported organoselenium catalysts, the catalysts proposed by Betzemeier and coworkers required temperatures around 70 °C to make the reaction feasible,^[212]^ while Reich and Sharpless pioneering catalysts were all active at room temperature. Based on previous literature, an activation of this monoselenide by hydrogen peroxide to the seleninic acid form was hypothesized, in analogy with Scheme 2.

At the beginning of the 2000s, ten Brink and Sheldon reported a series of interesting studies revolving about the use of organoselenium catalysts for oxidations by hydrogen peroxide, systematically investigating the role of the solvent, the role of an additional base and the role of substituents on the organocatalyst.[^213^, ^214^, ^215^]

**Scheme 9:**
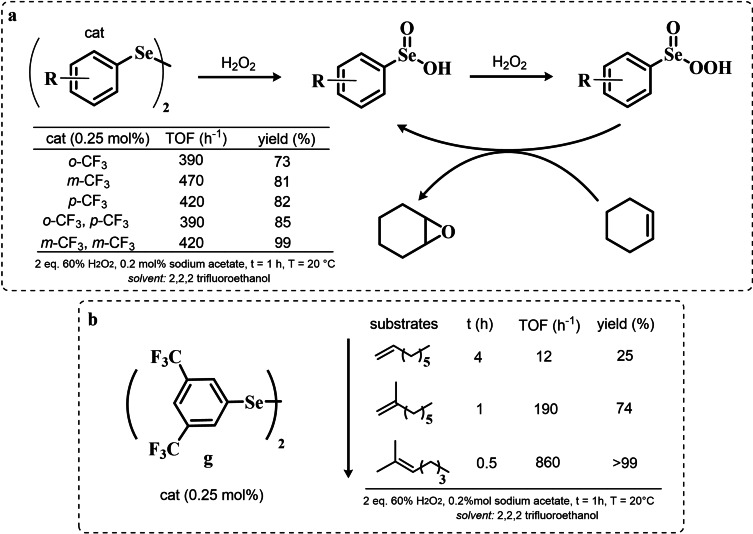
**a** Substituent effect on the diselenide pre‐catalyst (cat) in the organoselenium catalyzed epoxidation of cyclohexene by hydrogen peroxide; **b** Performance of catalyst **g** in the epoxidation of variously mono‐, bi‐, and tri‐substituted olefines.

Particularly, it was observed that by properly optimizing the reaction conditions by addition of 0.2 mol % of a weak base such as sodium acetate and using trifluoroethanol as a solvent, phenyl seleninic acids behave as epoxidation catalysts at room temperature with loadings as low as 0.5 mol %, leading to the effective oxidation of various alkenes in a few hours or less (Scheme 9b).

Importantly, in a systematic study of the substituent effect, it was observed that various substituted phenyl seleninic acids with electron‐withdrawing groups (EWG) on the phenyl rings were all good catalysts for cyclohexene epoxidations.^[214]^ Focusing on the trifluoromethyl substituent (Scheme 9a), the meta substituted acids (*m*‐CF_3_) were found to be generally more reactive than ortho or para substituted analogues (*o*‐ and *p*‐CF_3_). Consequently, meta‐ disubstituted acids were observed to be better catalysts than ortho‐ para‐ disubstituted analogues (*m*‐CF_3,_ *m*‐CF_3_ (catalyst **g**) vs *o*‐CF_3,_ *p*‐CF_3_ in Scheme 9). Similar results were found for nitro substituted phenyl seleninic acids, but these catalysts showed a greater tendency towards epoxide hydrolysis to 1,2 diols, most likely due to an increase in the acidity of the seleninic acid. Additionally, it was reported that the tendency of different alkenes to undergo epoxidation matches to the one reported for the reaction with peroxycarboxylic acids, with mono‐substituted alkenes being the less reactive and tri‐substituted alkenes being more reactive (Scheme 9b).

**Scheme 10:**
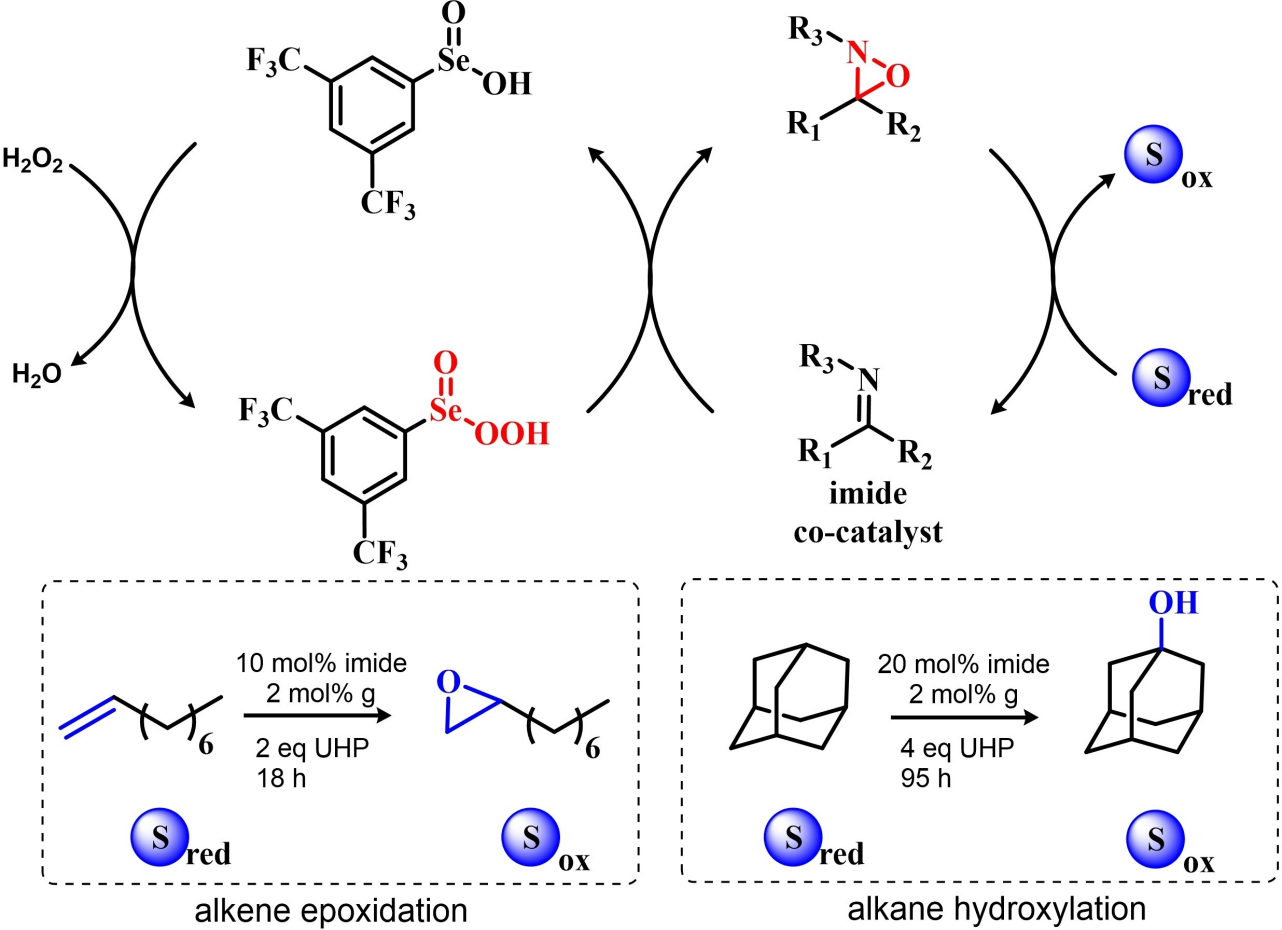
Organoselenium co‐catalyzed oxidation of alkenes to epoxides and of alkanes to alcohols. The organoselenium and active oxidants are highlighted in red.

In 2005, the catalyst **g** optimized by ten Brink and Sheldon was also employed as a co‐catalyst in an interesting study by Brodsky and Du Bois.^[216]^ The authors reported the use of **g** to catalytically oxidize an imine‐based catalyst to an oxaziridine species which acts as the effective oxygen‐transfer agent in alkene epoxidations and alkane hydroxylations (Scheme 10). The system is effective using a urea–hydrogen peroxide (UHP) sacrificial oxidant, but was reported to be ineffective with aqueous hydrogen peroxide. These reactions were found to be effective with as little as 1 mol % of the pre‐catalytic diselenide and 10 or 20 mol % of the imine co‐catalyst. While the performance in the epoxidation of alkenes are less satisfactory when compared against the study of ten Brink (longer reaction times and higher catalytic load are required to lead the reaction to completeness), the hydroxylation of alkanes represents to date the only reported organoselenium‐mediated hydroxylation of alkanes to alcohols.

**Scheme 11:**
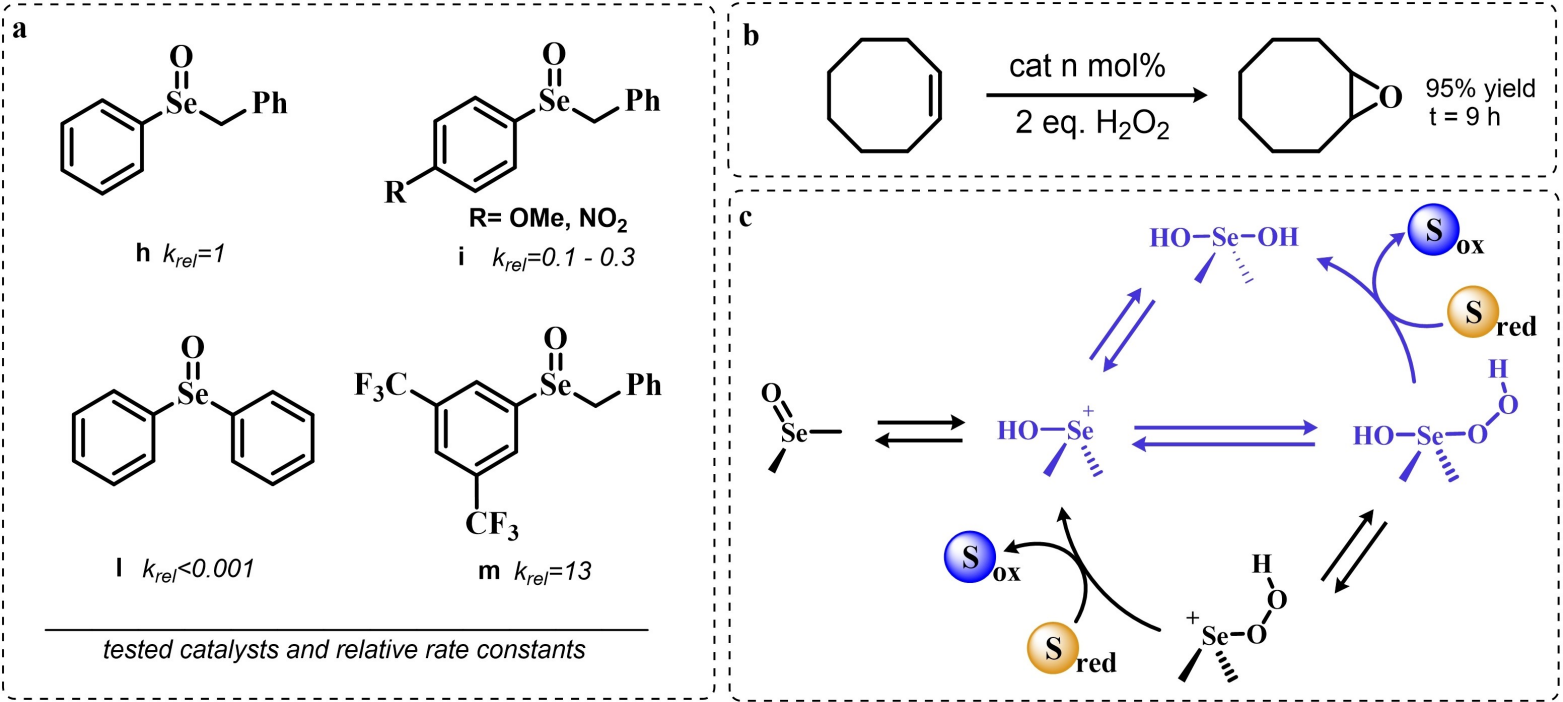
Selenoxides as oxygen‐transfer catalysts. **a,b**. different selenoxides tested by Detty and coworkers as epoxidation catalysts; relative rates (*k_rel_*) of epoxidation of cyclooctene with respect to catalyst **h** were evaluated with 5 mol % of each catalysts. The highlighted yield in cyclooctane epoxide was obtained with 2.5 mol % of catalyst **m; c**. proposed catalytic cycle on the basis of kinetic evidence, the most plausible cycle according to Detty and coworkers is highlighted in blue.

Until the early 2000s, only seleninic acids and their corresponding peroxyacids were implicated in catalytic epoxidations by hydrogen peroxides. Conversely, between 2003 and 2006 Detty and coworkers reported interesting results about the use of selenoxides in the activation of H_2_O_2_,[^217^, ^218^, ^219^] and in 2006, phenyl‐ benzyl‐ selenoxides were found to be active catalysts in epoxidation reactions^[219]^ (Scheme 11a). These species cannot undergo selenoxides elimination reactions for lack of a β‐H with respect to the selenoxide moiety, and thus cannot take part in catalytic mechanisms such as the one shown in Scheme 2. Nevertheless, these species showed promising catalytic activity, even if with lower performance than similar seleninic acids.

Phenyl‐ benzyl‐ selenoxides **h**, **i** and **m** all showed some potential as epoxidation catalyst when employed in 5 mol %, while diphenyl selenoxides was inactive under the same conditions. Interestingly, electronic effects did not seem to affect the reaction in a systematic or easily predictable way, since both EWG and electron‐donating groups (EDG) were found to lower the catalytic potential of these systems (catalysts **i**).^[219]^ Nevertheless, selenoxide **m**, with two EWG *m*‐CF_3_ substituents on the phenyl rings appeared to be the best tested catalyst, in line with substituent studies on phenyl seleninic acids (Scheme 9).

Since previous studies highlighted selenoxides as good oxidants[^220^, ^221^] one might have expected a catalytic cycle in which the selenoxide acts as the actual oxygen‐transfer species being reduced back to a selenide and finally oxidized back to selenoxide by hydrogen peroxide as in the leftmost part of Scheme 2. However, control experiments performed by Detty *et al*. showed that no epoxidation occurred when the selenoxide **m** was employed stoichiometrically without H_2_O_2_ as a co‐oxidant, suggesting that the selenoxide moiety must react with hydrogen peroxide to generate an active oxygen‐transfer agent. Additionally, as expected on the basis of the lack of β‐H, no selenoxide elimination occurred after treatment of the selenoxide **m** with hydrogen peroxide, thus ruling out the possible implication of seleninic acids as catalysts in the reaction. On the basis of these evidences, the catalytic cycle in Scheme 11b was proposed. Since both EWG and EDG hamper the reactivity, a lack of charge development in the rate determining transition state was hypothesized. Thus, the blue path in Scheme 11b was deemed more likely to be the effective catalytic cycle in selenoxide‐mediated epoxidations (An additional discussion on the topic can be found in paragraphs 3.2.2 and 3.3).^[219]^

An important turn in the history of the use of organoselenides in organic chemistry came with the observation that organoselenides reactions can be carried out under green conditions.[^4^, ^222^, ^223^] While the activation of hydrogen peroxide can be considered a green procedure *per se*, organoselenium‐catalyzed alkene functionalization were found to proceed also in water^[224]^ or in green solvents such as glycerol.^[222]^

**Scheme 12:**
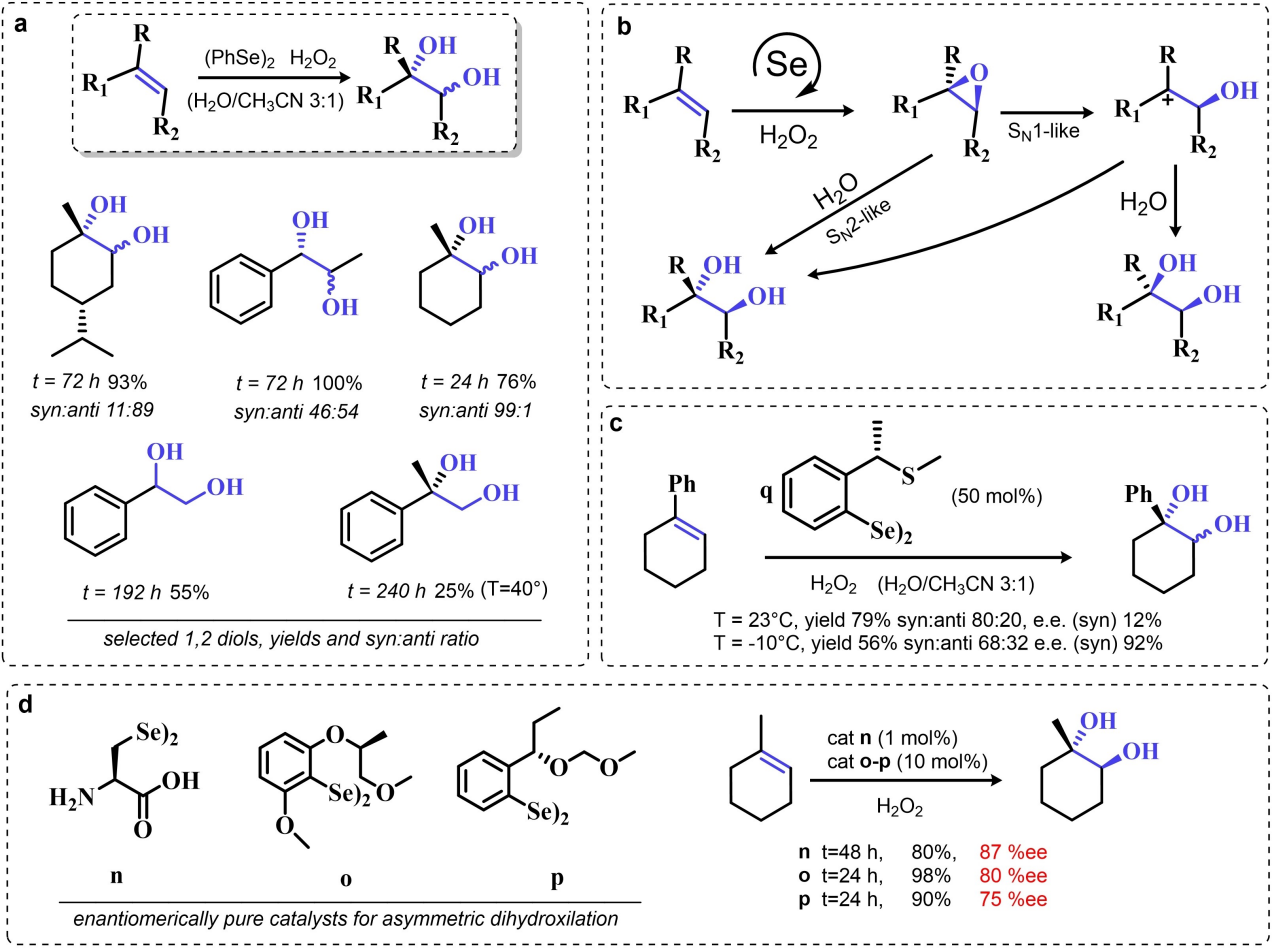
Santi dihydroxylation. **a**. (PhSe)_2_ catalyzed dihydroxylation of substituted alkenes (10 mol % of catalyst). **b**. Reaction mechanism, the epoxidation occurs in line with scheme 9 a. **c** First organoselenium mediated asymmetric dihydroxylation by hydrogen peroxide. **d** selected exempla of organoselenium catalyzed asymmetric dihydroxylations of alkenes by hydrogen peroxide (4 equivalents of hydrogen peroxide were used with catalyst **n**, while 40 equivalents were used with catalysts **o** and **p**.)

Particularly, in 2008 Santi and coworkers reported the green dihydroxilation of alkenes by hydrogen peroxide and the commercially available diphenyl diselenide as precatalyst, thus expanding on the previously described pioneering studies[^202^, ^208^, ^214^] (Scheme 12a). Importantly, the authors observed how both syn and anti 1,2 diols can be produced in both stoichiometric and catalytic conditions, most likely due to two competitive ring opening mechanisms, S_N_1‐like and S_N_2‐like, respectively (Scheme 12b). Despite the somewhat discouraging long times required for the oxidation (from 24–74 h to >150 h depending on the substrate), their study also reported the first ever successful use of a chiral diselenide for the effective asymmetric dihydroxylation of alkenes by hydrogen peroxide (Scheme 12c). Under stoichiometric conditions, it was possible to induce 1‐phenyl cyclohexene dihydroxylation with an enantiomeric excess above 90 % when the reaction was conducted below the freezing point.^[223]^ Unfortunately, under catalytic conditions, only the racemic 1,2 diol was obtained. Nevertheless, a couple of years later, in 2012, some of the same authors showed that it is possible to obtain good to excellent performance in the organoselenium catalyzed asymmetric dihydroxylation of alkenes, by using the chiral diselenides **n–p** ^[224]^ (Scheme 12d). The pre‐catalyst **n**, L‐selenocystine, allowed to carry out the reaction on water, with a pre‐catalyst load as low as 1 mol %. For the other catalysts, the enantiomeric excess was generally lower. Unfortunately, the performance of these catalysts turned out to be somewhat unreliable, with the dihydroxylation of α‐methyl styrene and styrene reaching enantiomeric excess no higher than 20 % and 0 %, respectively. Additionally, by performing the reactions in methanol, the authors reported the use L‐selenocystine pre‐catalyst **n** for the stereoselective hydroxymethoxylation, leading to α‐methoxy alcohols. While α‐methoxy alcohols could be isolated in good yields, the reaction stereoselectivity was greatly reduced when compared against the dihydroxylations of analogous alkenes, most likely due to a parasitic non‐catalyzed path leading to a racemic epoxide intermediate.

A different approach to asymmetric organoselenium catalyzed oxidations of alkenes was investigated in 2013 by Buckley and coworkers,^[225]^ who reported the stereoselective variation of the reaction by Du Bois and coworkers (Scheme 10). In their contribution, the authors used the diphenyl diselenide, which acts as the precursor for the oxygen‐transfer catalyst **a**. The conventional seleninic–peroxyseleninic catalytic cycle was proposed to effectively lead to the oxidation of a chiral imminium co‐catalyst. This species appeared to be an effective asymmetric oxidant leading to epoxides in good yields and with enantiomeric excesses above 80 %. Importantly, differently from the study from Du Bois *et al*., UHP was not required and aqueous hydrogen peroxide provided a good sacrificial oxidant for a variety of asymmetric epoxidations of trisubstituted alkenes.^[225]^

**Scheme 13:**
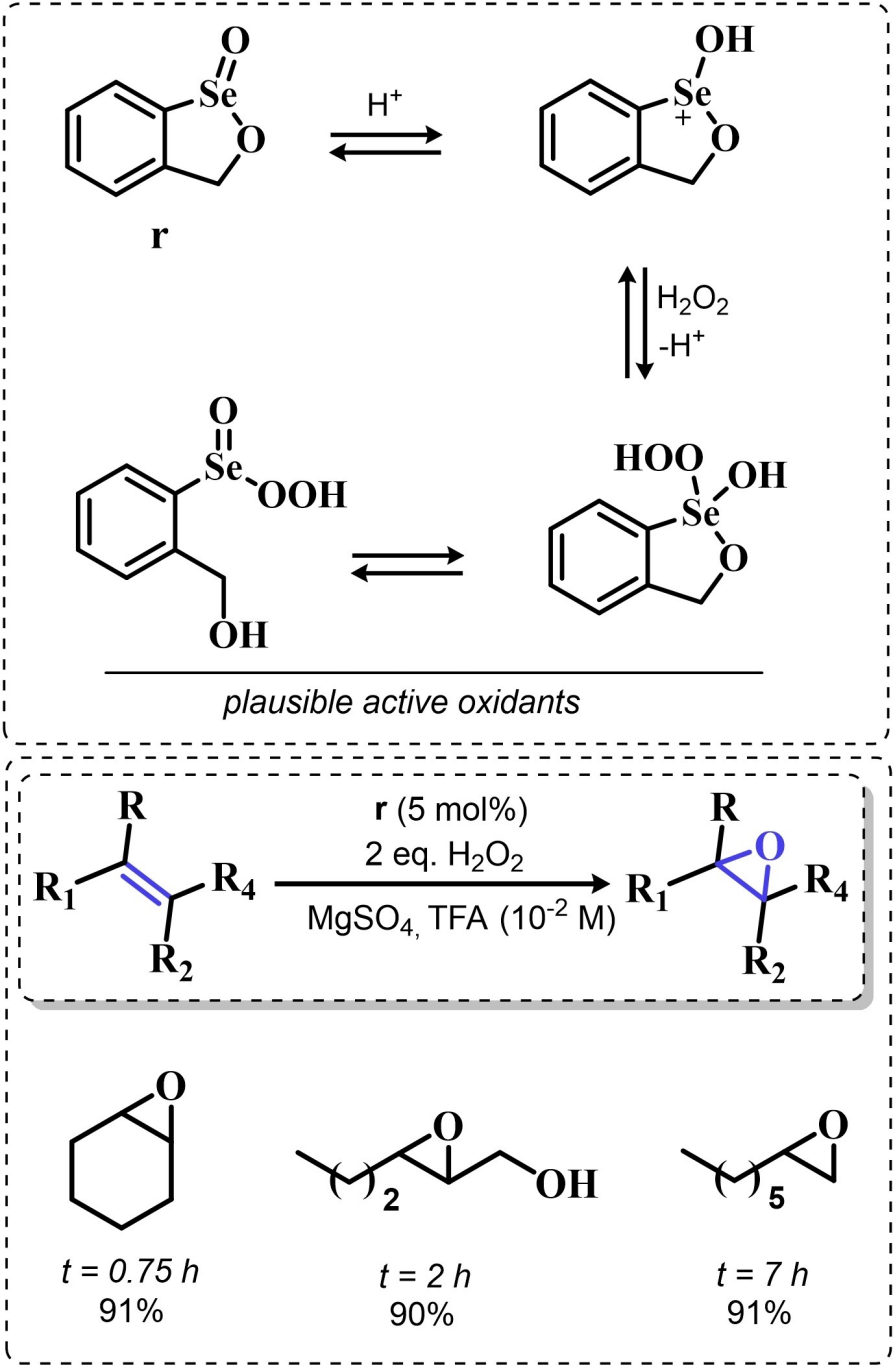
Catalytic epoxidation catalyzed by cyclic seleninate ester **r** in dichloromethane.

In the same period, Back and coworkers reported the effective epoxidation of alkenes with a cyclic seleninate ester **r**. The authors proposed that under oxidative conditions, this species activates hydrogen peroxide either in the form of the hydroxy perhydroxy selenurane proposed by Detty and coworkers or in the form of a conventional peroxyseleninic acid. The authors observed that the addition of trifluoroacetic acid significantly fastens the reaction. In the reaction conditions optimized by Back, partial epoxide hydrolysis to the corresponding 1,2‐diols appeared unavoidable, but catalyst **r** was observed to be more selective for the epoxide than the simple benzene seleninic acid. Particularly, for the oxidation of 1‐methyl cyclohexene, the conditions reported in Scheme 13 led to a 90 : 10 mixture of the corresponding epoxide and 1,2 diol. Conversely, when the reaction was catalyzed by benzene seleninic acid, a 50 : 50 mixture of epoxide and 1,2‐diol was recovered. Intriguingly, styryl derivatives underwent a totally different side reaction, i. e. the oxidative cleavage to the corresponding carbonyl compounds, and the authors provided evidence in favor of an epoxide intermediate in the reaction which then undergoes further reactivity with the organoselenium catalyst *(vide infra*, Paragraph 3.1.3*)*.

More recently, Yu and coworkers screened a variety of aliphatic and aromatic diselenides in the dihydroxylation of cyclohexene, further confirming the privileged nature of the bis‐ trifluoromethyl substituted catalyst **g**.^[226]^ Additionally, the same group expanded the work pioneered by Taylor in 1983 by presenting a polystyrene bound organoselenium catalyst which could effectively oxidize cyclohexene to the corresponding 1,2‐diol. Besides the synthetic value of the catalyst *per se*, Yu and coworkers presented interesting results regarding the reaction mechanism. Particularly, they proved that the catalyst activates not only hydrogen peroxide, but also molecular oxygen to some extent. Most strikingly, by means of XPS spectroscopy, the oxidized form of the catalyst was found compatible with the presence of the selenium atom in the oxidation states +2 and +6 within the resin, as opposed to the expected oxidation state +4 almost exclusively invoked in the analogous homogeneous catalytic cycle. This result is not completely general, since other XPS investigations of other classes of immobilized phenyl seleninic acids were found consistent with a selenium nucleus in the oxidation state +4 after exposure to hydrogen peroxide.^[227]^

**Scheme 14:**
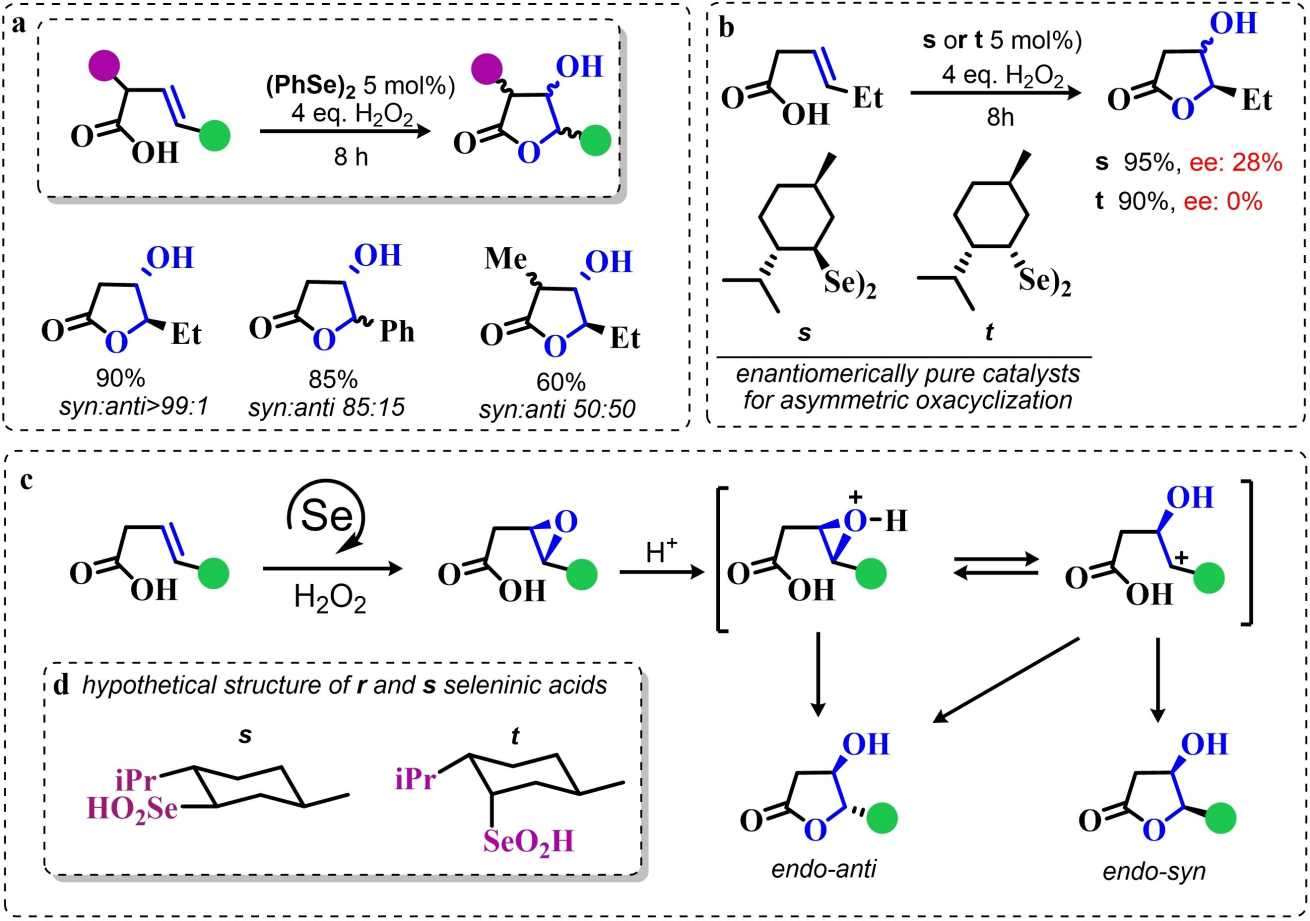
Organoselenium catalyzed oxacyclization of alkenoic acids. **a** reaction conditions, selected cyclic products, yields and diasteromeric ratio. **b** organoselenium catalyzed stereoselective oxacyclization catalyzed by enantiomerically pure diselenides. **c** proposed reaction mechanism. **d** proposed structure of optically pure catalysts to explain the different enantiomeric excesses.

Santi and coworkers also reported on the organoselenium catalyzed oxacyclization reaction of alkenoids acids, which proceeds via an organoselenium mediated epoxidation and intramolecular ring opening reactions with 5*‐endo*, or 6‐ and 5*‐exo* cyclizations (Scheme 14).[^228^, ^229^]

5 mol % of diphenyl diselenide is enough to carry out the reaction in 8 hours, on water and at room temperature, leading to 60–90 % yield in the cyclic compound. They proposed an organoselenium mediated process in line with Scheme 9a, and then two different epoxide ring‐openings with concomitant cyclization to explain the different diasteroselectivity observed (Scheme 14c). Particularly, when the *5‐endo* cyclization occurs on substrates capable of stabilizing carbocationic species, it can lead partly to the endo‐syn product, otherwise the endo‐anti product is favored (Scheme 14a). *5‐* and *6‐exo* cyclizations have also been observed depending on the position of the double bond on the substrate.^[228]^ Generally, *5‐endo* products are favored, while *5‐exo* cyclization is favored over *6‐endo*. The same authors also explored the use of flow chemistry to carry out the reaction, in the first reported use of organoselenium catalysis under flow conditions. The procedure was found effective for the synthesis of a variety of lactones and cyclic ethers under mild conditions.^[229]^ Attempts to develop a stereoselective variant of the oxacyclization reactions were made by the same authors (Scheme 14b), by using the optically pure diterpenyl diselenides **s** and **t**. While both catalysts effectively carried out the oxacyclization, only a moderate stereoselectivity was observed for catalyst **s**, while catalyst **t** led to the racemic mixture of the products. This outcome was rationalized hypothesizing a different steric effect in the vicinity of the selenium atom in the two catalysts, as shown in Scheme 14d.

Interestingly, Garcia and coworkers observed that benzene seleninic acid can activate hydrogen peroxide for the effective oxidation of substituted naphtols to naphtoquinones. The proposed reaction mechanism is based on the conventional seleninic–peroxyseleninic catalytic cycle, in which the peroxyseleninic acid oxidizes naphtol to an epoxide which is lastly converted to the corresponding quinone by air.^[230]^ A similar reaction was studied in 1983 with tBuOOH as oxidant by Taylor and coworkers,^[208]^ but experimental evidence for the occurrence of this reaction via a selenium‐mediated epoxidation are still missing.

**Scheme 15:**
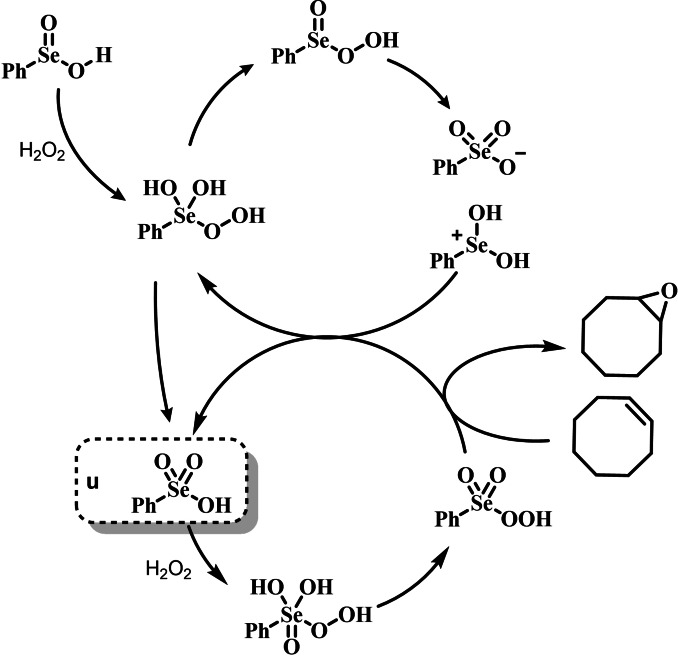
Back *et al*. proposal for Se(VI) selenonic acid catalyzed epoxidation of cyclooctene.

Up until 2020, the seleninic–peroxyseleninic acid catalytic cycles dominated the field of homogeneous organoselenium catalyzed epoxidation and dihydroxilations. Apart from the report of +2 and +6 oxidation state in the immobilized phenyl seleninic acids, only the +4 oxidation state of selenium had been invoked in epoxidation and dihydroxylation reactions occurring in solution. Quite recently, the supremacy of the +4 oxidation state has been challenged by Back and coworkers. Interestingly, by reviewing the diphenyl diselenide mediated oxidation cyclooctene by hydrogen peroxides, first pioneered by Sharpless and Hori, it was observed that the benzene seleninic acid produced *in situ* by oxidation of the diselenide was converted to a selenonium selenonate salt in which Se is in the oxidation states +4 and +6. This salt was found to further react with hydrogen peroxide at 60 °C, leading to the formation of benzene selenonic acid **u**. Both the salt and selenonic acid were found to be more active than seleninic acid in the epoxidation of cyclooctene with hydrogen peroxide. These observations led the authors to hypothesize a Se(VI) based catalytic cycle for the organoselenium mediated epoxidation of alkenes, in which the selenonic acid is produced *in situ* by a step‐wise isomerization of the corresponding peroxyseleninic acid (Scheme 15). Whether Se(VI) is the active oxidation state also under catalytic conditions, or for other organoselenium mediated oxidation, is still a topic of investigation.

#### 3.1.2 Organoselenium Catalyzed Baeyer‐Villiger of Ketones and Aldehydes

**Scheme 16:**
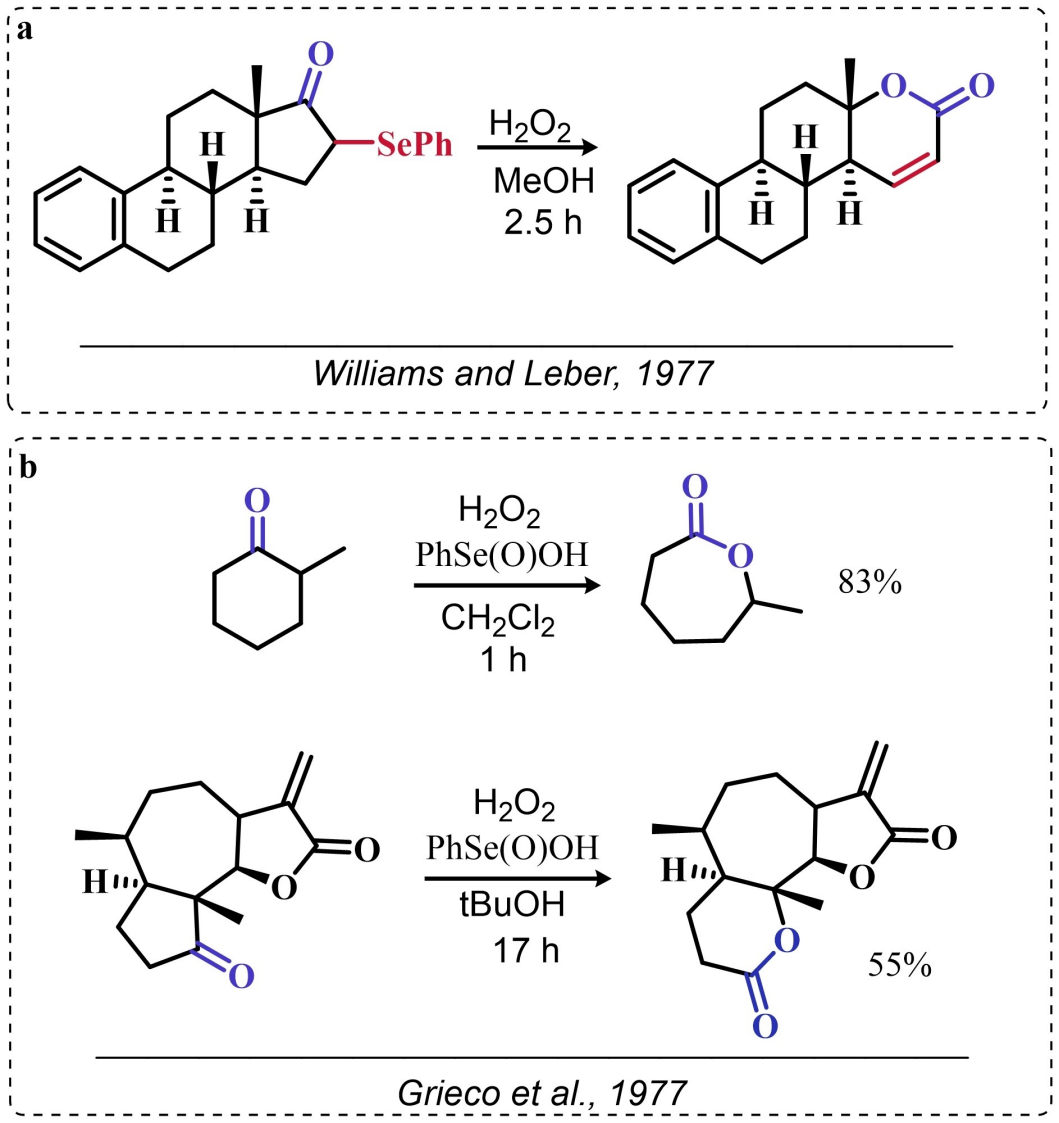
Pioneering organoselenium‐mediated Baeyer‐Villiger reactions of cyclic ketones. **a** serendipitous discovery by Williams and Leber, and **b** Systematic investigation with simple and complex ketones by Grieco and coworkers.

Organoselenium mediated Baeyer‐Villiger reactions were independently discovered by Williams and Grieco in 1977.[^231^, ^232^] Williams’ discovery was serendipitous, and it occurred in an attempt to synthesize the correspondent α,β unsaturated ketone, in a manner not dissimilar by the fortuitous discovery of organoselenium mediated epoxidations reported in Scheme 4. Conversely, Grieco *et al*. study was the first fully dedicated to the reaction^[232]^ (Scheme 16).

Particularly, the authors reported a variety of cyclic and polycyclic ketones oxidation to the corresponding lactones, highlighting the possible hydrolysis to hydroxy carboxylic acids. For most of the investigated systems, the reactions occurred at room temperature in couple of hours, and the active oxidant was deemed to be the benzene peroxyseleninic acid previously postulated by the same authors in the organoselenium mediated epoxidation.^[204]^

Following this pioneering study, Taylor and coworkers employed the same polystyrene bound seleninic acid previously described for epoxidations (Scheme 7) to carry out a variety of Baeyer‐Villiger reactions of alkenes. Cyclobutanone, particularly, reacted readily in three hours to produce the corresponding gamma‐butyrolactone in a 96 % yield.^[208]^ The oxidation of aldehydes was not attempted.

**Scheme 17:**
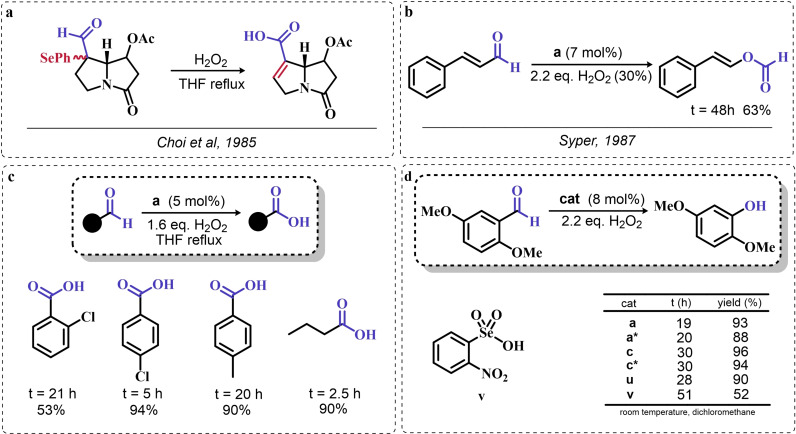
Pioneering results on organoselenium‐catalyzed aldehyde oxidations. **a** First reported organoselenium‐mediated oxidation of aldehydes to carboxylic acids. **b** Oxidation of α,β unsaturated aldehydes to formyl esters catalyzed by benzene seleninic acid. **c** Oxidation of substituted aromatic and aliphatic aldehydes to carboxylic acids catalyzed by benzene seleninic acid. **d** Role of the catalyst in organoselenium catalyzed oxidation of 2,5‐dimethoxy benzaldehyde. Catalysts marked as * were generated *in situ* starting from the corresponding diselenide.

To the best of our knowledge, the first reported organoselenium mediated oxidation of aldehydes to carboxylic acids was possibly reported by Choi and coworkers in 1985.^[233]^ The protocol closely matches to the serendipitous discovery of the oxidation of cyclic ketones to lactones by Williams *et al*. Indeed, Choi and coworkers observed the oxidation of an α,β unsaturated aldehyde generated via selenoxides elimination of the corresponding α‐selenylated aldehyde (Scheme 17a). Then, in the late 1980s, the field of organoselenium catalyzed oxidation of aldehydes and ketones by hydrogen peroxide was independently developed by the groups of Syper[^234^, ^235^] and Choi^[236]^ (Scheme 17b–d).

The authors observed two very different kinds of reactivity. In apparent contrast with the results of Choi *et al*., Syper reported in 1987 the oxidation of cinnamaldehyde and other α,β unsaturated aldehydes to the correspondent formyl esters^[234]^ (Scheme 17). The reaction took place in two days at room temperature when benzene seleninic acid was used as a catalyst. Importantly, the formyl ester was observed to be able to undergo hydrolysis to the corresponding alcohol or undergo further reactivity, especially when a higher concentration of hydrogen peroxide was used (90 %) or more acidic catalysts were employed (e. g., catalyst **c**). Conversely, Choi *et al*. observed the oxidation of aldehydes to the corresponding carboxylic acids, and it was observed that EWG facilitates the reaction, while EDG and steric hindrance in the surrounding of the catalyst disfavors it. Indeed, in a nice comparison, Syper *et al*. observed that *o‐*tolualdehyde is oxidized to the corresponding formyl ester, which then undergoes hydrolysis to *o*‐methyl phenol. Conversely, *p*‐tolualdehyde is slowly oxidized to the corresponding acid, as observed by Choi *et al*. (Scheme 17c). Additionally, Choi and coworkers also observed that aliphatic aldehydes can also be oxidized to the corresponding acids. Most importantly, starting from *o‐ p*‐ EDG substituted aldehydes (Scheme 17d), Syper investigated how different organoselenium acids catalyzed their oxidation to the formyl derivatives and further hydrolysis to the substituted phenols. Differently from epoxidations, nitro substituted benzene seleninic acids appeared to be worse catalysts then unsubstituted ones. Another important result is that Se(VI) phenyl selenonic acids appear to be systematically worse catalysts than their analogous Se(IV) phenyl seleninic acids (e. g., **a** vs **u, c** vs **v** in Scheme 17). Additionally, the reaction was less selective, and part of the starting aldehyde gave products different from the substituted phenols. On the basis of the recent observation by Back and coworkers,^[237]^ it seems reasonable that the selenonic acid identified by Syper and coworkers might have been the selenonium selenonate salt characterized in 2020. Nevertheless, the result by Syper provide the first evidence that the lower oxidation state Se(IV) has better catalytic performances than the higher Se(VI) in aldehyde oxidations. Additionally, the catalytic performances of seleninic acids and the corresponding diselenides appeared to be quite similar and highly different from analogous selenonic acids, thus suggesting that under the reaction conditions in which Syper operates, diselenides are not overoxidized to the high Se(VI) oxidation state.

**Scheme 18:**
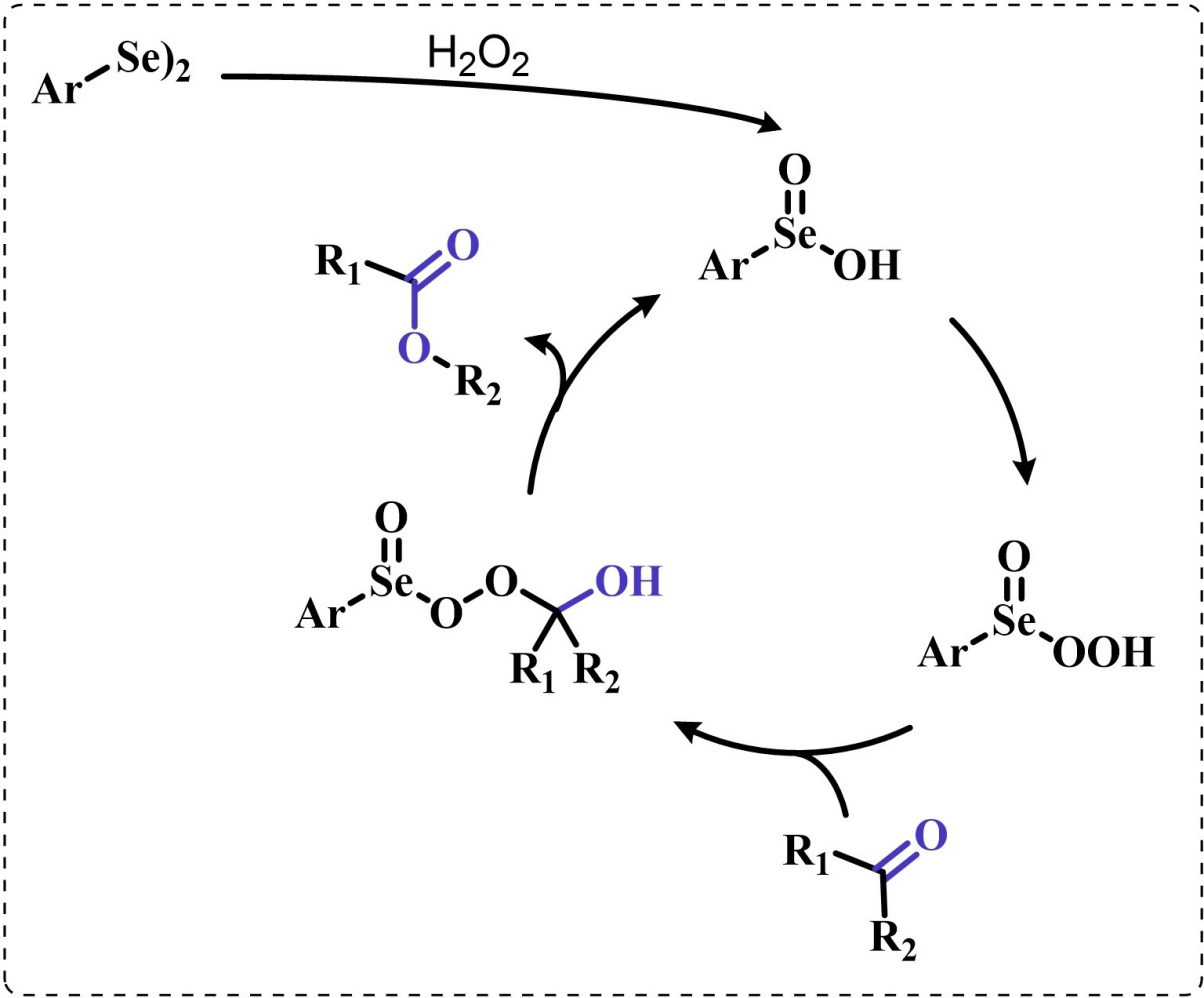
Reaction mechanism proposal by Sheldon *et al*. for organoselenium mediated BV reaction of ketones and aldehydes.

In 2001, a systematic study by Sheldon and coworkers, who previously investigated applications of diselenides as catalysts in epoxidation, led to the conclusion that catalyst **g** is also suitable catalyst for ketones and aldehydes oxidations. By using this catalyst and the 2,2,2‐trifluoroethanol as solvent, they reported the effective quantitative conversion to the corresponding BV products of a series if aldehydes and ketones with good to excellent selectivity. Most aldehydes and ketones were found to react to completeness in 0.5–4 hours, with some challenging substrates requiring longer times.^[213]^ For aldehydes, the authors also further highlighted how electron‐rich aromatic aldehydes are smoothly oxidized to the corresponding formyl esters which then hydrolyzes to the corresponding substituted phenol. Conversely, electron poor aromatic aldehydes are converted to the corresponding acids, in nice confirmation of the pioneering results of Syper and Choi (Scheme 17). Additionally, a plausible reaction mechanism for the BV oxidation was provided by the authors in analogy with the corresponding reaction mechanism of peroxycarboxylic acids (Scheme 18). The authors propose the initial activation of the diselenide pre‐catalyst in the form of a phenyl seleninic acid, which lastly activates hydrogen peroxide in the form of the corresponding peroxyacid. This species nucleophilically attacks the carbonyl carbon, leading to the formation of a tetrahedral intermediate which lastly transfers the oxygen intramolecularly (see also Scheme 3). To the best of our knowledge, this catalytic cycle is, up to date, the only mechanistic proposal for carbonyl oxidations by phenyl seleninic acids and precursors. The same authors extended their investigation also to BV reaction carried out under bi‐ and tri‐phasic reaction conditions by using a modified version of catalyst **g**, to allow an easy catalyst recovery and reuse.^[215]^

**Scheme 19:**
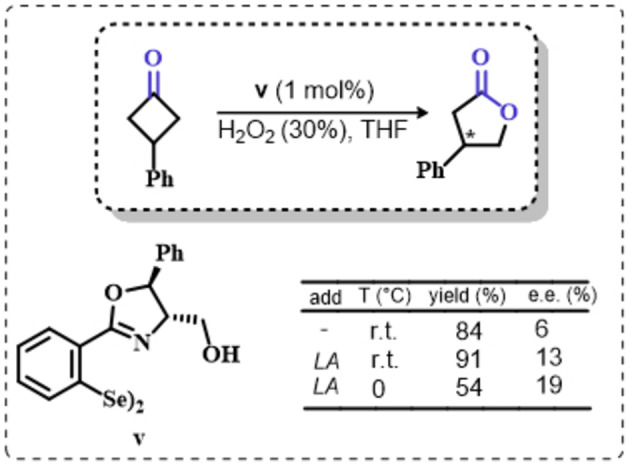
Asymmetric Baeyer‐Villiger reaction catalyzed by chiral diselenide **z** and Yb(OTf)_3_ as Lewis acid (LA, 2 mol %).

In the same period, Uemura and coworkers attempted the first organoselenium‐catalyzed asymmetric BV reaction of 3‐phenyl butanone (Scheme 19), by using chiral diselenide **z**. Unfortunately, only low level of enantioselectivity were observed.

Addition of a metal‐based Lewis acid moderately increased the enantioselectivity, likely via formation of a chiral complex with the seleninic acid, however enantiomeric excesses not higher than 20 % were observed even by reducing the temperature down to 0 °C. The possible racemization of seleninic acids, discovered in the same period by Kamigata and coworkers, was mentioned as a possible origin of the low asymmetric induction.^[238]^

Interestingly, while electron poor catalysts dominated the field of organoselenium‐catalyzed oxygen‐transfers, Ichikawa and coworkers reported in 2005 on a new *o‐*triflate substituted diselenide with good performance in the BV reactions with hydrogen peroxide. Particularly, the catalyst was tested against the oxidation of the 2,5 dimethoxyaldehyde previously oxidized by Syper (Scheme 17). While diphenyldiselenide required ca. 20 h to convert the aldehyde to the corresponding phenol, the *o*‐triflate substituted diselenide carried out the reaction in ca. 3 h under similar conditions. A variety of cyclic ketones were also oxidized to the corresponding lactones in 8–24 h depending on the substrate.^[239]^

**Scheme 20:**
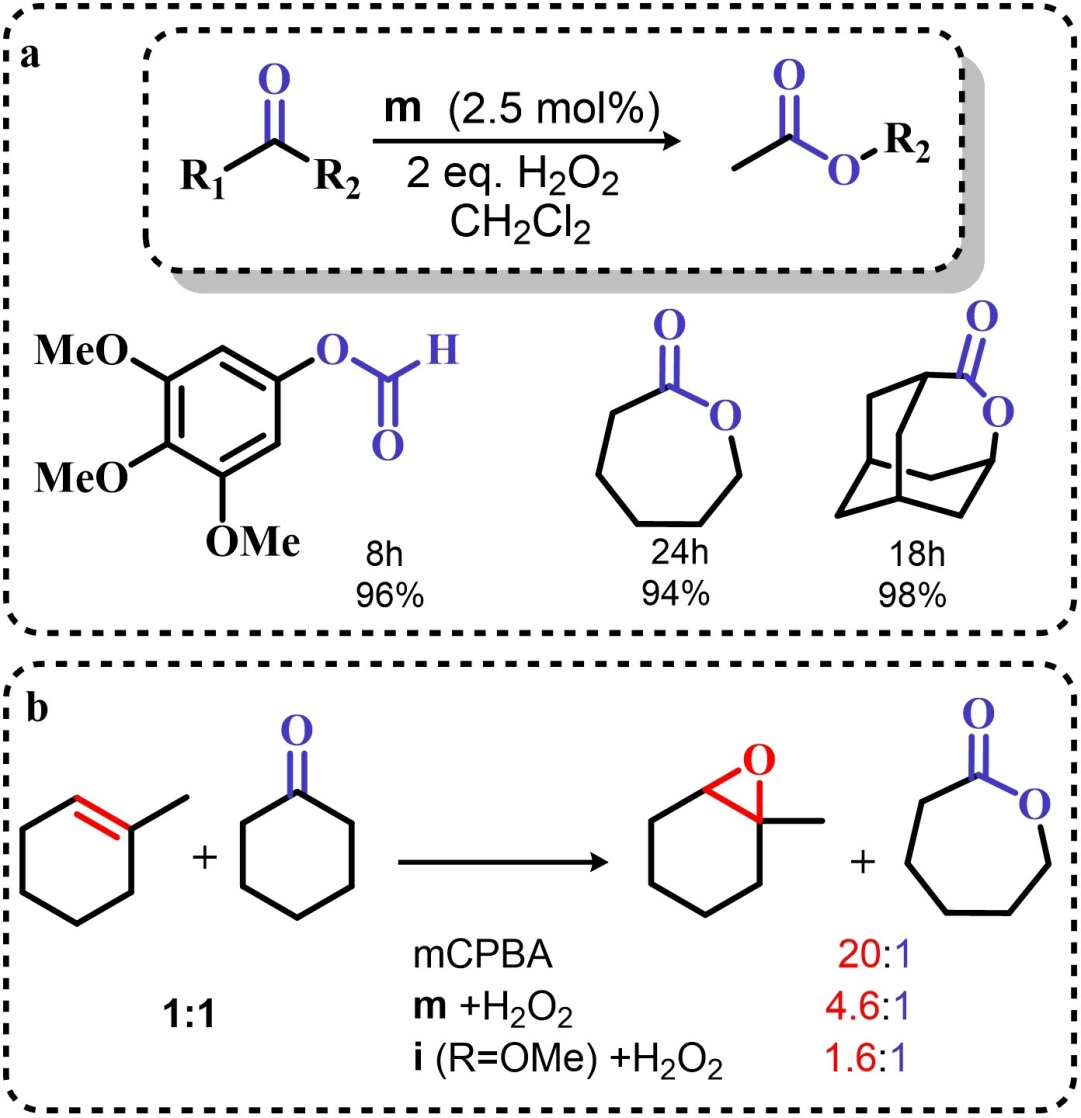
Detty *et al*. investigation of selenoxides catalyzed Baeyer‐Villiger reactions. **a** Oxidation of aldehydes and ketones under optimized conditions. **b** Competitive oxidation of alkenes and ketones by catalyst **m** and **i**, and comparison with *m*‐CPBA oxidations.

As previously discussed for epoxidation, selenoxides *per se* can also be active catalysts for Baeyer‐Villiger reactions, that is, without undergoing β‐elimination reactions to generate phenyl seleninic acids (Scheme 16a). Indeed, Detty and coworkers reported that the same phenyl benzyl selenoxides active for epoxidations, (catalysts **h–m** in Scheme 11a) can effectively carry out the oxidation of ketones and aldehydes to esters^[219]^ (Scheme 20a). Importantly, the electron poor catalyst **m**, the selenoxides analogue of the diselenide **g**, was found to be the most active among the investigated catalysts. However, under the optimized conditions, the selenoxides catalyzed BV reactions were slower than corresponding diselenides catalyzed ones performed by Sheldon and coworkers using catalyst **g**.^[213]^ Most importantly, Detty and coworkers tested the different selectivity in the oxidation of ketones and alkenes by selenoxides and hydrogen peroxide, as compared to oxidations carried out with *m*‐CPBA (Scheme 20b). While their selectivity ratios are certainly bounded to choice of esanone and 1‐methyl cyclohexene as substrates, and more activated ketones or deactivated alkenes might change the results, the results presented show a decreased selectivity in the selenoxides catalyzed oxidation of these functional group. Indeed, while *m*‐CPBA strongly favors epoxidation over BV, this preference is strongly reduced with the most active catalyst **m**, and almost disappears with the less active and electron rich catalyst **i**. To the best of our knowledge, a similar analysis for oxidations mediated by phenyl seleninic acid has not been performed.

**Scheme 21:**
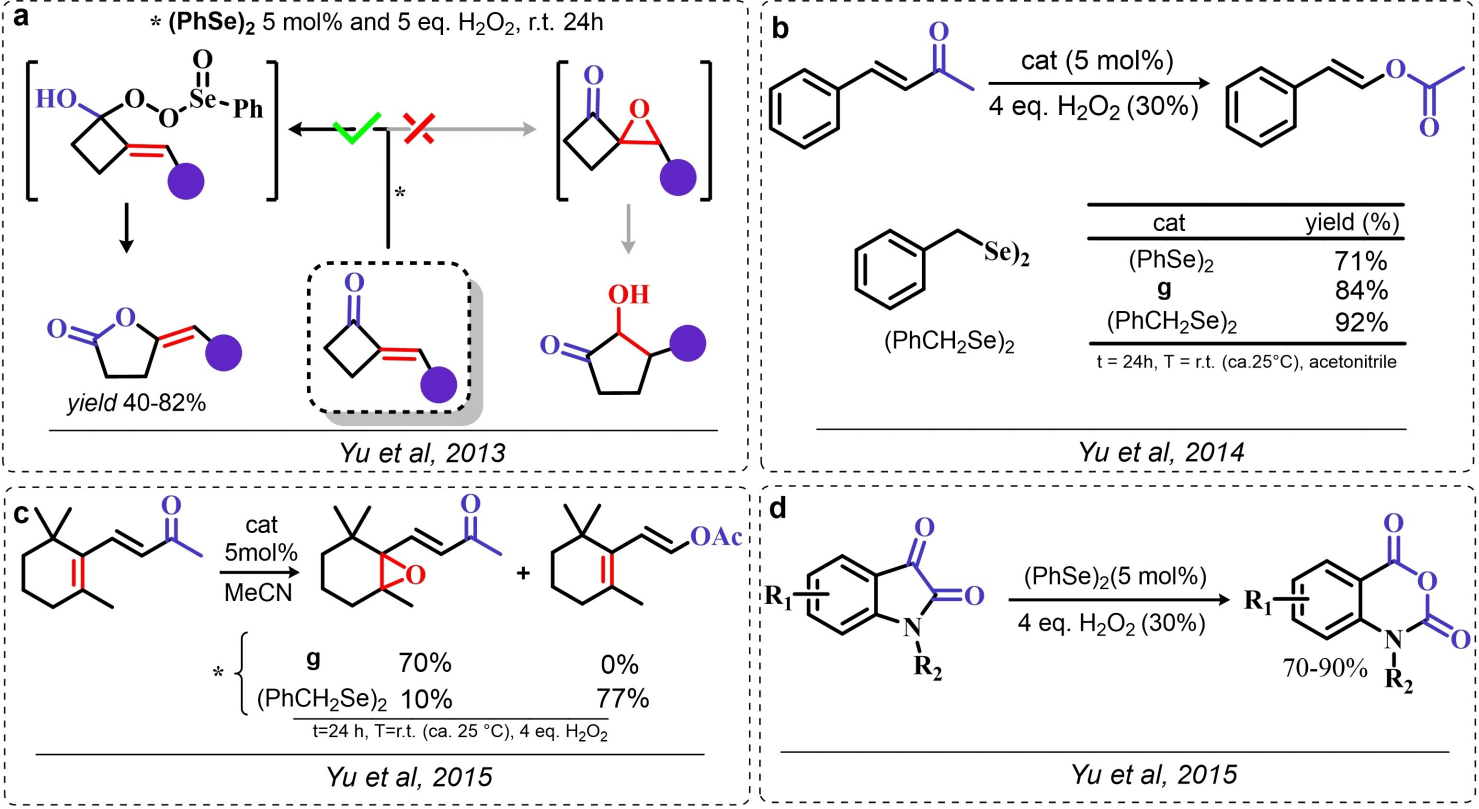
Yu and coworker's investigation on the scope of organoselenium catalyzed BV oxidations. **a** Selective oxidation of 2‐methylene cyclobutanone. **b** Oxidation of α,β unsaturated ketone to allyl esters; **c** Oxidation of β‐ionone to the corresponding epoxide or allyl ester different diselenides; **d** Oxidation of isatin to corresponding anhydride.

Around the mid‐2010s, the group of Yu presented some interesting contributions for organoselenium catalyzed carbonyl oxidations, especially BV ketone oxidations, providing further insight into the selectivity of various seleninic acids against different functional groups. Particularly, it was observed that diphenyl diselenide specifically targets the carbonyl function instead of the C=C bond in variously substituted 2‐methylene cyclobutanones leading to the corresponding lactones^[240]^ (Scheme 21a). The pentacyclic α‐hydroxy ketone deriving from the plausible alternative epoxidation reaction was not observed. Additionally, the authors observed that while both diphenyl diselenide and the electron poor catalyst **g** are indeed good catalyst in the BV oxidation of α,β unsaturated ketone to vinyl esters, as previously observed,^[213]^ dibenzyl diselenide appeared to be an even better catalyst^[241]^ (Scheme 21b). This result is even more interesting under the perspective that phenyl seleninic acids dominated the field of organoselenium catalyzed oxygen‐transfer, while aliphatic analogues usually displayed worse performances^[207]^ and have been generally less investigated. The catalytic power of dibenzyl diselenide was further applicated in 2015 in a study about the selective oxidation of β‐ionone^[227]^ (Scheme 21c). Indeed, β‐ionone has two double bonds conjugated to a carbonyl function, and all three functions might interact with peroxyseleninic acids leading possibly to a variety of compounds, such as epoxides or esters. Yu *et al*. observed that the electron poor catalyst **g** leads to the selective epoxidation of the endocyclic double bond, while dibenzyl diselenide preferentially leads to the BV oxidation of the carbonyl function to the corresponding allyl ester. These results highlighted the possibility of tuning the regioselectivity of organoselenium‐catalyzed oxygen‐transfers to specifically target different functional groups with different pre‐catalysts. Lastly, the authors applied diphenyl diselenide as a catalyst to convert isatin to the corresponding anhydride, thus allowing the use of hydrogen peroxide to perform this oxidation (Scheme 21d). Importantly, the capacity of seleninic acids derived from diselenides to oxidize 1,2 diketones to anhydrides was also hypothesized by Mlochowsky and coworkers in 2008 in the context of multistep, domino degradation reaction.^[242]^

**Scheme 22:**
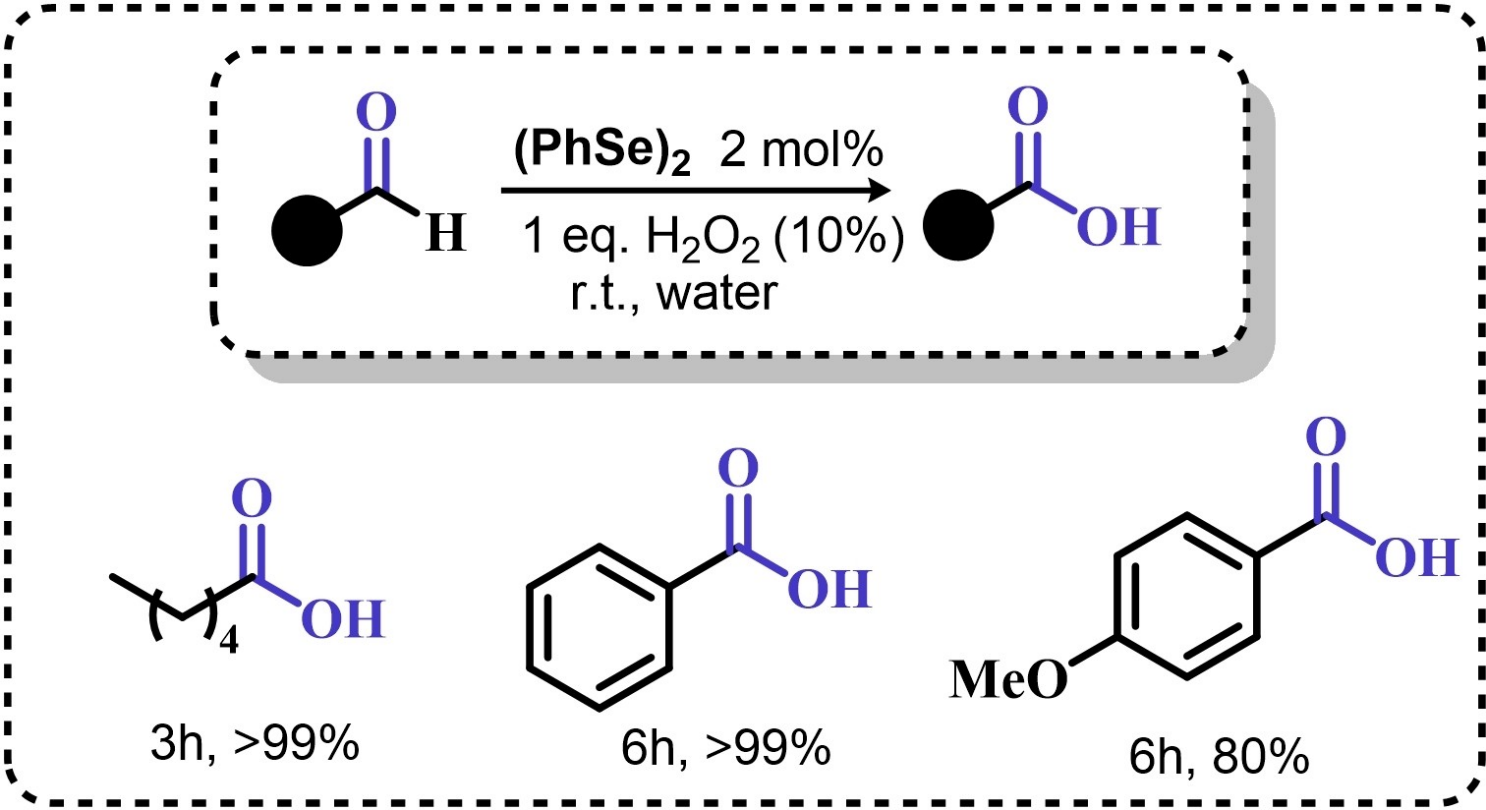
Organoselenium catalyzed oxidation of aldehydes to carboxylic acids under on water conditions.

In their effort of turning organoselenium chemistry greener, Santi and coworkers reported in 2015 the possibility of performing the diphenyl diselenide catalyzed oxidation of aldehydes to carboxylic acids under on water conditions.^[243]^ The protocol, which further expands and develops the pioneering work of Choi and coworkers, provided good yield in the corresponding carboxylic acids using as little as 2 mol % of catalyst, and with only one equivalent of relatively diluted (10 %) hydrogen peroxide. Interestingly, under these conditions, it was possible to oxidize to carboxylic acids also electron rich aldehydes that usually undergo oxidation to formyl esters and partial or total hydrolysis to substituted phenols in other solvents (Scheme 22).

Additionally, the authors proved that by performing the reaction in methanol and heightening the concentration of hydrogen peroxide to 30 % in the attempt to esterify the acids produced by the organoselenium catalyzed oxidations, electron rich aldehydes such as *p*‐methoxy benzaldehyde are only moderately oxidized to the corresponding acids. Conversely, the main product of the reaction is either the formyl ester (at low temperatures) or directly the hydrolyzed phenol (at high temperatures), as previously observed by Choi and Syper. A Dakin‐like reaction was deemed responsible for this different behavior. Whether these different reactivities are mediated by different organoselenium active oxidants, or simply by a change in the experimental conditions, has never been investigated. According to the authors’ hypothesis, the reaction proceeds within the conventional seleninic–peroxyseleninic catalytic cycle (Scheme 18), where the benzene seleninic acid is derived from the *in situ* oxidation and cleavage of the Se−Se bond of diphenyl diselenide. Importantly, in the attempt to provide evidence in support of this hypothesis, the author treated 1 eq. of diphenyl diselenide with 5 eq. of hydrogen peroxide, and the obtained NMR spectra showed three different signals, among which only the benzene seleninic acid could be precisely assigned.^[243]^ Moreover, addition of a radical scavenger completely hampered the reaction, suggesting a radical step in part of the mechanism. A consistent mechanistic picture is still to be proposed to date.

Most recently, these reactivities have been performed also via immobilized organoselenium compounds, mostly phenyl seleninic acids. Particularly, via a resin bound phenyl seleninic acids, organoselenium‐catalyzed BV reaction of lignin‐derived ketones have been performed, leading to the corresponding esters and, via hydrolysis, to the corresponding phenols in moderate to good yields 50–90 %, at room temperature and in low times (15 minutes to 4 h).^[244]^ Additionally, phenyl seleninic acids immobilized on magnetic nanoparticles proved to be active catalysts for aldehyde oxidations to carboxylic acids.^[245]^ Most importantly, via an XPS analysis, the oxidation state of the catalyst pre‐treatment with hydrogen peroxide was found to be +2, while it turned to +4 after treatment with hydrogen peroxide. This result is compatible with the active role of phenyl seleninic acids in the catalytic cycle. Organoseleninic acids incorporated in microgels were also found to be capable of activating hydrogen peroxide to perform aldehydes oxidations to carboxylic acids, and IR, NMR and Raman spectroscopy data were in support of the formation of seleninic acid within the microgel, in compliance with a Se(IV) based catalytic cycle.^[246]^

As a final note, it must be mentioned that carbonyl compounds can undergo side reactions during organoselenium catalyzed BV oxidations, the most important of which is a ring contraction reaction leading to an *exo*‐ carboxylic acid. This reaction was first explored in 1999 by Syper and coworkers, and it was mentioned along the year as the origin of some of the byproducts of organoselenium catalyzed oxidations of carbonylic species. To the best of our knowledge, precise mechanistic evidence to build a reasonable mechanism for the reaction are still missing, but it is hypothesized that it proceeds via the addition of the organoselenium moiety to the enolate form of carbonyl species.[^247^, ^248^]

#### 3.1.3 Organoselenium Catalyzed C=C Bond Breaking

**Scheme 23:**
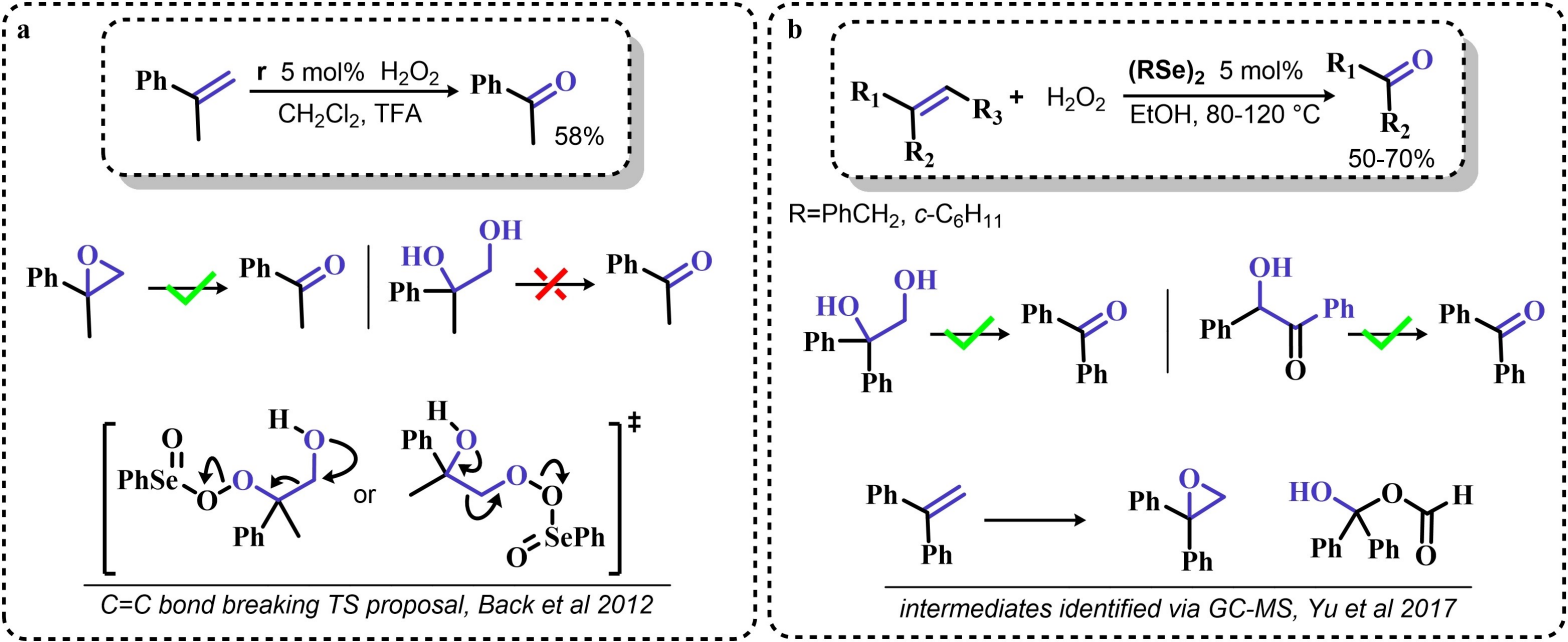
Organoselenium catalyzed C=C bond breaking. **a** Synthesis of acetophenone with cyclic seleninate **r** at room temperature, control experiments and proposed transition states by Back *et al*.; **b** Yu *et al*. C=C bond cleavage of di and trisubstituted alkenes, control experiments and GC‐MS identified intermediates.

In the light of the previous discussion, the past fifty years of research showed that electron deficient aryl seleninic acids are good catalysts for epoxidation and dihydroxylation reactions and, in general, for reactions in which the organoselenium catalysts act as the electrophile in the S_N_2‐like oxygen transfer mechanism. Conversely, benzyl seleninic acids are good catalysts for Baeyer‐Villiger reactions and, possibly, for reaction mechanisms in which the organoselenium peroxyacid nucleophilically attacks the organic substrate. There are cases in which both mechanisms can co‐exist within the same reaction. One interesting case is the organoselenium‐catalyzed C=C bond breaking with hydrogen peroxide. While this process is not an oxygen‐transfer *per se*, the reaction is currently believed to proceed via a series of organoselenium‐catalyzed oxygen transfers. To the best of our knowledge, the first evidence that organoselenium species can cleave C=C bonds is due to Back and coworkers, who observed this kind of reactivity as a side reaction during the epoxidation of some alkenes in 2012^[249]^ (Scheme 23).

During their application of the cyclic seleninate **r** to epoxides a variety of olefines (Scheme 13), Back and coworkers observed the production of acetophenone starting from 1,1 methyl‐phenyl ethylene. In a series of control experiments, they observed that under their experimental conditions, only the epoxide reacts to the same product; conversely, the 1,2 diol produced by epoxide hydrolysis does not react under the same condition, thus ruling out its participation in the CC bond breaking reaction. On the basis of these observations, two possible C=C bond breaking transition states were proposed, both formed by the nucleophilic attack of the peroxyseleninic acid to the epoxide moiety (Scheme 23a).

A couple of years later, Yu and coworkers developed an organoselenium catalyzed C=C methodology for the cleavage of the C=C bond in di‐ and tri‐substituted alkenes[^250^, ^251^] (Scheme 23b). Conversely to Back *et al*. methodology, their protocol requires high temperature (80–120 °C) to occur but allows the conversion of a more general number of substrates. Importantly, they observed that with both aryl and alkyl diselenides average yield in carbonyl compounds could be obtained (ca. 50–60 %), with the latter giving slightly better performances. Dibenzyl diselenide (PhCH_2_Se)_2_ and dicyclohexyl diselenide (c‐C_6_H_11_Se)_2_ in the presence of excess hydrogen peroxide (5 eq.) were both good catalysts depending on the substrate, but somewhat long reaction times (48 h) were required to carry out the cleavage of most olefines. Most importantly, the authors provided GC‐MS data and control experiments to clarify the reaction mechanisms. (Scheme 23b) Contrarily to the results provided by Back and coworkers, under the conditions optimized by Yu *et al*., vicinal diols were cleaved to the corresponding carbonylic compounds, and are thus plausible intermediates in the reaction. Similarly, on the basis of control experiments, α‐hydroxy ketones were hypothesized as reaction intermediates. Lastly, both epoxides and α‐hydroxy esters/acids, were identified via GC‐MS investigations, and were thus considered as plausible intermediates. On the basis of these data, Yu *et al*. proposed that the alkenes are firstly oxidized to epoxides and 1,2 diols, via a canonical seleninic–peroxyseleninic acid cycles, which proceeds in line with Scheme 9. One of the hydroxyl group of 1,2 diol is then oxidized by peroxyseleninic acid to the corresponding α‐hydroxy ketones/aldehydes, that undergo an organoselenium catalyzed BV reaction according to Scheme 18. The ester produced by the BV reaction lastly undergoes hydrolysis to the final product. For the intermediate step, precedents exist in the tert‐butyl hydroperoxide oxidation of secondary alcohols to ketones with diselenides as catalysts. However, to the best of our knowledge, the use of hydrogen peroxide as sacrificial oxidant in the reaction has never been tested.

### 3.2 Heteroatom (X) Oxidations (X=S, Se, Br, N)

#### 3.2.1 Sulfoxidations and Autocatalytic Selenoxidations

Beside C=C and C=O bonds, organoselenides have also been employed for the catalytic activation of hydrogen peroxide to oxidize heteroatoms such as chalcogens (S, Se), halogens (Br) and nitrogen (N). The capacity of organoselenides to act as oxygen‐transfer agents for the practical sulfoxidation of organosulfides was probably the first example reported within this field. Indeed, Reich and coworkers proposed that the seleninic acid produced by the overoxidation of the selenenic acid moiety released via a selenoxide elimination could act as a catalyst for selenide oxidation to selenoxide. (Scheme 2) The oxidation of sulfides can be considered the natural extension of this reaction. In 1978, Reich and Chow reported the oxidation of di‐n‐butyl‐sulfide to the corresponding sulfone with 1 mol % of catalyst **c**.^[202]^ The oxidation to sulfones occurred readily at room temperature, leading in three hours to quantitative yields in sulfone when excess hydrogen peroxide (2 eq.) was used. However, the same authors also reported the possibility of reaching high excesses in sulfoxide working under controlled conditions.^[202]^ Later on, this possibility was confirmed and further explored by Syper^[211]^ and Mlochowski.^[252]^

Particularly, in 1996, Mlochowksi reported the clean oxidation of thioanisole to the corresponding sulfoxide, with only traces of the overoxidized sulfone as a byproduct, when ebselen (**w**) was employed as the oxygen‐transfer catalyst. The authors additionally tested an oxidized (**x**) ebselen‐based catalysts, and ebselen diselenide (w*) which provided mostly good catalytic performance in hydrogen peroxide activation towards substituted thioanisoles. Particularly, the commercially available ebselen allowed the complete conversion of thioanisole and the electronrich *p*‐CH_3_ thioanisole. Conversely, EWGs on the aromatic ring of thioanisole slowed down the reaction, leading to a reduced yield in sulfoxide alone with all three catalysts. In all cases, by working with only a slight excess of hydrogen peroxide (1.2 equivalents), overoxidation of the sulfoxide product to the sulfone functional group was mostly avoided, and only 0.1–2 % of the overoxidized product was obtained for the more reactive substrates.^[252]^

The same authors also reported the immobilized version of catalysts **w** in 2008, which showed good performance in sulfides oxidation to sulfoxides.^[253]^ Employing this system, they further highlighted how the sulfoxide product alone can be obtained by working with moderate excesses of hydrogen peroxide (1.2 equivalents). Sulfoxide oxidation to sulfone occurs only with larger excess of hydrogen peroxide (2.5 equivalents). Interestingly, diphenyl sulfoxide was found to be resistant against the overoxidation to sulfone with ebselen catalyst **w** and even more sowith its immobilized version.^[253]^ Thus, in general, while organoselenium catalysts are quite capable of oxidizing sulfides up to the corresponding sulfones, the sulfonization process can generally be avoided, as pioneeringly proposed by Reich and Chow in 1978.

The catalyst **r** introduced by Back and coworker in 2012 (Scheme 13) was also found to be an active catalyst in sulfoxidation reactions, with as little as 1 eq. of hydrogen peroxide. Under the conditions of Scheme 13, only minor overoxidation to the sulfone product was observed. Particularly, the authors found that the use of a 9 : 1 mixture of dichloromethane and methanol as well as addition of magnesium sulfate significantly reduced overoxidation to the sulfone, As discussed for epoxidations, the active oxidant was hypothesized to be either a peroxyseleninic acid or an hydroxy perhydroxy selenane formed *in situ* via reaction with hydrogen peroxide.24

**Scheme 24:**
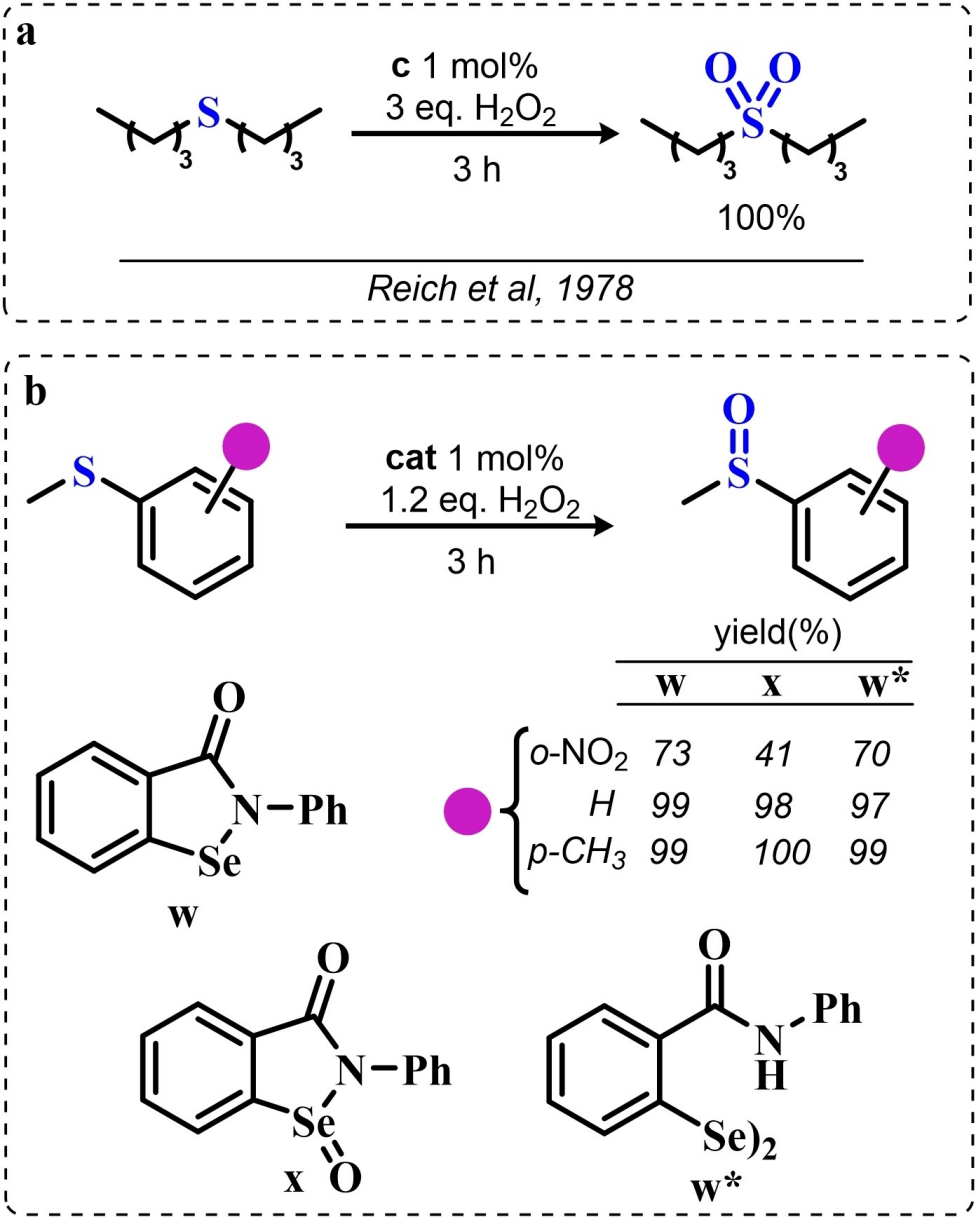
Organoselenium catalyzed oxidation of sulfides to sulfoxide and sulfone by hydrogen peroxide. **a** Reich oxidation of sulfide to sulfone; **b** Mlochowski oxidation of sulfide to sulfoxide with ebselen‐based catalysts.

**Scheme 25:**
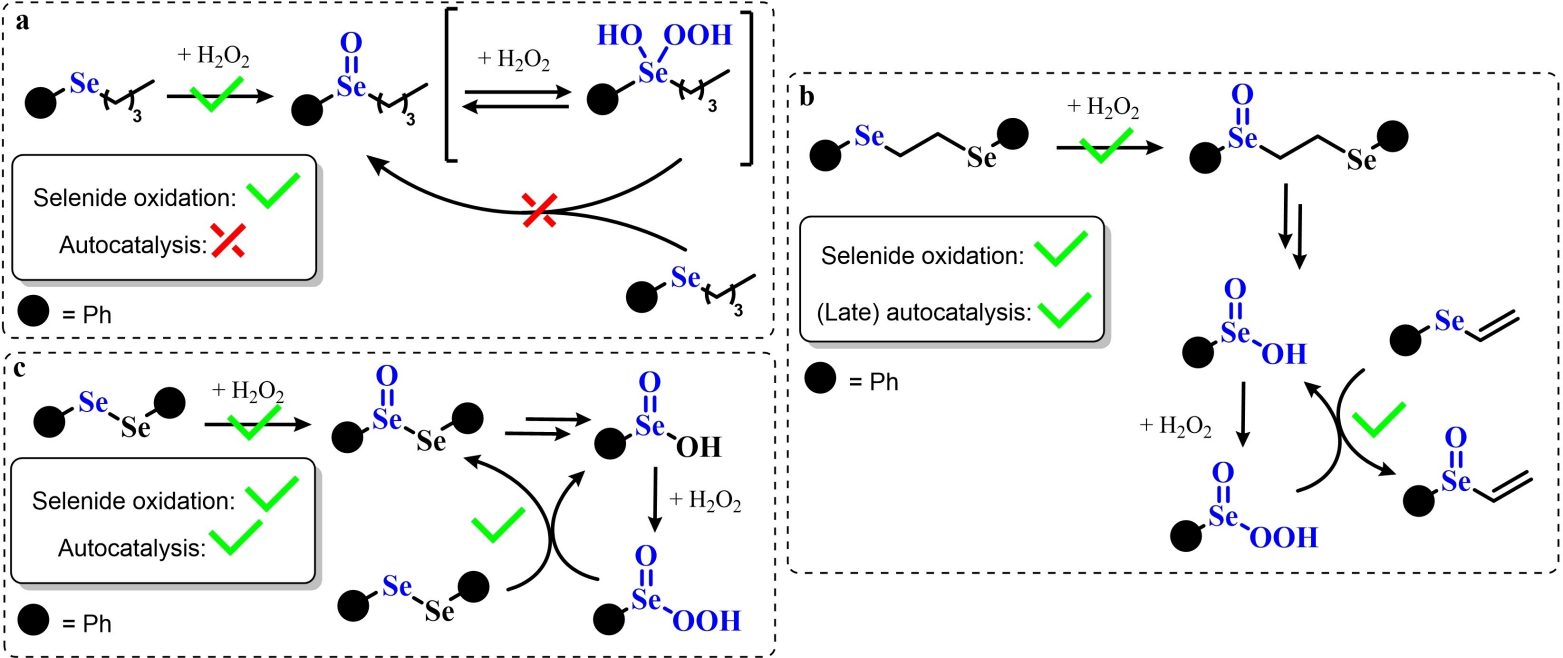
Oxidation mechanisms and eventual autocatalysis of **a** monoselenides; **b** ethyl diselenides; **c** diselenides.

While sulfides were the mostly investigated chalcogen‐based substrates, following the preliminary clairvoyant ideas of Reich and coworkers in 1975 about the autocatalytic oxidation of selenides, *selenium* oxidation by organoselenides has remained somewhat understudied. Indeed, the pioneering mechanistic ideas proposed in the mid‐1970s consolidated despite being supported only by moderate evidence. In 2016, an in‐depth study by Ribaudo and coworkers provided a systematic perspective on the topic, describing hydrogen peroxide oxidation of different classes of organoselenides, assessing *inter alia* the possibility of autocatalysis during Se oxidation (Scheme 25). Four different archetypal selenides, spanning mono and diselenides, were investigated via a combined experimental (NMR and MS) and computational (DFT) approach (Scheme 25). Most importantly, from the kinetic profiles obtained by NMR spectroscopy, no evidence of autocatalysis was observed for n‐butyl phenyl monoselenide (Scheme 25a). Similar results were obtained for bis(phenylselanyl)methane diselenide, in which the two chalcogen nuclei are separated by a methylene bridge. By exploring the reaction mechanism *in silico*, it was observed that even if the selenoxide initially produced by the oxidation of the monoselenides further reacts with hydrogen peroxide to produce a hydroxy perhydroxy selenane with an activation energy comparable to the direct oxidation of the chalcogen nucleus, this species is not a better oxidant than hydrogen peroxide when the organoselenide is the nucleophilic partner in the reaction. Since the selenoxides in Scheme 25a did not undergo any elimination, and thus did not trigger the formation of seleninic acid, only hydrogen peroxide could slowly oxidize the chalcogen nucleus. An analogous discussion holds true for the diselenide separated by a methylene bridge.

Conversely, for bis(phenylselanyl)ethane, in which an ethylene bridge separates the two chalcogens, a different kinetic profile was observed, and thus a different mechanism was proposed (Scheme 25b). Indeed, the relatively slow formation of the selenoxide triggers an elimination reaction, thus leading to the *in‐situ* formation of a phenyl seleninic acid which can further activate hydrogen peroxide. However, this occurred only when most of the initial selenide was oxidized to selenoxide (>70 % conversion). Thus, the released seleninic acid acted mostly as a catalyst for the oxidation of the allyl selenide generated by the selenoxide elimination, leading to autocatalysis features only in the later stages of the reaction.

A third scenario was observed for the oxidation of diphenyl diselenide. For the oxidation of this compound with 3.0 eq. of hydrogen peroxide, after an initial lag phase, the oxidation showed evidence of autocatalysis, which was also in this case rationalized in the light of the formation of phenyl seleninic acid acting as a catalyst in the diselenide oxidation. Most importantly, at the end of the reaction, only the Se(IV) phenyl seleninic acid was observed in solution (as identified on the basis of its chemical shift, i. e., 1169 ppm), and no Se(VI) phenyl selenonic acid was detected. Conversely, in the 2020s, Back and Tanini observed that by treatment with a larger excess of hydrogen peroxide (4.0 eq.), diphenyl diselenide is further oxidized to a selenonium selenonate salt. (Scheme 15) Thus, while Se(VI) species can be formed in large excess of hydrogen peroxide, Ribaudo and coworkers observed that an autocatalytic behavior for selenides oxidation can occur also in their absence, i. e., when only the Se(IV) phenyl seleninic acid is generated *in situ*.

#### 3.2.2 Haloperoxidations

Phenyl seleninic acids dominated the field of organoselenium catalyzed oxygen‐transfer reactions. Conversely, selenoxide‐based catalysts control the field of haloperoxidations. Detty and coworkers have pioneered the field of organochalcogen based catalysts in the activation of halides for halogenation reactions in the 90s.[^254^, ^255^, ^256^, ^257^] Oxidative halogenations are an important class of reactions in which hydrogen peroxide oxidizes halides to activated electrophilic halogenides (i. e., OX^−^ or, in general, “X^+^” species, with X=Cl, Br, I) that can carry out the halogenation of nucleophilic organic substrates (e. g., alkenes). While the oxidation of halides by hydrogen peroxide is well favored thermodynamically, catalysts are required for the reaction to take place in reasonable times.

While tellurides and telluroxides have been first investigated as catalysts, in virtue of their higher reactivity when compared to analogous selenides,[^254^, ^255^, ^256^] highly competent organoselenium‐based systems were reported before the end of the century.^[257]^

**Scheme 26:**
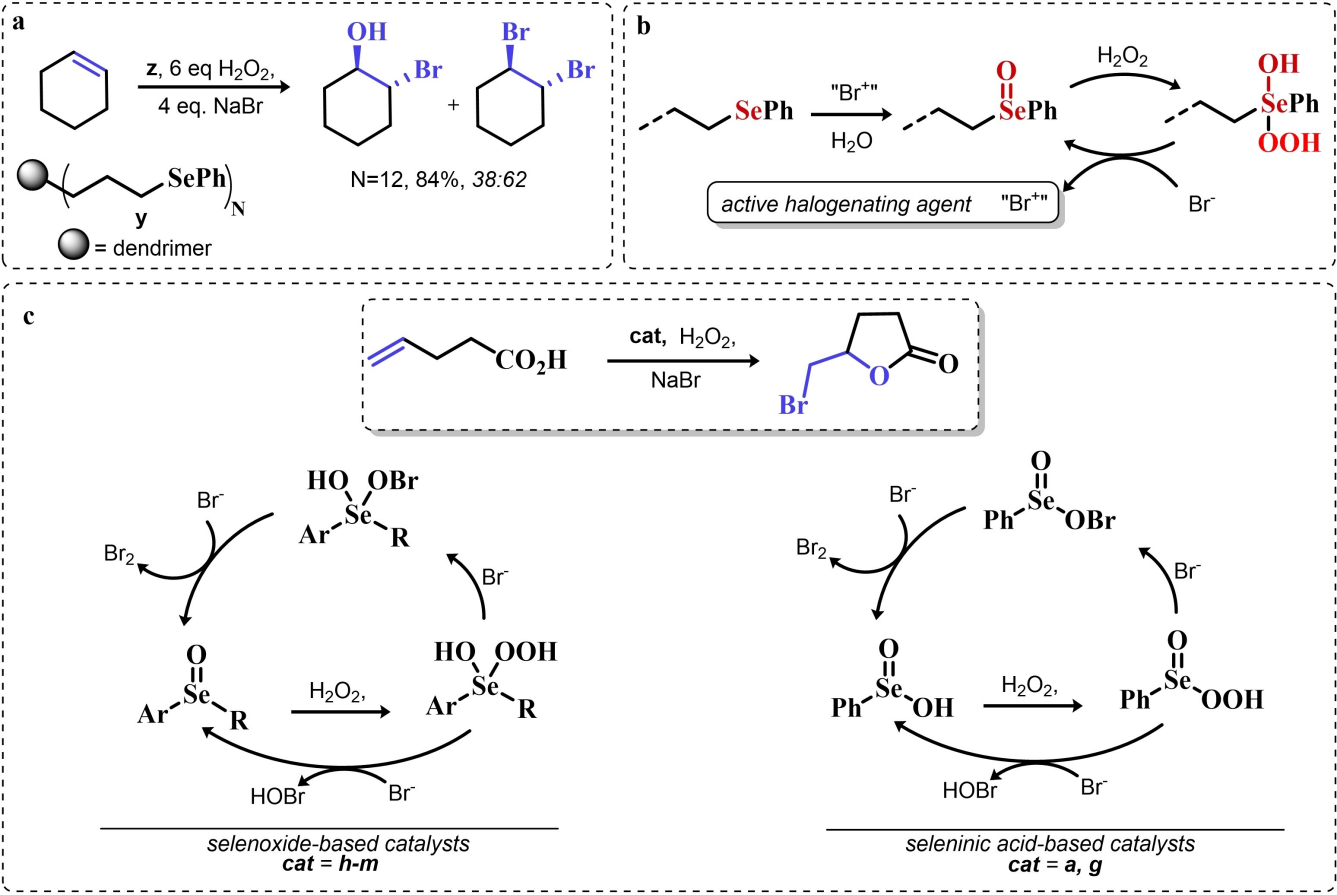
Organoselenium‐catalyzed oxidative halogenations by Detty and coworkers. **a** First reported halogenation of cyclohexene catalyzed by poly selenide dendrimer **y**; **b** Proposed reaction mechanism for the selenoxide‐catalyzed oxidation of bromide to active bromonium species. **c** organoselenium‐catalyzed bromolactonization of enoic acids, and proposed reaction mechanisms for selenoxide‐ and seleninic acid‐catalyzed processes.

Particularly, in 1999 Detty reported the development of a dendrimeric catalyst based on multiple (N=1–12) phenyl selenide units capable of activating bromide with hydrogen peroxide, to perform the bromination of cyclohexene (Scheme 26a). Importantly, the catalysts displayed a significant “dendrimeric effect”, i. e., the activity of multi phenyl selenide units was higher than what might have been expected based on purely statistical arguments. The largest system developed (N=12) could convert cyclohexene into the corresponding brominated products (*trans*‐1,2‐dibromocyclohexane and *trans*‐2‐bromocyclohexanol (Scheme 26a) in three hours, with an overall yield of 84 %. In a follow‐up study, the same authors shed light on the dendrimeric effect, and, by means of solid kinetic evidence, they proposed a reasonable reaction mechanism for organoselenium‐catalyzed oxidative bromination reactions (Scheme 26b). Particularly, the authors concluded that the catalytic activity of catalyst **y** is not related to the seleninic acid which might be released by a selenoxide elimination reaction. Most strikingly, it was also observed that the selenoxide moiety is not the active oxidant, since no reaction occurred by treatment of cyclohexene with bromide and the selenoxide form of catalyst **7**, in the absence of hydrogen peroxide. This led the author to the conclusion that the selenoxide moiety must first undergo further reactivity with hydrogen peroxide to subsequently react with the bromide anion.^[217]^ The formation of a hydroxy perhydroxy selenane is proposed, which lastly oxidizes bromide to a formal bromonium cation which can react with the organic substrate. To the best of our knowledge, this is the first time that the involvement of hydroxy perhydroxy selenane species in organoselenium catalyzed oxidation of organic substrates is proposed, even if the species was previously hypothesized to be formed in oxidizing conditions.^[258]^ Further studies by the same author further revealed the generality of these catalytic schemes also in epoxidation and Baeyer‐Villiger reactions (see also Scheme 11 and relative discussion.) and, later on, in the GPx‐like mechanism of selenoxides (*vide infra*, Paragraph 3.3)[^219^, ^259^]

These results additionally allowed the rationalization of the dendrimeric effect observed for **y(N=12)**. Indeed, for **y(N=1)** an induction time was observed prior to the conversion of the organic substrate, while no induction time was observed for **y(N=12)**. These results were also considered incompatible with a hydrogen peroxide oxidation of the Se atom, since for both N=1 and N=12, this process was too slow to account for the catalytic potential. Conversely, the authors proposed that the “Br^+^” species produced by the background uncatalyzed reaction of hydrogen peroxide with the bromide anion initially oxidizes the selenide to selenoxide. Once the selenoxide is formed, it can catalytically produce more “Br^+^”, thus increasing the local concentration of oxidant within the dendrimer and explaining the dendrimer effect observed for catalyst **y**.^[217]^

In the early 2000s, Detty and coworkers observed also that both simple selenoxides and phenyl seleninic acid could catalyze oxidative bromination reactions, such as bromolactonizations[^218^, ^260^] (Scheme 26c). Only a moderate substituent effect was observed for both classes of catalyst. Curiously, the electron poor catalysts **g** and **m**, which performed well in epoxidation reactions, appeared to be the worst catalysts among the investigated ones. Conversely, the electron rich catalysts (i. e., with EDG on the phenyl rings) performed as good as (for seleninic acid catalyzed) or even better (for selenoxide catalyzed) than the unsubstituted analogues.

For both seleninic and selenoxide catalyzed reactions, similar mechanisms were proposed. In line with the previous results obtained by the same group (Scheme 26b and relative discussion), for selenoxide‐based catalysts the selenoxide moiety is considered to be the catalyst which activates hydrogen peroxide in the form of a hydroxy perhydroxy selenane.[^217^, ^258^] Similarly, seleninic acids are proposed to activate hydrogen peroxide in the form of a peroxyseleninic acid, in line with the previous evidence available at the time.[^204^, ^211^, ^213^] Two plausible bromide activation pathways are then proposed, i. e., either the peroxidic function of both catalysts directly oxidizes the halogenide to the corresponding hypobromite, or a covalent adduct between the organoselenium catalyst and the halogen is formed. A subsequent S_N_2‐like reaction with the nucleophilic Br^−^ leads to the formation of Br_2_ and regenerates the catalyst. The second path was deemed more likely to be relevant for phenyl seleninic acids as catalysts, while no preference for one of the two pathways was hypothesized for selenoxides. However, for this second class of catalysts, the direct participation of the organoselenium bromine species as brominating agent was deemed unlikely on the basis of the similar regioselectivity between the uncatalyzed and catalyzed reactions. A deeper mechanistic investigation of this class of reactions, which goes beyond these pioneering studies by Detty and coworkers, is currently missing.

PhSeCl was also observed to behave as a catalyst for oxidative chlorination reactions, but only preliminary results were reported in 2008, with poor yields in chlorinated products.^[261]^ Conversely, in 2008 and 2012, Detty and coworkers showed that good oxidative halogenation performance can be obtained in aqueous environment or under on water conditions using xerogel sequestered selenoxides,^[262]^ or soluble diselenides, respectively.^[263]^ Particularly, Detty and Braga showed that imidazolium containing diselenides, which are completely soluble in water in catalytic conditions, can act as catalysts for bromolactonization reactions (Scheme 26c), even if partial hydrolysis of the lactone complicates the protocol. Dioxane as a co‐solvent significantly improved the yield in brominated product. Additionally, in 2015, by an XPS analysis on xerogel absorbed diselenides and monoselenides, the presence of the Se(IV) in both diselenides‐based and selenide‐based catalysts was assessed, which is compatible with the catalytic role of seleninic acids and selenoxides, respectively.^[264]^

#### 3.2.3 N‐Oxidations

A third important, but relatively underexplored, class of heteroatoms oxidations in which organoselenides can act as catalysts is the oxidation of amines. When compared against other heteroatom oxidations (e. g., sulfoxidations), organoselenides were tested somewhat late as oxygen‐transfer agents for the effective oxidation of the nitrogen atom. Indeed, even the inorganic SeO_2_, that pre‐dates organoselenides as a catalytic oxidant by decades, was employed for the oxidation of aromatic amine only in 2005, by Ruck‐Braun and Priewisch.^[265]^ In their pioneering work, the authors reported the SeO_2_ catalyzed oxidation of aniline to nitrosobenzene, with nitrobenzene and azoxybenzene as byproducts. While organoselenides, especially the phenyl seleninic anhydride, were tested in the 70s and 80s as stoichiometric oxidants for primary and secondary amines to a variety of products[^266^, ^267^] (i. e., cyano‐derivates, or carbonyl compounds), to the best of our knowledge no catalytic version of the same reaction has been reported so far.

**Scheme 27:**
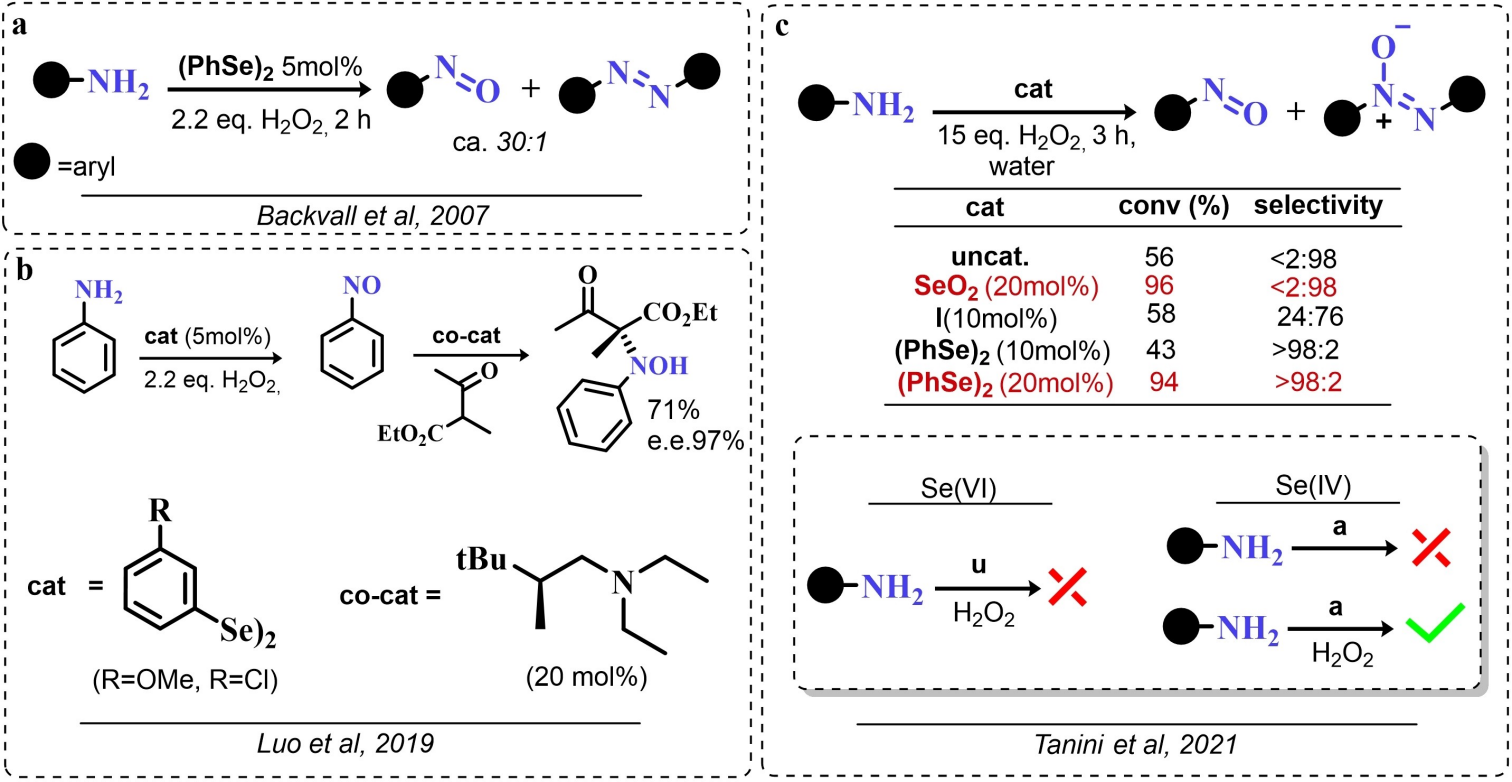
Organoselenium catalyzed arylamine oxidation. **a** pioneering study with diphenyl diselenide; **b** nitrosobenzene in‐situ generation for nitroso aldol reaction; **c** arylamines oxidation to nitroderivatives.

Conversely, the oxidation of arylamine was the subject of a couple of interesting studies in the past twenty years. The first organoselenium‐catalyzed oxidation of aromatic amine was reported in 2007 by Backvall and coworkers (Scheme 27).^[268]^ The authors built on the previous pioneering work of Barton and coworkers, who reported the stoichiometric use of phenylseleninic anhydride to oxidize aromatic hydroxylamine to nitroso derivative.^[199]^ Backvall observed that hydroxylamine as starting material was not required, since this intermediate can also be produced *in situ* by the organoselenium‐catalyzed oxidation of aniline by hydrogen peroxide, which leads in the end to the corresponding nitrosobenzene. Importantly, the authors showed that both 5 mol % diphenyl diselenide and the phenyl seleninic anhydride (which can release two equivalents of the corresponding seleninic acid) provide catalytic performance analogous to 10 mol % benzene seleninic acid. In all three cases, quantitative conversion was achieved at room temperature in less than 2 h, with high selectivity for the formation of nitrosobenzene over azo‐ and azoxybenzene. The participation of phenyl seleninic acid as the direct oxygen‐transfer agent was excluded, since its use as a stoichiometric oxidant produced only minor conversion of aniline. Thus, a conventional seleninic–peroxyseleninic mechanistic scheme was postulated.^[268]^

The same reaction was expanded by Luo and coworkers in 2019,^[269]^ who employed the organoselenium‐catalyzed oxidation of arylamine to produce *in situ* substituted nitroso arene and to trigger a nitroso aldol reaction with a carbonylic partner (Scheme 27b).[^270^, ^271^] Importantly, the authors tested variously substituted phenyl seleninic acids, showing that electron poor seleninic acids behave as better catalysts than electron rich catalysts. Importantly, catalyst **g** was somewhat outperformed by 3‐chloro phenyl seleninic acid. By use of a chiral cocatalyst, good yields and excellent enantiomeric excess in the aldol product could be obtained.^[269]^

While nitrosoarenes were the target products in these pioneering investigations, Tanini and coworkers observed in 2021 that by working in excess of hydrogen peroxide, oxidation of substituted arylamines to the nitroarenes can be achieved under on water conditions(Scheme 27c).^[272]^ Alkyl di‐ and monoselenides proved to be poor catalysts for the reaction, leading to a moderate conversion of aniline and to a poor selectivity toward the formation of nitrobenzene. Conversely, diphenyl diselenide and phenyl seleninic acids proved to be good catalysts leading to almost quantitative conversion of the starting material to the nitroderivatives in ca. 3 h at room temperature. Interestingly, under the same on water conditions, the inorganic SeO_2_ was observed to catalyze the conversion of aniline with similar yields to phenyl seleninic acid, but with a totally oppose selectivity, i. e., only traces of nitrobenzene were observed, while >98 % of azoxybenzene was identified. Most importantly, the authors performed a detailed series of control experiments to identify the active oxidation state in the organoselenium‐mediated process. They showed, *inter alia*, that while benzene selenonic (catalyst **u**) acid is identified in the water after the reaction took place to completeness, this Se(VI) species is not an active catalyst for the reaction. Conversely, after being reduced back to the analogous Se(IV) benzene seleninic acid (catalyst **a**), addition of hydrogen peroxide triggers the catalytic process. These results led the authors to describe the nature of organoselenium‐catalyzed processes as polyhedral, since, despite the strong analogies between the oxygen‐transfer mechanisms, different oxidation states of selenium might be active or totally inactive in various organocatalyzed processes in different environments,^[272]^ as exemplified by the comparison between arylamine oxidation, in which Se(VI) is inactive, and olefine epoxidation, in which Se(VI) is currently recognized as a better oxidant (Scheme 15). Recently, the privileged role of Se(IV) in arylamine oxidation was further corroborated by some of us in a thorough computational study of the reaction mechanisms, highlight the superior properties of seleninic acid (catalyst **a**) in hydrogen peroxide activation for aniline oxidation.^[273]^

### 3.3 Glutathione Peroxidase‐Like Catalysis

**Figure 7:**
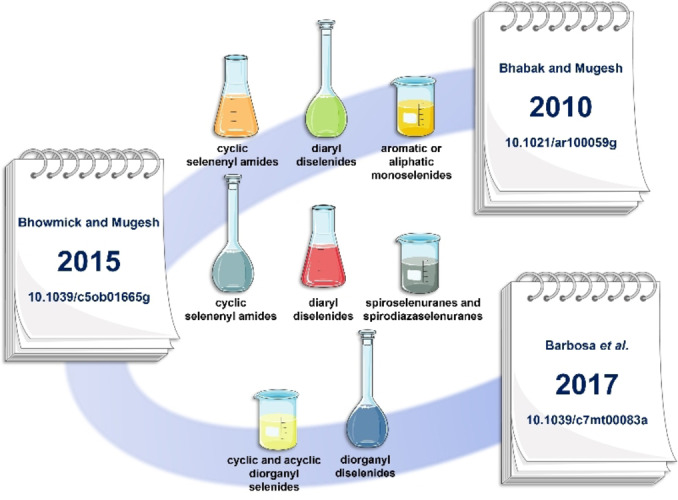
Various classification of organoselenides as GPx mimics.

As mentioned above (Paragraph 2), members of the GPx family regulate the peroxide tone in the cell. Given the detailed knowledge about the reaction mechanism of GPx and its deep involvement in the human antioxidant defenses, organic and medicinal chemists have been tempted by the idea of capturing the enzyme activity and transferring it to a small, drug‐like molecule which can sustain the same, or a similar, catalytic cycle. Organoselenides had, obviously, a privileged role in this field given the importance of Sec in GPx catalysis and several reviews can be consulted on the topic, giving detailed perspectives of their structure, mechanism and medicinal potential.[^12^, ^14^, ^274^, ^275^] In the following, only a couple of seminal mechanistic topics will be covered, to highlight the reactivity of organoselenides as catalysts for the oxidation of thiols to disulfides. Possible applications in the biological environment, will be covered in detail later on (*vide infra*, Paragraph 4). In the past, GPx mimics have been variously categorized in the basis of their chemical structure, cyclic or acyclic, and on the basis of the organoselenium functional group, monoselenides, diselenides, or selenyl amides (Figure 7).[^276^, ^277^, ^278^]

On the basis of their mechanisms, three different reactive schemes can be described, which are applicable to the three classes proposed by Mugesh in 2010,^[278]^ also lately reviewed by Toppo and Orian.^[274]^

The first GPx mimics to reach widespread recognition is the widely studied small molecule named ebselen, or 2‐phenyl‐1,2‐benzisoselenazol‐3(2H)‐one, (also labelled as catalyst **w** in Scheme 24) which was first synthesized in 1924 (before the discovery of the nutritional importance of selenium!) and only later re‐discovered as an antioxidant agent in 1984, by the independent contributions of research groups of Sies and Wendel.[^279^, ^280^, ^281^] Mechanistic studies on the reaction of ebselen with peroxides and thiols started as early as in the late 80s,^[282]^ and more than thirty years later, the all‐around description of its reactivity and of the reactivity of other GPx mimics is still an active area of research with contributions coming from both experimentalists and theoreticians.[^281^, ^283^, ^284^] The reactivity of organoselenides as GPx‐mimics has been extensively investigated and reviewed,[^12^, ^274^, ^276^] thus in the following we only highlight the origin of the field and a couple of fundamental points about our current mechanistic understanding of three different organoselenium species, namely ebselen, diphenyl diselenide and monoselenides. The reader is referred to specialistic reviews about the design of GPx‐mimics for a deeper understanding of the factors controlling their reactivity.

**Scheme 28:**
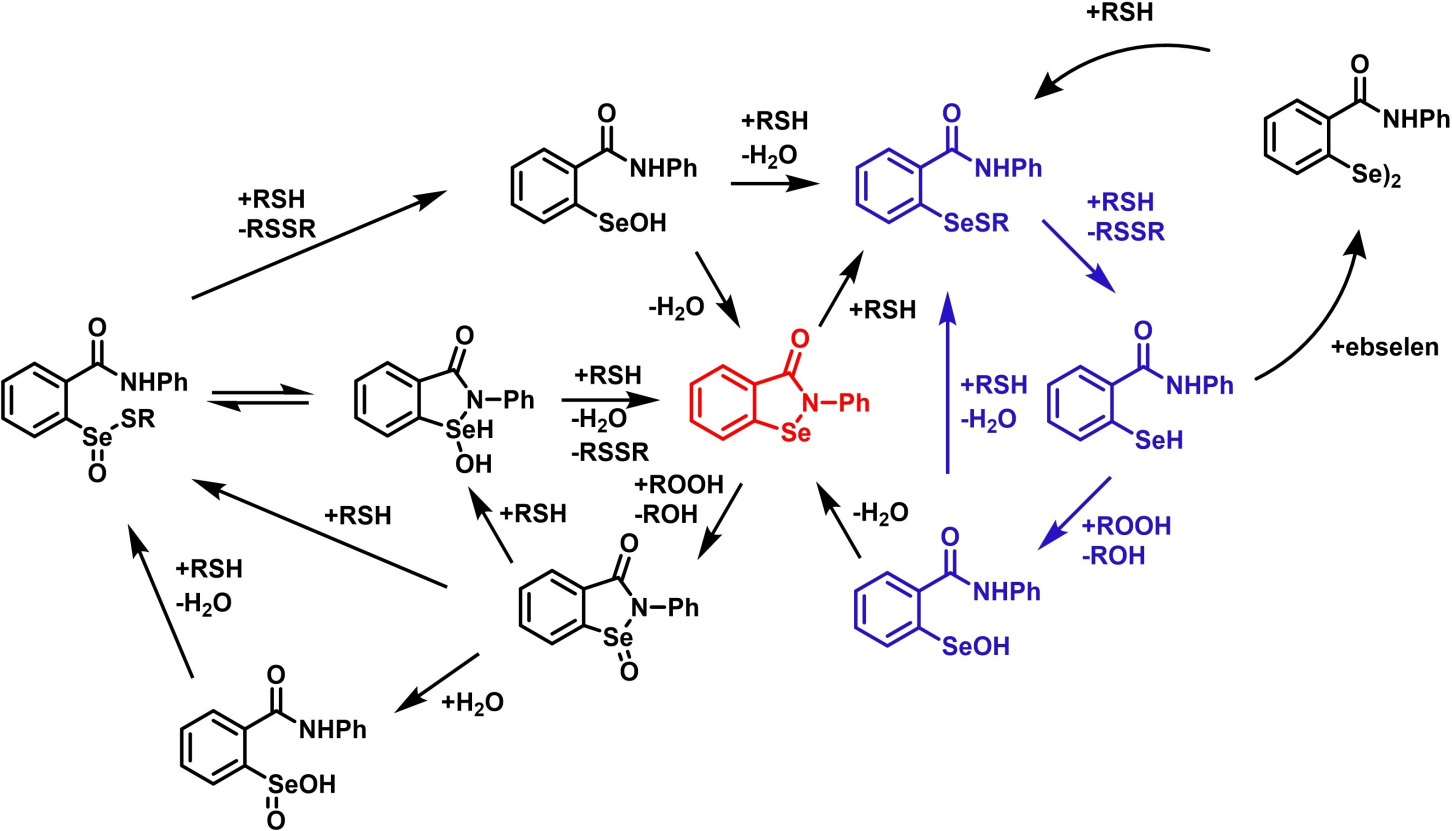
Comprehensive picture of GPx‐like chemistry of ebselen (in red). The GPx‐like catalytic cycle sharing the fundamental reaction intermediates present also in the conventional GPx enzymatic catalysis (Figure 3) is highlighted in blue.

Ebselen and diphenyl diselenide follow catalytic cycles which are mostly reminiscent of the GPx enzyme.^[283]^ While their structure is highly simplified when compared to the enzyme, they catalyze hydrogen peroxide reduction and thiol oxidation via the same reaction intermediates, namely, a selenol, a selenenic acid, and a selenyl sulfide species (Scheme 28).

The basic potential mechanisms of the thiol‐peroxidase like activity of ebselen have been studied *in vitro* and *in silico*. However, only few studies utilized a medium that can be comparable with the physiological and aqueous environment of the living cells.[^285^, ^286^, ^287^] To better understand the molecular mechanism of GPx‐like activity of ebselen, in 1992 Engman's group synthesized ebselen intermediates of the catalytic cycle.^[288]^ The kinetics of the reaction with hydrogen peroxide were investigated, and ebselen selenol (2.8 mM^−1^ min^−1^) showed higher activity than ebselen (0.29 mM^−1^ min^−1^), ebselen‐GSH selenosulfide (≤0.01 mM^−1^ min^−1^), and ebselen diselenide (0.32 mM^−1^ min^−1^). Additional assays determined relative ebselen selenol and diselenide levels under typical peroxidase assay conditions, suggesting that, among all possible intermediates, the selenol is the predominant species responsible for the peroxide reduction in the GPx‐like activity. It is plausible to conclude that endogenous dithiols (for instance, reduced lipoate and proteins containing vicinal thiols) should be better substrate to activate (or to form the selenol intermediate of) ebselen and other diselenides.[^283^, ^284^, ^289^]

While other intermediates are also possible, such as an ebselen diselenide or an overoxidized seleninic acid, under physiological conditions these species are unlikely to be produced. Particularly, while the oxidation of the diselenide form of ebselen might in principle occur, thus making the diselenide an active species in peroxide reduction, this path is quite slow and unlikely to occur under biological conditions.[^282^, ^284^, ^289^, ^290^, ^291^] Thus, the selenenic acid of ebselen more reasonably reacts with thiols to produce the conventional selenyl sulfide intermediate (*blue path* in Scheme 28). Importantly, the formation of this selenyl sulfide intermediate has been over and over recognized as a “trap” for the catalysts and as the reason for the relatively low catalytic competence of ebselen. Indeed, while in GPx the second nucleophilic thiol attack occurs on the S end of the selenyl sulfide, in the free ebselen the Se nucleus is unprotected, and the enhanced electrophilicity of the heavy chalcogen preferentially directs the nucleophilic attack towards itself. This thiol scrambling reaction prevents the regeneration of the selenol functional group, and thus further peroxide reduction.[^292^, ^293^] Expectedly, the development of strategies to hamper this thiol scrambling reactions has been an active area of research, with the exploitation of weak O/N intramolecular interaction being recognized as a useful way of influence the reactivity of selenyl sulfides.[^12^, ^292^, ^294^]

**Scheme 29:**
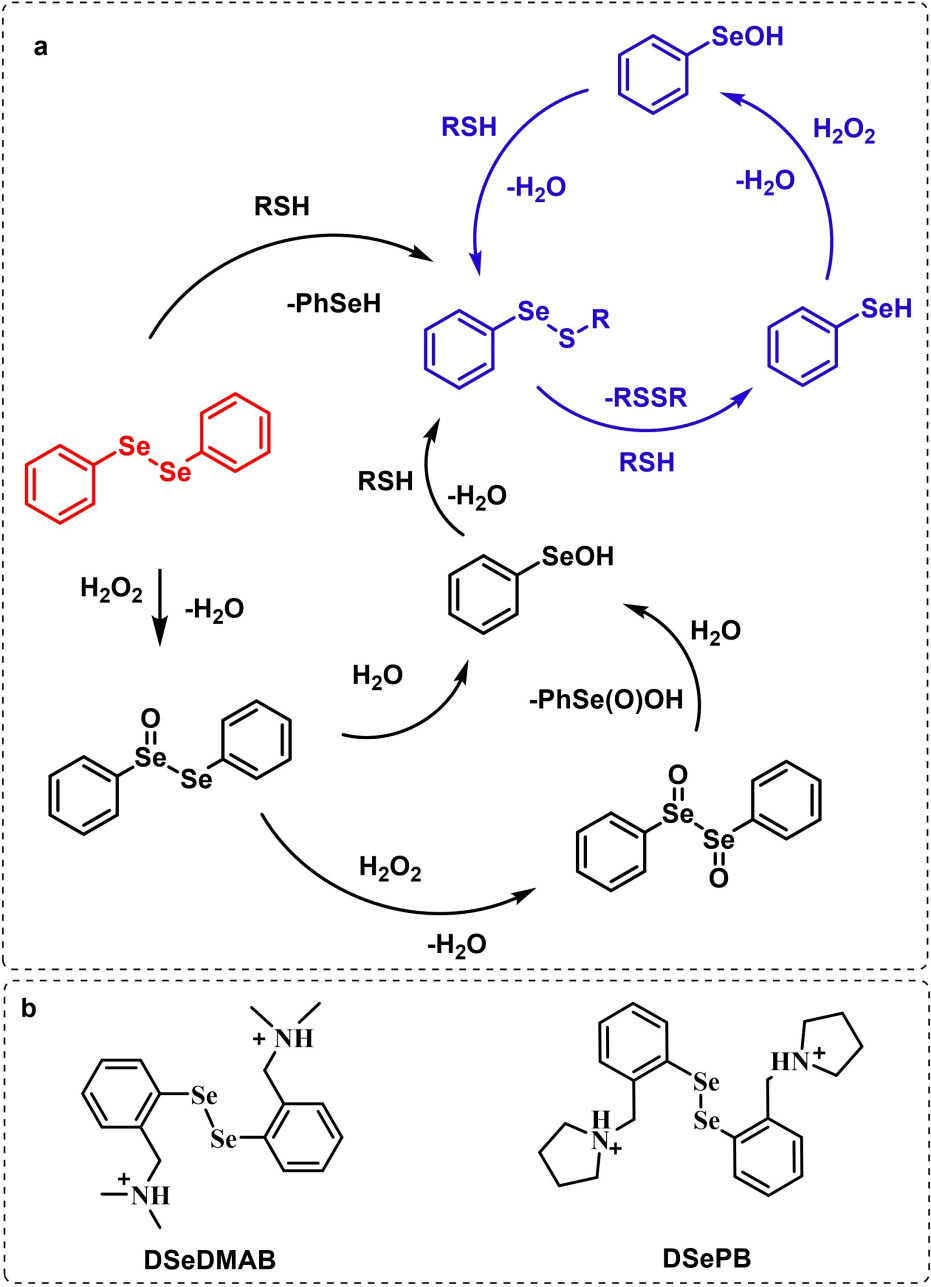
**(a)** Comprehensive picture of GPx‐like chemistry of diphenyl diselenide (in red). The GPx‐like catalytic cycle sharing the fundamental reaction intermediates present also in the conventional GPx enzymatic catalysis (Figure 3) is highlighted in blue. **(b)** Diselenides with enhanced GPx‐like activity designed by Wilson and coworkers.

Diphenyl diselenide was also discovered quite early for its weak GPx‐like activity, with the first reports dating back to the late 1989,[^285^, ^295^] and our current mechanistic understanding of this class of compound closely matches to the one of ebselen and the native GPx, with the only difference being the initial cleavage of the Se−Se bond by a thiol. Conversely, Ribaudo *et al*. showed that under oxidant conditions in which thiols are lacking, the diselenide is slowly converted in an autocatalytic manner to seleninic acid^[296]^ (and to some extent to selenonic acid under an even larger excess of hydrogen peroxide, as observed by Back and Tanini).[^237^, ^272^] Once the selenyl sulfide is produced, the catalyst enter the conventional GPx‐like cycle, with a second equivalent of thiol generating a selenol/selenolate moiety that can reduce one equivalent of peroxide (Scheme 29a). Both Wilson and coworkers^[285]^ as well as Iwaoka and Tomoda^[297]^ showed that diselenides with an amino group in ortho to the Se atom are even better catalysts for hydrogen peroxide reduction, due to the capacity of the basic amine to both stabilize the nucleophilic selenolate as well as to prevent the unwanted thiol scrambling mechanism in the selenyl sulfide function. Particularly, (2,2′‐diseleno bis[[(*N*,*N*‐dimethylamino)methyl]benzene] bis(hydrochloride salt) (DSeDMAB) and 2,2′‐diseleno bis[(pyrrolidin‐1‐ylmethyl)benzene] bis(hydrochloride salt) (DSePB)), were found to have enhanced catalytic activity when compared against diphenyl diselenide^[285]^ (Scheme 29b).

**Scheme 30:**
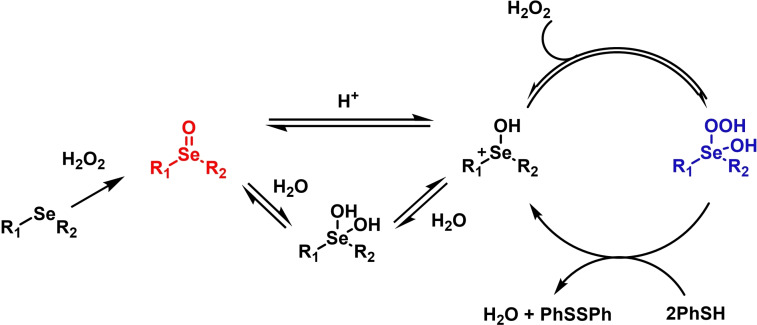
Comprehensive picture of GPx‐like chemistry of selenoxides (in red) produced by the preliminar oxidation of monoselenides. The proposed active oxidant, hydroxy perhydroxy selenane, is highlighted in blue.

Interestingly, also monoselenides can display GPx‐like catalytic activity. This result is remarkable especially for selenides incapable of undergoing a beta‐elimination reaction, because in this case formation of selenol or seleninic acid is prevented. Since selenoxides have been long known for their oxidizing capacity, they have been proposed in the past as active oxidizing species against thiols, once they are formed via oxidation of the chalcogen nucleus by hydrogen peroxide.[^298^, ^299^, ^300^] However, Braga and Detty showed that the initial oxidation of the monoselenide to selenoxides occurs too slowly to be compatible with the catalytic activity reported for these systems. Thus, in analogy with previous studies on haloperoxidations and Baeyer‐Villiger reactions, (*vide supra*, Scheme 11 and 27) Detty and Braga proposed the monoselenide to act as a pre‐catalysts, which is slowly oxidized to a selenoxides species acting as the true catalyst in the reaction. Particularly, the presence of an hydroxy peroxy selenane species was proposed also for the GPx‐like mechanism of these system,^[259]^ in analogy with the previous proposals for other reactions catalyzed by the same pre‐catalysts[^217^, ^218^, ^219^] (Scheme 30). To the best of our knowledge, a definitive experimental fingerprint of hydroxy perhydroxy selenane is still lacking, and their proposal is mostly based on kinetic evidence.

While the chemical behavior of organoselenides in thiol peroxidase mechanism is well‐assessed, biological studies are still evolving and we have only scarces evidence to understand their behavior under biological conditions. Thus, in the following, principles of “organoselenium pharmacology” will be delineated, to complement the discussion of GPx‐mimics only briefly highlighted above.

## 4 Selenium in Pharmacology: From GPx‐Mimics to Thiol‐Modifying agents

An uncountable number of synthetic organoselenium compounds (aliphatic and aromatic diorganyl(di)selenides and selenylamides) have been synthesized and studied in biological *in vitro* and *in vivo* models. Several of them have been reported to have pharmacological properties, such as antioxidant, anti‐inflammatory, neuroprotective, antinociceptive, anxiolytic, antidepressant‐like, antitumoral, gastroprotective, and antiviral, among others (for comprehensive reviews see[^13^, ^14^, ^275^, ^301^, ^302^, ^303^, ^304^, ^305^]). However, as discussed in more detail below, there is a lack of studies on their metabolism either *in vitro* or *in vivo*. Indeed, for *in vivo* studies, little to nothing is known about the concentrations of the active compounds in the alleged target tissues or organs. In these studies, indirect pharmacological approaches are frequently utilized, often leading to unsupported conclusions. An additional serious problem is the precarious knowledge about the molecular targets of organic selenocompounds both *in vitro* and *in vivo*. Consequently, very little is known about their mode of action (MoA). In short, the subfields of pharmacology and toxicology of organoselenium compounds have not progressed from the phenomenological approaches of the 20^th^ century to the more mechanistic approaches of the 21^st^ century.

Since only a few studies have explored the molecular pharmacological or biochemical processes involved in the beneficial effects of organoselenium compounds, here we will not review the vast literature on the speculative modes of action of different organoselenium compounds in rodent models (the reader is referred to previous narrative[^14^, ^275^, ^303^] and critical reviews^[274]^ by some of us). Here we will give emphasis only to ebselen (which reached clinical trials in humans) and to some diselenides that react with thiols and can potentially form a selenol intermediate[^285^, ^306^] (for review see[^276^, ^307^]), and can thus imitate the function of selenocysteine in selenoproteins (for a comprehensive and historical review see^[308]^).

Importantly, while selenium compounds are known for their toxicity, some of them can be quite innocuous to mammals. While the toxicity of ebselen and diphenyl diselenide (in red in Scheme 28 and 30) has been reported in rodents, flies and fish,[^14^, ^309^, ^310^, ^311^] no toxic effects were reported for ebselen in human clinical trials studies (ischemic stroke, aneurysmal subarachnoid hemorrhage, acute middle cerebral artery occlusion, type 1 and type 2 diabetes mellitus, and mania or hypomania) with 2–4 weeks of duration.^[312]^

### 4.1 The Glutathione‐Peroxidase‐Like Activity of Organoselenium Compounds

The capacity of synthetic organoselenium compounds to imitate the activity of glutathione peroxidase^[281]^ gave origin to the terminology of glutathione‐ or thiol‐peroxidase mimics in organic synthesis (*vide supra*).^[274]^ The basic idea was to synthesize low‐molecular mass molecules that could resemble, at least partially, the protein active site and catalyze the enzymatic reaction.[^313^, ^314^, ^315^] The interest in the development of enzyme mimetic agents next escalated also in the field of organic selenium chemistry, particularly with the synthesis of the so‐called glutathione peroxidase mimics.[^280^, ^316^, ^317^]

Ebselen (PZ‐51),[^279^, ^318^] and, subsequently, innumerous organoselenium compounds[^288^, ^299^, ^316^, ^319^, ^320^, ^321^, ^322^, ^323^, ^324^] were denoted with this term. However, it is important to highlight that the capacity of some low molecular weight organic selenium compounds (i.e, selenocystine and selenocystamine) to imitate the activity of glutathione peroxidase had been demonstrated some years earlier by Yasuda *et al*. (1980), who did not refer to a peroxidase‐like activity.^[306]^

The limits of most of these studies were the absence of a comparison with the purified glutathione peroxidase. Wilson *et al*. (1989) was the only comparative study reporting on ebselen, diphenyl diselenide and several derivatives, and a purified GPx.^[285]^ The authors also tested the activities in aqueous medium. Furthermore, it is worth to mention here those studies that use the methodology of Iwaoka and Tomoda (1994),^[297]^ which use thiolphenol for the peroxide reduction in methanol. This methodology is very simple and cheap and can be used to perform high throughput screening of numerous organoselenium compounds in a short time. However, the thiol‐peroxidase like activity has to be compared with ebselen and diphenyl diselenide as positive or internal controls, but cannot be straightly extrapolated to the assay used by Wilson *et al*.^[285]^ Anyway, to some extent, the assay of Iwaoka and Tomoda^[297]^ correlates with the methodology using the coupled gluthathione reductase and NAPDH to quantify the rate of GSSG (oxidized glutathione) formed in presence of peroxide, at least for ebselen and diphenyl diselenide. For instance, in the study of Wilson *et al*.,^[285]^ the activity of diphenyl diselenide was two times higher than that of ebselen. The Iwaoka and Tomoda methodology, with methanol or ethanol diselenide, the GPx‐like activity was about 2.5 times greater than ebselen.^[325]^ In the study of Wilson *et al*., the activity of ebselen was set to 1, while that of purified GPx to 10.000.^[285]^ Thus, ebselen resulted four orders of magnitude less effective than the enzyme. The authors reported that DSeDMAB and DSePB (Scheme 29b), were three orders of magnitude less effective than the purified GPx, and the activity of diphenyl diselenide was intermediate between these derivatives and ebselen. Unfortunately, the authors did not calculate the molar concentration of GPx used in the assay. Since each purified enzyme preparation can have a specific activity and the enzyme can lose its activity during storage, it was not possible to use the activity reported by Wilson *et al*. for comparison in subsequent studies. The inclusion of GPx and other purified selenoenzymes as comparative standards for organoselenium compounds might be important to determine whether or not the thiol‐peroxidase like activity is promising for the development of safe therapeutic agents.

In summary, although the thiol‐ or glutathione‐peroxidase like activity of some organoselenium compounds may have a role in their pharmacological effects, there is no direct evidence of their activity in real biological medium (in vitro in cell cultures or *in vivo* at the organismal level). Conversely, the thiol modifier effects of organoselenium compounds (their ability to oxidize endogenous thiols to disulfides) can rather be involved in the pharmacological effects of ebselen and diphenyl diselenide (see Section 4.2).

### 4.2 The Thiol Modifier Effects of Organoselenium Compounds

The molecular shape of ebselen and diselenides is simple and, consequently, may justify the lack of selectivity of these low molecular mass selenium compounds. That is, they react rapidly with an array of thiol‐containing molecules in living cells, like cysteine (Cys) and glutathione (GSH). The literature has also indicated that dithiol‐containing molecules with vicinal −SH usually react more efficiently with organoselenium compounds than monothiols.[^288^, ^326^, ^327^] The adducts formed have new structural and physical‐chemical properties, affecting the reactivity, bioavailability, distribution, excretion, selectivity, and interactions with macromolecular targets.

**Figure 8:**
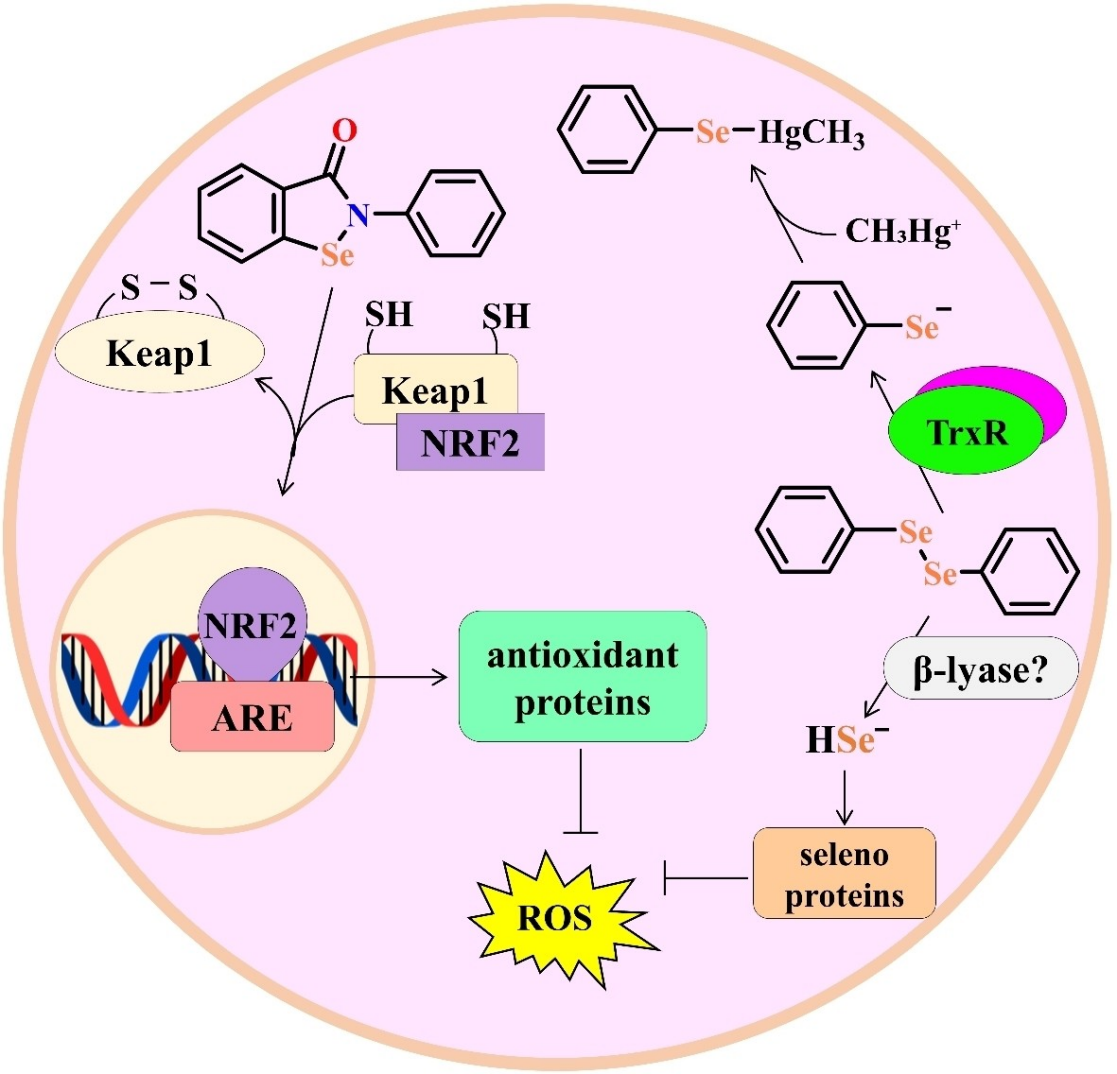
General mechanism of action of ebselen (left) and diphenyl diselenide (right).

The thiol modifier action of organoselenium compounds at non‐toxic concentrations in various mammalian cells, i.e, the non‐specific oxidation of thiol (−SH) moieties, has been demonstrated as an indirect antioxidant mechanism involved in the activation of transcription factors playing a role in the biosynthesis of antioxidant proteins. For instance, ebselen, diphenyl diselenide and its derivatives can oxidize the thiol‐containing Keap1 (which is an inhibitor of the NRF2 ‐ the nuclear factor erythroid 2‐related factor 2). The oxidation of Keap1 releases it from the NRF2 that migrates to the nucleus and activates the expression of antioxidant genes in bovine aortic endothelial and other types of cells.[^276^, ^286^, ^328^, ^329^, ^330^, ^331^, ^332^, ^333^] (Figure 8)

Accordingly, in Schwann cells (RSC96), the cytotoxicity caused by high concentration of glucose (100 mM) was reduced by high concentrations of diphenyl diselenide (10–100 μM).^[334]^ One limitation of this study was the absence of a curve of diselenide at low glucose levels.

In an *in vitro* model of osteoarthritis, diphenyl diselenide activated the NRF2 signaling, which was associated with chondrocytes cytoprotection against IL‐1β. The protection was associated with an increase in the immunocontent of NRF2 but to a decrease in the Keap1 expression.

In rat glomerular mesangial (HBZY‐1) cells, diphenyl diselenide protected the renal cells from the cytotoxicity induced by tert‐butylhydroperoxide and the inflammatory response induced by lipopolysaccharide,^[335]^ which was associated with the activation of the NRF2/Keap1 pathway and the expression of genes coding for antioxidant proteins.

Diphenyl diselenide and some of its derivatives (mainly 2,2′‐diselenyl dibenzoic acid ‐ DSBA) activated the NRF2 in murine fibroblasts and in HepG2 human hepatocarcinoma cells.[^336^, ^337^, ^338^] Similarly, the dimethyldiselenide (CH₃SeSeCH₃) was reported to modulate the NRF2‐associated antioxidant response and to induce phase II glutathione‐S‐transferase (GST) enzyme activity in murine hepatoma (Hepa 1c1c7) cells.[^339^, ^340^] In contrast, Zhang *et al*. (2021) have not observed an activation of the NRF2 pathway after the exposure of primary microglia to diphenyl diselenide.^[341]^

An antioxidant and anti‐inflammatory nanogel of diselenide‐bridged hyaluronic acid was reported to protect RAW 264.7 macrophages and HT29 cells from H₂O₂ or lipopolysaccharide.^[342]^ In RAW 264.7 cells, the protective effects of diselenide‐bridged hyaluronic acid was associated with a translocation of NRF2 from the cytoplasm into the cell nucleus and with a decrease in the levels of inflammatory cytokines.^[342]^

It is important to highlight that the activation of thiol‐containing transcription factors by ebselen and diselenides could be interpreted as a nonspecific oxidation of crucial thiol groups found in thiol‐containing proteins. And when the non‐specific oxidation of thiol groups is excessive, the redox thiol‐disulfide balance can be disrupted, causing unpredictable toxicological effects.^[274]^

### 4.3 Biological Interactions with Electrophilic Species

The antioxidant activity of organoselenium compounds can be related to their capacity of catalyzing the reduction of hydrogen peroxide (H₂O₂), and other organic peroxides. The high reactivity of selenol (R‐SeH) group in cellular environments makes the organoselenium compounds mimics of glutathione peroxidases,^[343]^ as previously described in section 3.3.

There is indirect and limited *in vitro* and *in vivo* evidence that organoselenium compounds form −SeH and HSe^−^ metabolites in rodents. The strong affinity of the nucleophilic selenium (e. g., −SeH and HSe^−^) for soft electrophiles, such as inorganic and organic mercury (E^+^Hg) leads to the formation of species like PhSeHgCH₃, PhSeHgSePh, CH₃HgSeH or HgSe. The evidence for the formation of organic selenium‐mercury intermediates are indirect and based on the capacity of diphenyl diselenide and ebselen of changing the toxicity of CH₃Hg^+^ or Hg^2+^.[^276^, ^344^, ^345^, ^346^, ^347^, ^348^, ^349^, ^350^, ^351^, ^352^, ^353^, ^354^, ^355^] Organomercurials are very toxic, because they can cross cellular membranes, including the blood‐brain barrier, leading to significant damage to the central nervous system.[^276^, ^344^, ^348^, ^356^, ^357^] The formation of species like PhSeHgCH₃ suggests that the existence of highly reactive selenol(ate) intermediates originating from diselenides (reaction: PhSe^−^ + CH₃Hg^+^→PhSeHgCH₃). In fact, the formation of Se−Hg adducts *in vivo* is responsible for the heavy metal detoxification mechanism of naturally‐occurring organoselenium compounds (e. g., selenomethionine, selenocysteine, methylselenocysteine). The formation of either RSeHgCH₃ or RSeHgSeR, besides neutralizing the toxicity of E^+^Hg forms, can accelerate the decomposition of selenium compounds to the inorganic HgSe.[^358^, ^359^, ^360^]

Regarding the behavior of diphenyl diselenide in rodents exposed to CH₃Hg^+^ or Hg^2+^, the species and the temporal relationship between the exposure to diselenide and the E^+^Hg influenced considerably the toxicity of Se‐ and Hg‐containing compounds.[^349^, ^350^, ^351^, ^361^, ^362^, ^363^, ^364^, ^365^] For instance, quite different metabolic pathways between mice and rats were observed. The formation of RSeHgR adducts in mice was accompanied by a decrease in the liver, kidneys, and brain Hg levels. In contrast, in the rats, an increase in the Hg deposition in the brain and liver was reported.[^344^, ^366^] Possibly, this is due to the formation of insoluble particles of mercury selenide (HgSe) upon cleavage of the RSeHgR adduct (RSeHgR→HgSe). In fact, HgSe have been reported in several species.[^367^, ^368^, ^369^] Finally, considering the affinity of Se (and S) for Hg, studies have demonstrated that *N*,*N*′‐imidazole and *N*,*N*′‐benzimidazole‐based thiones/selones have potential detoxification activity against organomercurials, acting as mimics of the bacterial organomercurial lyases. In this process, the proteolytic cleavage of Hg−C bond occurs, and the selones react faster than the sulfur analogs; inert species, such as HgS, HgSe, and HgSSe, are formed.[^370^, ^371^]

### 4.4 The Limited Knowledge on the Metabolism of Organoselenium Compounds

Studies demonstrating that the metabolism of organoselenium compounds leads to the selenide formation (H₂Se, HSe^−^, or Se^2−^) are rare. The most important studies were performed by Schwarz, Foltz, and Fredga between 1950 and 1975, with the aim of determining those organoselenium compounds with the ability of protecting rats from the fulminant liver necrosis induced by a diet deficient in selenium, vitamin E and sulfur amino acids.[^370^, ^372^, ^373^] They pointed out that, similarly to Na₂SeO₃ several mono and diselenides could be source of dietary selenium. Posteriorly, studies about the Se metabolism in the mammalian body demonstrated that cationic Se (Se(IV) and Se(VI)) and organoselenium compounds have to be metabolized to HSe^−^ to be incorporated into the selenoproteins as selenocysteinyl residues.^[307]^ Knowing whether or not the bond between selenium and the organic moiety is available to feed the hydrogen selenide pool is critical to understand the potential pharmacological, nutritional, or toxic effects of a given compound. For instance, metabolic evidence for Se−C bond cleavage in diphenyl diselenide was reported in one study. The authors treated Swiss mice with ⁷⁵Se and ^1^⁴C labeled diphenyl diselenide and found labeled metabolites only in urine; in addition, the excretion of ⁷⁵Se and ^1^⁴C was not simultaneous, suggesting the cleavage of the Se−C bond.^[310]^ From the chemical point of view, the Se−C σ‐bond is weaker than the S−C bond and can be broken under mild conditions[^374^, ^375^] or by lyases (participating in the metabolism of S‐containing amino acids or selenocysteine and selenomethionine).^[307]^

The pharmacological action of diselenides *in vivo* could be attributed to their metabolism to HSe^−^, which contributes to the inorganic selenium (iSe) pool and, consequently, could stimulate the selenoproteins biosynthesis.[^161^, ^376^, ^377^] On the other hand, when the selenoprotein synthesis reaches a plateau, the excess of HSe^−^ could be toxic due to the formation of reactive metabolites, such as methylselenol (CH₃SeH) and selenyl‐centered radicals (RSe⋅), which, in the cell milieu, increase the levels of reactive oxygen species (ROS), and, interestingly, might have chemotherapeutic effects.^[378]^

Furthermore, in the literature, the potential metabolism of diselenides to their selenol (−SeH) intermediates, by the action of reductases such as TrxR has been postulated as a crucial step in their antioxidant activity.[^13^, ^14^, ^276^, ^303^, ^304^, ^378^, ^379^, ^380^] However, in oxygen (O₂) rich environments, the contribution of −SeH intermediates may be relatively minor. The direct demonstration of organoselenium compounds metabolism to selenol intermediates in the extra‐ and intracellular environments will require the synthesis of very selenol‐specific probes with selective access to only one of the two cellular environments. In contrast to diphenyl diselenide, the Se atom of the selenenylamide ebselen is not released and thus is not bioavailable.^[281]^

The identification of the main metabolites and the possible release of Se from the organic moiety is important to assess the potential molecular mechanisms of action of organoselenium molecules in the biological environment. Critical aspects for a real pharmacological use of organoselnium compounds are (i) their low selectivity in mimetic reactions, such as the GPx‐like activity; and (ii) the absence of standard models and mechanistic approaches to evaluate their biological activities.

### 4.5 Selenoneine and Selenoneine Diselenide: A Natural Selone–Diselenide Pair

**Figure 9:**
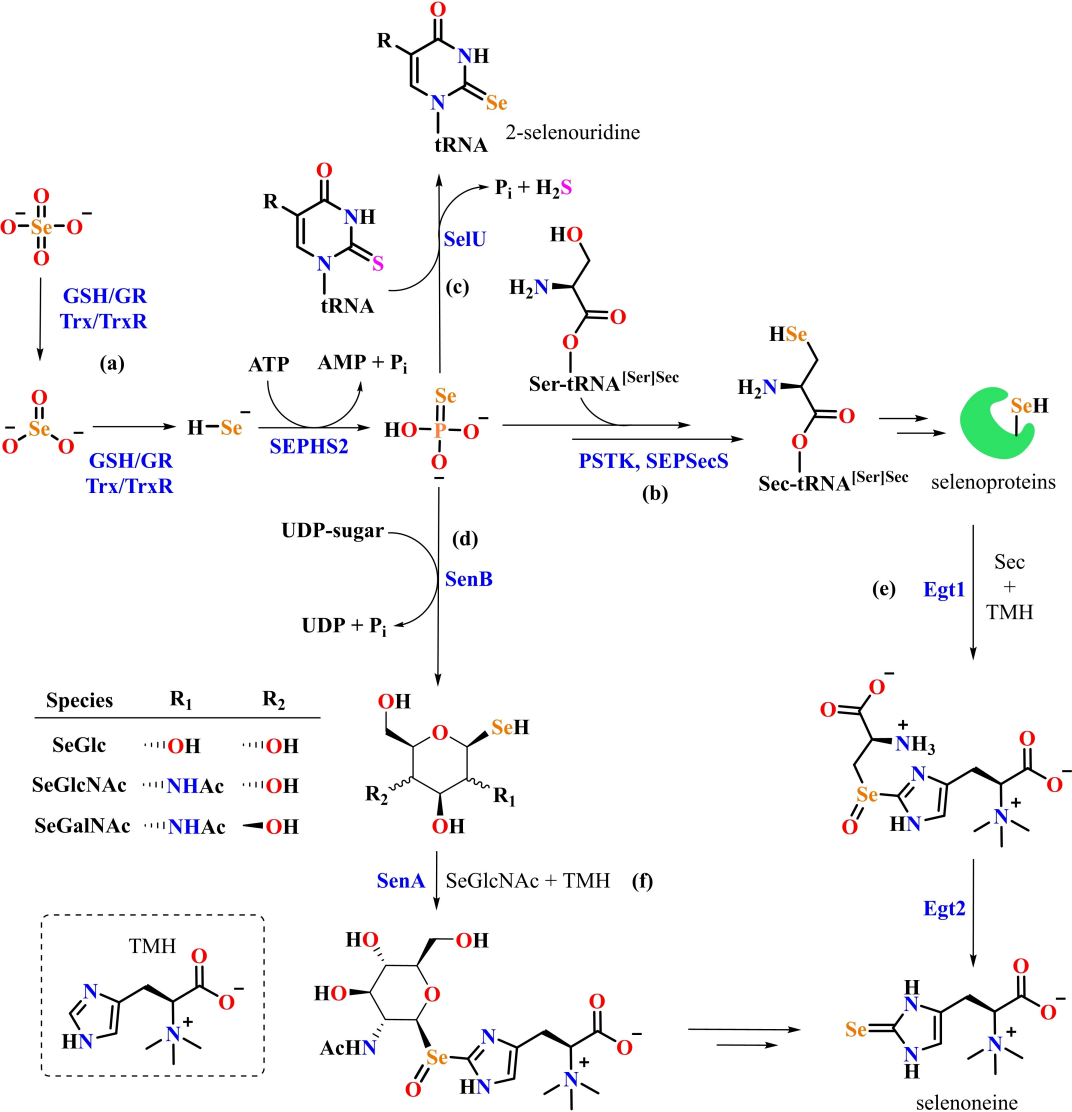
Specific selenium incorporation pathways in eukaryotes. (a) selenate (SeO₄^2−^) and selenite (SeO₃^2−^) are reduced, by GSH/GR and/or Trx/TrxR systems, to selenide (HSe^−^), which gives the selenophosphate (HSePO₃^2−^). The selenophosphate generates the (b) selenoproteins, (c) 2‐selenouridine, and (d) selenosugars, such as selenoglucose (SeGlc), *N*‐acetyl‐selenoglucosamine (SeGlcNAc), and *N*‐acetyl‐selenogalactosamine (SeGlcNAc). The selenoneine synthesis can occur from (e) Sec as a substrate of Etg1, and/or from (f) *N*‐acetyl‐selenoglucosamina (SeGlcNAc) as selenium‐donor in a reaction catalyzed by selenoneine synthase (SenA). In both cases, the trimethylhistidine (TMH) is a substrate. Free Sec can be used as Se source in *S. pombe* and recombinant *Aspergillus*[^387^, ^390^, ^404^]. The specific synthesis of selenoneine is catalyzed by the enzyme SenA, which transfers the Se atom from SeGlcNAc (right side in the Scheme)^[391]^. The product of SenA is a hercynyl‐SeGlcNAc selenoxide intermediate, similar to the hercynylcysteine sulfoxide intermediate from ergothioneine biosynthesis, which spontaneously decomposes (by beta elimination) into selenoneine.^[404]^

Of particular importance to the field of synthetic organoselenium compounds, the discovery of selenoneine (2‐selenoxo‐*N*α, *N*α, *N*α‐trimethyl‐*L*‐histidine; a selone or selenoketone; Figure 9), at millimolar concentrations in the muscle of tuna,[^381^, ^382^, ^383^] carnivorous cetaceans, sea birds and in the blood of fish or cetaceans consuming populations in Japan and Canada,[^384^, ^385^, ^386^] highlighted the existence of a natural low molecular mass organoselenium compound in some food chains. Initially, it was thought that selenoneine was synthesized non‐specifically via the incorporation of Se in place of S in ergothioneine (the thione or thioketone analogous of selenoneine; also known as 2‐mercaptohistidine trimethylbetaine, thioneine, or 2‐thioxo‐*N*α, *N*α, *N*α‐trimethyl‐*L*‐histidine) in bacteria and fungi.[^382^, ^387^, ^388^, ^389^, ^390^] Similarly to ergothioneine, and despite its presence in the blood and tissues of fish, birds and mammals, the synthesis of selenoneine occurs only in bacteria and fungi. Of utmost importance to the organoselenium field, the demonstration of a specific metabolic route of synthesis of selenoneine was reported only recently.^[391]^ Importantly, selenoneine biosynthesis represents the third and the fourth known biochemical pathways for an enzyme catalyzed covalent bond formation between an inorganic selenium and an organic carbon.[^391^, ^392^, ^393^] (Figure 9) The specific incorporation of inorganic Se in a selone‐containing low molecular mass and natural compound supports the idea that life from bacteria to mammals can tolerate, and possibly exploit, the selone‐diselenide redox pair physiologically.

The predominant chemical form of ergothioneine in the intracellular environment is the thione. The thione differs from the thiol group because it has a more positive redox potential (~−60 mV) and a more alkaline pKa (~11.0) than the small intracellular monothiol species (e. g., GSH, coenzyme‐A and cysteine, which have a redox potential around −240 mV and pKa ~9.0).[^394^, ^395^] The chemical reactivity of thione in ergothioneine is lower than that of typical thiols (for instance, GSH and cysteine). Ergothioneine does not react with classical thiol‐reagents (e.g, nitroprusside and 5,5‐dithio‐bis‐(2‐nitrobenzoic acid) or DTNB) at physiological pH values.[^396^, ^397^] In relation to ergothioneine, selenoneine has a much more negative redox potential ~−490 mV, which is similar to that of selenocysteine/selenocystine pair (−488 mV).^[383]^

Consequently, the reactivity of selenoneine resembles more closely that of selenocysteine. Specifically, under aerobic conditions and in aqueous media (for instance, the extra‐ and intracellular media), selenoneine is expected to be oxidized to selenoneine diselenide. Since the redox potential of the extracellular environment is more positive than that of the intracellular one, the plasmatic concentrations of monomeric selenoneine should be lower in the former. However, since the methodologies employed to quantify selenoneine typically include 10 mM of DTT, the selone is considered the circulating form of the compound.[^381^, ^384^, ^385^, ^398^, ^399^]

The concentration of selenoneine in human blood (Table 2) varies from 0 to 0.2 μM,^[398]^ and is much higher in the fish‐eating Japanese population (~0.02–9 μM),^[384]^ or in the Beluga‐eating Inuit population in Canada (~0.6–12 μM).[^385^, ^399^] The levels of ergothioneine in human erythrocytes (~40–400 μM) and in plasma (~0.05–2 μM) are typically higher than that of selenoneine.[^384^, ^385^, ^394^, ^398^, ^399^, ^400^, ^401^]

**Table 2:** Blood concentrations of ergothioneine, selenoneine and their methylated metabolites (adapted from^[385]^).

Molecule	[μM] mean (minimum, maximum)	% methylated metabolite
Ergothioneine	100 (50–150)	nd
S‐methyl‐ergothioneine	0.05 (0.02–0.07)	0.05 (0.03–0.07)
Selenoneine	0.05 (0.02–0.12)	nd
Se‐methyl‐selenoneine	0.003 (0.002–0.006)	5.7(4.8–10.2)

nd: not determined.

The physiological role of ergothioneine and selenoneine in mammals is still unknown and it has been suggested that ergothioneine can be a powerful antioxidant derived from the human diet, according to antioxidant and cytoprotective *in vitro* studies.[^394^, ^402^] But the reactivity of selenoneine is much higher than that of ergothioneine as can be deduced also by the levels of their methylated metabolites. The proportional methylation of the S or Se atom in relation to the thione or selone form is 100 times higher for the latter (the percentage of blood Se‐methylselenoneine is between 4 and 11 %), whereas for the ergothioneine the percentage varies from 0,03 to 0,07 %.[^385^, ^403^] Since the concentration of ergothioneine is usually much higher than that of selenoneine, the methylation may be performed by an enzyme with preference for the selone than the thione group, or due to the Se greater reactivity the reaction rate constant for the methylation of the selone is higher than the methylation of the thione molecule. It is also important to note that, to the best of our knowledge, the actual concentration of selenoneine and its diselenide derivative has not been determined in samples of blood or tissues from vertebrates.

## 5 Summary and Outlook

Roughly fifty years ago, at the beginning of the 1970s, while enzymologists succeeded in bridging the nutritional importance of selenium with molecular biology by discovering the existence of selenoenzymes in both bacteria and mammals, organic chemists observed the synthetic potential of the Se−C bond. With the discovery that organoselenides could be used for the practical manipulation of organic functional groups, the field of modern organoselenium chemistry started. Some ten years later, the field of organoselenium pharmacology started too, with the first identification of a selenium‐based enzyme mimic. From a chemical point of view, organoselenium‐catalyzed oxygen transfers are a class of reactions that provides a bridge between chemistry and biology. Indeed, the first selenoenzyme discovered in mammals, GPx1, precisely catalyzes the oxidation of thiols to disulfides, employing hydrogen peroxide (or, more in general, hydroperoxides) as terminal oxidant. While in biology this reaction takes place to control the concentration of harmful peroxide species as well as for signaling purposes, organic chemists employed organoselenides for the practical activation of hydrogen peroxide, leading to organoselenium‐catalyzed epoxidations, dihydroxilations, Baeyer‐Villiger oxidations and a variety of heteroatom (e. g., S, N, Br) oxidations. In this review, our current mechanistic understanding of these reactions have been summed up. Indeed, the capacity of glutathione peroxidase to reduce hydroperoxides even without a heme‐cofactor has been considered a scientific puzzle since its discovery. Today, the recent and commonly accepted picture sees a double attack on the peroxide bond as the origin of the extreme reactivity of peroxidatic selenocysteine and cysteine residues, with the chalcogen atom being responsible for the nucleophilic attack on one end of the peroxide bond, and a back‐proton transfer from an enzyme residue being responsible for the electrophilic attack to the other end. Much less is known about the reaction mechanisms of other selenoenzymes, and for some of the twentyfive known selenoproteins expressed in humans even their role remains in the dark. Thus, while our understanding of GPx chemistry can be considered a milestone in the field, the road to our understanding of selenium biochemistry is still mostly untravelled.

On the other hand, the exploitation of organoselenides as oxygen‐transfer catalysts in organic chemistry is a well‐developed and explored field. Nevertheless, from the mechanistic point of view, the field somewhat lagged behind the synthetic results. Indeed, the proper identification of the active oxidants in reactions catalyzed by both organoselenium acids and monoselenides proved to be troublesome. Particularly, until very recently, most of our mechanistic understanding was based on the Se(IV) seleninic–peroxyseleninic hypothesis dating back to the mid‐1970s. Conversely, while not completely general, the very recent discovery that Se(VI) selenonic acids can act as hydrogen peroxide activators for epoxidations opens new opportunities and venues for discussion. While many reactions, such as arylamine oxidations, might be properly described by a Se(IV)‐based catalytic cycle, reconsidering the role of Se(VI)‐based catalytic cycles also in other organoselenium‐catalyzed reactions seems a worthwhile pursuing investigation.

The pharmacological application of organoselenium compounds started approximately a decade later, and much effort was devoted to find GPx mimics, i. e., low molecular weight organoselenium compounds displaying catalytic peroxide‐reducing activity. The intrinsic difficulty of this research is mostly due to the high reactivity of the selenol moiety which implies a very poor selectivity, despite interesting attempts of designing molecules with functional elements resembling the protein environment. Ebselen and diphenyl diselenide are still receiving attention, but we are assisting to a paradigm shift in the application of low molecular weight organoselenium compounds, which are now investigated for their thiol modifier potential and their application as antivirals, detoxifying agents and more in general protein inhibitors. But while the importance of low molecular weight organoselenium compounds was going to be scaled back, the recent discovery of selenoneine is fueling the idea that indeed low molecular weight organoselenium compounds may have a biologically active role.

This review, by encompassing different “organoselenium stories” spanning from biochemistry, to chemistry and pharmacology, provides a perspective on our mechanistic understanding of organoselenium chemistry and organoselenides reactivity across different fields, with the aim of inspiring new challenging investigation at the boundaries of these research areas and of building a coherent unified picture of selenium reactivity. We hope this summary of mechanistic information, chemical and biological, to be of help into the design of new experiments in the biological environment as well as the laboratory, pushing the exploitation of organoselenides’ potential even further.

## Conflict of Interests

The authors declare no conflict of interest.

6

## Biographical Information

*Andrea Madabeni was born in Brescia, Italy, in 1996. He obtained his Ph.D. in Molecular Sciences in 2024 under the supervision of Prof. Dr. Laura Orian at Università degli Studi di Padova, with a thesis entitled “A Theoretical Mechanistic Journey into Organoselenium (Bio)chemistry: from Elementary Molecular Models to Catalysis”. At present he is a postdoctoral researcher in the group of Prof. Dr. Orian. His primary research interest revolves around the theoretical investigation of bonding and reaction mechanisms in chemistry, biochemistry and catalysis*.

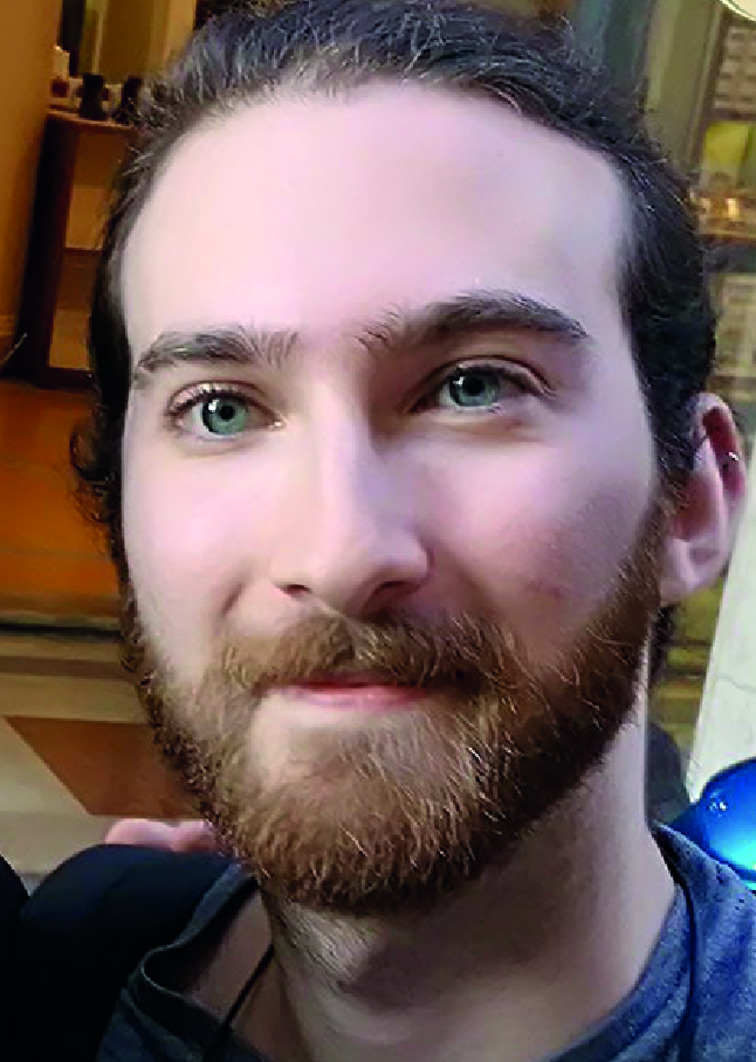

## Biographical Information

*Marco Bortoli was born in Marostica, Italy in 1990. He completed his Ph.D. with Prof. Dr. Laura Orian (University of Padova) and Prof. Dr Matthias Bickelhaupt (Vrije Universiteit Amsterdam), obtaining a joint title in 2019 with a thesis on the role of selenium in glutathione peroxidase. After a postdoc at the Univeristy of Girona under Prof. Dr. Lluís Blancafort on the design of novel compound for neutron cancer therapies, he is currently a Marie Curie postdoc fellow at the University of Oslo in the group of Prof. Dr. Michele Cascella working on the reactivity of main group polar organometallics*.

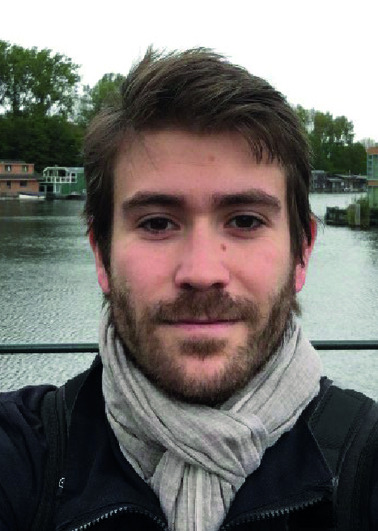

## Biographical Information

*Pablo Andrei Nogara was born in Ijuí, Brazil, in 1991. He graduated in Chemistry from Universidade Federal de Santa Maria (UFSM) in 2014, with an exchange period at Universidad de Oviedo. He obtained his Ph.D. in Biochemistry in 2020 through a sandwich doctoral program between UFSM and Università degli Studi di Padova, supervised by Prof. João B. T. Rocha and Prof. Laura Orian. Currently, he is a professor at Instituto Federal Sul‐rio‐grandense (IFSul). Dr. Nogara's research focuses on mercury and selenium reactions and biological effects, particularly their interactions with thio‐ and selenoproteins, using chemical computational techniques*.

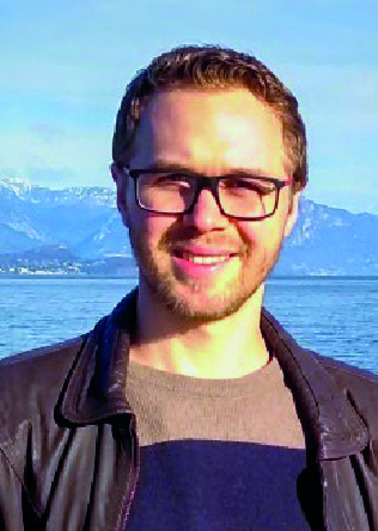

## Biographical Information

*Giovanni Ribaudo was born in Valdobbiadene, Italy, in 1987. He received his Ph.D. in Molecular Sciences in 2015 from Università degli Studi di Padova after carrying out part of the research activity at the State University of New York in Albany, USA. In 2019, he joined Università di Brescia and his research activity is based on the combined use of synthetic, analytical (HPLC, NMR, mass spectrometry) and computational medicinal chemistry tools. The main research topics consist in the design and screening of small molecules interacting with peculiar DNA arrangements and Nature‐inspired drug‐like bioactive compounds*.

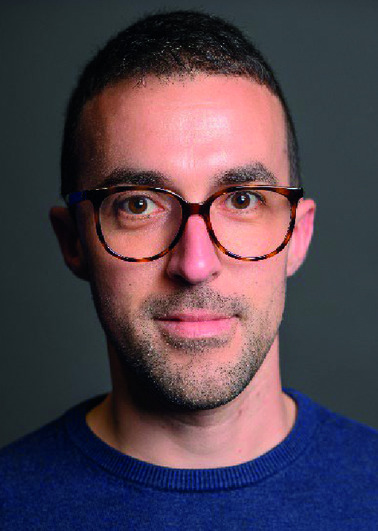

## Biographical Information

*Marco Dalla Tiezza was born in Feltre, Italy, in 1993. He obtained his Ph.D. in Molecular Sciences in 2021 through a joint doctoral program between Università degli Studi di Padova and Vrije Universiteit Amsterdam. His Ph.D. research, conducted under the supervision of Prof. Dr. Laura Orian as part of the REdox state role in Bio‐inspired ELementary reactions (REBEL) project, focused on the theoretical study of inorganic (group 9 and 11 metals) and enzyme catalysis. He completed his Ph.D. with a thesis entitled “A Quantitative Kohn–Sham Approach to Elementary Redox Reactions in Artificial, Bio‐inspired, and Biological Catalysis.”*

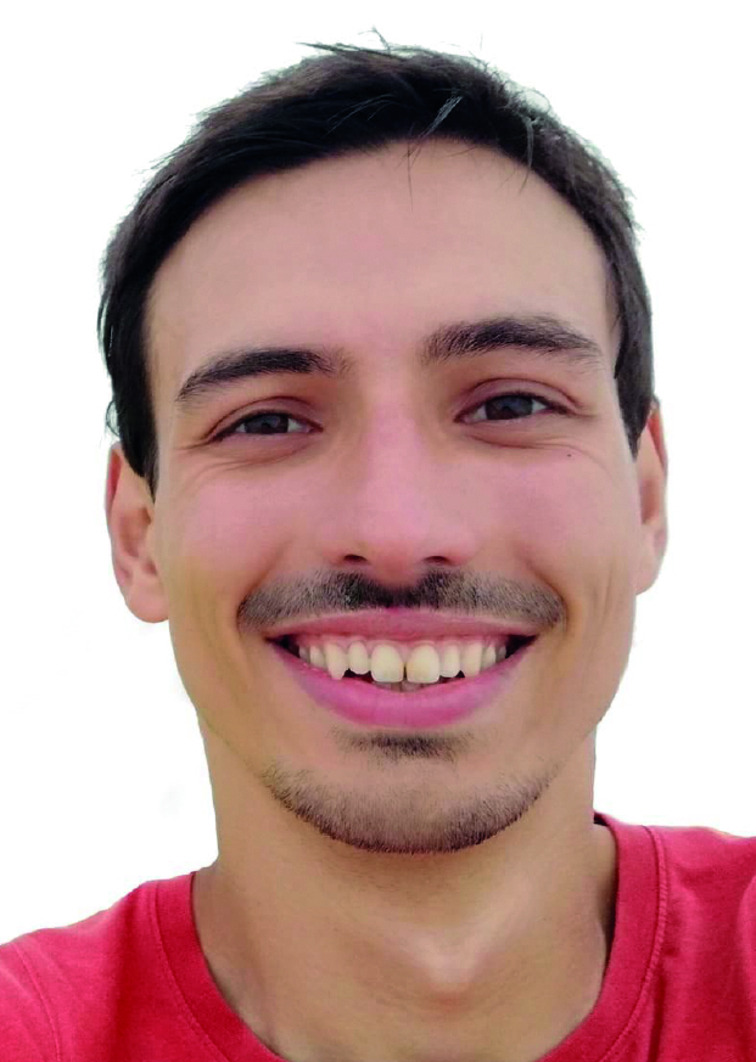

## Biographical Information

*Born in 1938 in Grevenbroich, DE, Leopold Flohé obtained his diploma In 1967 in biochemistry and in 1968 a medical doctorate from the University of Tübingen, DE. From 1976–1990 he served as R&D director at Grünenthal GmbH in Aachen, DE. Thereafter, he became scienific director of the of the GBF (now HZI) in Braunscweig, DE. After 5 years, he decided to switch back to bench‐type reasearch, spent a three months sabatical at the University of Berkeley, CA, and then took the Chair of Biochemisry at the University of Braunschweig. After retirement, he kept working in a start‐up company, as Chairman of the COST Action CM 0801 (EU), as guest professor at the Universities of Magdeburg, DE, Montevideo, UY, and Padova, IT. He received an award from the Anna Monica Foundation (1973), the Claudius Galenus Prize (1985), the Klaus Schwarz Commemorative Medal (1997), the Science and Humanity Prize of the Oxygen Club of California (2001), the Trevor Frank Slater Award and Gold Medal (2006), was elected Redox Pioneer #3 (2010), and received honorary degrees of the universities of Buenos Aires (AR, 1997) and Montevideo (UY, 2013)*.

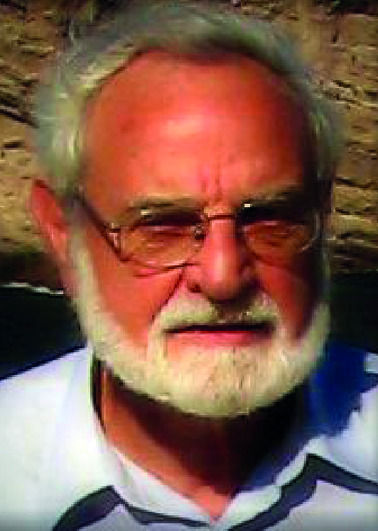

## Biographical Information

*João B.T. Rocha has a degree in Biological Sciences from the Federal University of Rio Grande do Sul ‐ UFRGS (1986), and a PhD in Biological Sciences (Biochemistry) from the Federal University of Rio Grande do Sul ‐ UFRGS (1996). He completed his Post‐Doctorate at UFRJ, Department of Medical Biochemistry in the Bioenergetics Laboratory coordinated by Prof. Leopoldo de Meis (1997–1998). He currently works in the areas of organochalcogen, mercury toxicity, mercury‐thiol and mercury‐Se interactions. He has experience in the neurodevelopmental toxicology of mercury. He participates in activities related to science education (mainly biology and chemistry)*.

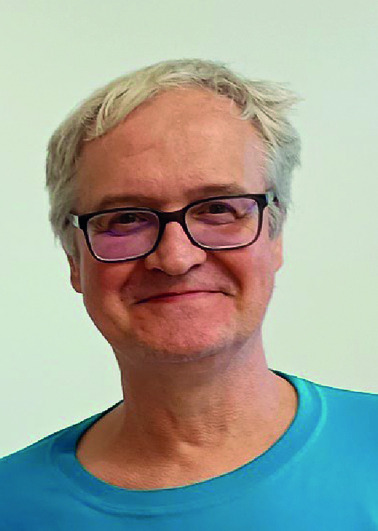

## Biographical Information

*Laura Orian graduated cum laude (1997) and got her PhD in Chemical Sciences (2001) at Universita‘ degli Studi di Padova. She is currently Associate Professor of Physical Chemistry in Padova. Her research aims to elucidate physical‐chemical phenomena, particularly reactivity, rooted in the properties of atoms and (bio)molecules. Her approach to the interpretation of the experiment is pursued by improving the fundamental description of chemical systems (chemical theory) and by applying new and existing techniques to chemical, physical and biological problems (chemical computation). The last goal of her research is to predict the reactivity properties of a chemical system in advance of the experiment, for the rational design of functional molecules assisted by computer. Her favorite and most known modeling activity is in the field of chalcogen redox (bio)chemistry, organoselenium catalysis and reaction mechanisms, and selenoproteins and their biomimics*.

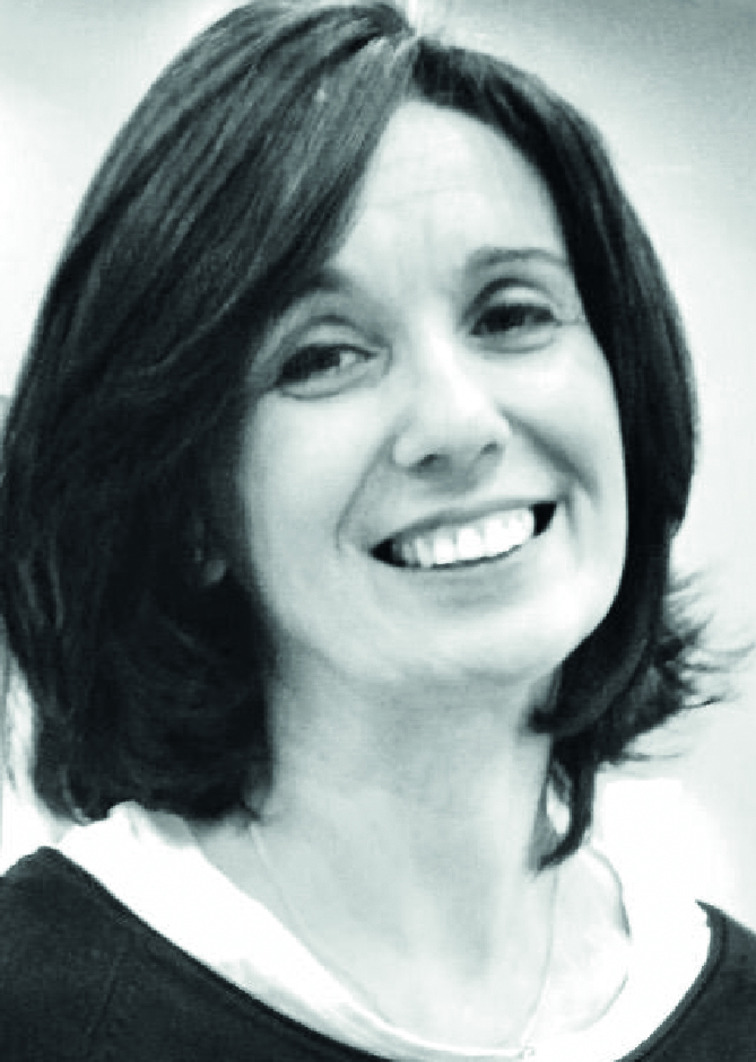

## Data Availability

Data sharing is not applicable to this article as no new data were created or analyzed in this study.
